# Infraslow Closed-Loop Brain Training for Anxiety and Depression (ISAD): A pilot randomised, sham-controlled trial in adult females with internalizing disorders

**DOI:** 10.3758/s13415-025-01279-z

**Published:** 2025-03-18

**Authors:** Tyson M. Perez, Divya B. Adhia, Paul Glue, Jiaxu Zeng, Peter Dillingham, Muhammad S. Navid, Imran K. Niazi, Calvin K. Young, Mark Smith, Dirk De Ridder

**Affiliations:** 1 Department of Surgical Sciences, University of Otago, Dunedin, 9016 New Zealand; 2 Department of Psychological Medicine, University of Otago, Dunedin, New Zealand; 3 Department of Preventative & Social Medicine, Otago Medical School-Dunedin Campus, University of Otago, Dunedin, New Zealand; 4 Coastal People Southern Skies Centre of Research Excellence, Department of Mathematics & Statistics, University of Otago, Dunedin, New Zealand; 5 Centre for Chiropractic Research, New Zealand College of Chiropractic, Auckland, New Zealand; 6 Donders Institute for Brain, Cognition and Behaviour, Radbout University Medical Center, Nijmegen, The Netherlands; 7 Department of Psychology, University of Otago, Dunedin, New Zealand; 8 Neurofeedback Therapy Services of New York, New York, NY USA

**Keywords:** Infraslow neurofeedback, Electroencephalography, Internalizing disorders, Anxiety, Depression, Triple network

## Abstract

**Introduction:**

The core resting-state networks (RSNs) have been shown to be dysfunctional in individuals with internalizing disorders (IDs; e.g., anxiety, depression). Source-localised, closed-loop brain training of infraslow (≤ 0.1 Hz) EEG signals may have the potential to reduce symptoms associated with IDs and restore normal core RSN function.

**Methods:**

We conducted a pilot randomized, double-blind, sham-controlled, parallel-group (3-arm) trial of infraslow neurofeedback (ISF-NFB) in adult females (*n* = 60) with IDs. Primary endpoints, which included the Hospital Anxiety and Depression Scale (HADS) and resting-state EEG activity and connectivity, were measured at baseline and post 6 sessions.

**Results:**

This study found credible evidence of strong nonspecific effects as evidenced by clinically important HADS score improvements (i.e., reductions) across groups. An absence of HADS score change *differences* between the sham and active groups indicated a lack of specific effects. Although there were credible slow (0.2–1.5 Hz) and delta (2–3.5 Hz) band activity reductions in the 1-region ISF-NFB group relative to sham within the targeted region of interest (i.e., posterior cingulate), differences in activity and connectivity modulation in the targeted frequency band of interest (i.e., ISFs = 0.01–0.1 Hz) were lacking between sham and active groups. Credible *positive* associations between changes in HADS depression scores and anterior cingulate cortex slow and delta activity also were found.

**Conclusions:**

Short-term sham and genuine ISF-NFB resulted in rapid, clinically important improvements that were nonspecific in nature and possibly driven by placebo-related mechanisms. Future ISF-NFB trials should consider implementing design modifications that may better induce differential modulation of ISFs between sham and treatment groups, thereby enhancing the potential for specific clinical effects in ID populations.

**Trial Registration:**

The trial was prospectively registered with the Australia New Zealand Clinical Trials Registry (ANZCTR; Trial ID: ACTRN12619001428156).

**Supplementary Information:**

The online version contains supplementary material available at 10.3758/s13415-025-01279-z.

## Introduction

Mental disorders are one of the most common causes of morbidity and mortality worldwide (Kessler et al., 2009) with rates markedly increasing in recent years (Duffy et al., 2019; Haidt & Allen, 2020; Keyes et al., 2019; Pfeifer & Allen, 2020; Twenge et al., 2019). In New Zealand, it is estimated that one in five people suffer from a mental illness at any given time with a majority likely to experience at least one episode in their lifetime (Kris, 2018). Alarmingly, New Zealand’s suicide numbers are increasing; the 2017–2018 rate is the highest in 20 years, which is contributing to a staggering reduction in life expectancy (i.e., up to 25 years) for mental illness sufferers (Kris, 2018). Internalizing disorders (IDs), which include generalized anxiety disorder, social anxiety disorder, and major depressive disorder among others, are the most prevalent psychopathologies experienced worldwide (Demyttenaere et al., 2004; Kessler et al., 2005, 2007; 2009; Oakley-Browne et al., 2006) and can be broadly characterised by a proclivity to direct distress inwardly (Buchan et al., 2014; Carragher et al., 2015; Kotov et al., 2017; Krueger & Eaton, 2015; Rhee et al., 2015). Notably, IDs are highly comorbid (Bui & Fava, 2017; Stein et al., 2017) with females and young people (i.e., < 65 years) disproportionately affected (Kessler et al., 2015).

In recent years, neuropsychiatric research is pointing to transdiagnostic, neurobiological aberrations specifically involving the so-called core resting-state networks (RSNs), which include the default mode network (DMN), central executive network (CEN), and salience network (SN) (Beckmann et al., 2024; Hamilton et al., 2015; Kim & Yoon, 2018; Li et al., 2023; Menon, 2011; Xu et al., 2019). Briefly, the DMN is anchored in the posterior cingulate cortex (PCC), medial prefrontal cortex, and lateral parietal cortex, and putatively subserves internally directed thought (Buckner et al., 2008; Raichle, 2015). The CEN, anchored in the dorsolateral prefrontal cortex (dlPFC) and posterior parietal cortices (PPC), is associated with executive functioning and self-control (Dai et al., 2019; Dosenbach et al., 2008; Elton & Gao, 2014; Liang et al., 2016; Sridharan et al., 2008; Uddin, 2014; Uddin et al., 2011). Lastly, the SN, anchored in the anterior insula (aINS) and dorsal anterior cingulate cortex (dACC), is believed to be important for the detection of salient stimuli and switching between the other core RSNs (Menon, 2015; Seeley et al., 2007). Additionally, the core RSNs have also been associated with autonomic nervous system (ANS) modulation (Beissner et al., 2013; Critchley et al., 2011; Sie et al., 2019) possibly helping to explain the autonomic dysfunction consistently reported across psychopathologies (Alvares et al., 2016; Beauchaine, 2015). In 2011, the converging neurobiological evidence led Menon and colleagues to propose a unifying theory of psychopathology termed the “triple network model” (Menon, 2011). The central tenet of this theory is that the sensorial, cognitive, affective, and behavioural dysfunctions associated with mental illnesses are the result of disruptions within and between the core RSNs (Menon, 2011, 2020). Since its inception, support for this model has been rapidly mounting within the domain of IDs (Janiri et al., 2020; Perez et al., 2022a, 2022b, 2022c; Rayner et al., 2016; Sha et al., 2019; Yang et al., 2019).

Electroencephalography (EEG) noninvasively tracks and records electrophysiological signals generated by the brain (Buzsáki et al., 2012). Although traditionally used to assess activity in sensor-space, modern source-space algorithms (e.g., low-resolution brain electromagnetic tomography, LORETA) now allow accurate estimations of the brain regions (i.e., nodes) responsible for generating the scalp-recorded electrophysiological signals (Aoki et al., 2015; Pascual-Marqui, 2002). Furthermore, although standard clinical EEGs limit the recording bandwidth to traditional frequency bands, acquisition, and analyses of frequencies at the low end of the spectrum, commonly termed electrophysiological infraslow fluctuations (ISFs, ≤ 0.1 Hz), are now possible (Vanhatalo et al., 2005). Russian scientists discovered ISFs more than half a century ago, first in rabbits (Aladjalova, 1957; Aladjalova & Kol'tsova, 1958) and shortly thereafter in humans (Aladjalova, 1964) but, in large part owing to technological challenges, they have received little interest from the scientific and clinical communities until recently (He & Raichle, 2009; Niedermeyer et al., 2011; Smith, 2013; Vanhatalo et al., 2004, 2005). Putatively engendered by a combination of neuronal and glial currents (He & Raichle, 2009; Krishnan et al., 2018; Leistner et al., 2010; Pan et al., 2013; Vanhatalo et al., 2005; Watson, 2018), ISFs have been shown in both cortical (Aladjalova, 1957, 1964; Timofeev et al., 2000) and subcortical (Dash et al., 2018; Hughes et al., 2011; Lőrincz et al., 2009; van Putten et al., 2015; Watson, 2018) tissues and are believed to coordinate RSN organisation and long-range information exchange (Abbas et al., 2019; Buzsáki & Draguhn, 2004; Engel et al., 2013; Florin & Baillet, 2015; He & Raichle, 2009; Palva & Palva, 2012; Weaver et al., 2016). Furthermore, a ~ 0.1 Hz slow wave generator has been described in the dACC which is believed to act as a “central pacemaker” modulating cardiac-related ANS activity (Pfurtscheller et al., 2017). As such, treatments specifically targeting ISFs within core nodes of the triple network may address core RSN dysfunction and offer clinical utility in the treatment of IDs.

Although traditional frontline therapies (i.e., pharmacotherapy & psychotherapy) are effective for many, they offer numerous shortcomings including high failure rates (Choi & Jeon, 2020; Fornaro et al., 2019; Furukawa et al., 2019; Iosifescu, 2011; Nicholson et al., 2020; Perlman et al., 2019; Seibell & Hollander, 2014), lack of access (Andrade et al., 2014; Bandelow & Michaelis, 2015; Haller et al., 2015; Möller et al., 2016; Schoenberg & David, 2014), and marked adverse side-effects (Alvares et al., 2016; Andersohn et al., 2009; Gøtzsche, 2014; Haller et al., 2015; Möller et al., 2016; Pinter et al., 2019). In fact, many pharmaceutical companies are abandoning neuropsychiatric research because of frequent failures of novel agents to show efficacy (Huneke et al., 2020). A recent government inquiry by the New Zealand government has shed light on the shortcomings of current treatment and called for wider implementation of nonpharmaceutical approaches in treatment of mental health problems (Kris, 2018). Similarly, scientists in other parts of the world are calling for research into “novel interventions that may be based on altering plasticity or returning circuitry rather than neurotransmitter pharmacology” (Insel & Wang, 2010).

The implementation of safe, noninvasive neuromodulation techniques that have the potential to impact neuroplasticity within and between large-scale RSNs may offer new treatment opportunities for individuals who do not want, respond to, or tolerate standard interventions. Additionally, these techniques may serve as adjuncts to traditional treatments, potentially enhancing their efficacy. As an example, closed-loop brain training of EEG signals, also known as EEG neurofeedback, is a noninvasive therapy designed to modulate brain function by teaching individuals, via associative learning (e.g., operant conditioning), to self-regulate their brain function via auditory, visual, and/or tactile feedback (Cannon et al., 2009). That said, sceptics assert that comparable clinical improvements in both experimental and control groups in randomized, double-blind, sham-controlled trials suggest that EEG neurofeedback's efficacy rests entirely on nonspecific psychosocial factors (i.e., expectations, motivation, demand characteristics, context) (Arnold et al., 2020; Ghaziri & Thibault, 2019; Schabus et al., 2017; Schönenberg et al., 2017a, 2017b; Thibault & Raz, 2016a, 2016b, 2017; Thibault et al., 2015, 2016, 2017; Thibault et al., 2018a). However, proponents contend that evidence of differential EEG learning (i.e., greater change in the targeted electrophysiological variable(s) and/or region(s)-of-interest (ROIs) in the genuine versus sham groups), considered by many to be essential for a valid evaluation of EEG neurofeedback’s specificity (Arns et al., 2014; Kerson, 2013; Sherlin et al., 2011; Szewczyk et al., 2018; Witte et al., 2018; Zuberer et al., 2015), was noticeably absent in the trials presented as proof of wholly nonspecific effects (Pigott et al., 2018; Trullinger et al., 2019). Assessments of differential EEG learning are complicated, however, by a lack of standardised criteria for the determination of learning (or a lack thereof) (Weber et al., 2020). In any case, EEG neurofeedback has shown promising clinical effects in a wide variety of conditions, including IDs (Askovic et al., 2020; Bell et al., 2019; Chrapusta et al., 2015; Orndorff-Plunkett et al., 2017; Ros et al., 2017; Schoenberg & David, 2014; Van der Kolk et al., 2016).

Clinicians have reported success in ID populations targeting ISFs using sensor-space EEG neurofeedback (Grin-Yatsenko et al., 2018; Othmer & Othmer, 2009; Villanueva et al., 2011). Advanced source-localization (i.e., standardized LORETA, sLORETA (Pascual-Marqui, 2002)) combined with EEG neurofeedback targeting ISFs (ISF-NFB) is a novel introduction to the field that has been shown by our research group in a feasibility trial on obese females to improve sleep and wellbeing with minimal side-effects (Sook Ling Leong et al., 2018a, 2018b; Leong et al., 2018a, 2018b). In addition, we found evidence that ISF-NFB may modulate frequencies above ISFs within the targeted ROIs (Sook Ling Leong et al., 2018a, 2018b). Based on our recently published systematic review (Perez et al., 2022a, 2022b, 2022c), we believe that ours is the first randomized, double-blind, sham-controlled trial examining the effects of source-space ISF-NFB in an ID population.

We undertook this transdiagnostic trial with the following primary goals:

1. To compare the effectiveness of sham versus genuine ISF-NFB for treating IDs in an adult female population. Specifically, we sought to investigate differences between sham ISF-NFB and single-region ISF-NFB (ISF1) or two-region ISF-NFB (ISF2) in patient-reported outcomes (PROs) and EEG variables after 6 training sessions. We hypothesised that all groups would show clinical improvements via nonspecific (e.g., placebo) effects; however, ISF1 and ISF2 groups will demonstrate additional improvements owing to specific effects (i.e., effects specific to the modulation of the trained ROIs).
2. To assess whether there is a relationship between changes in EEG variables and PROs after 6 sessions for sham, ISF1, and ISF2. Specifically, we are interested in whether there is evidence for an association between changes in the primary PRO (i.e., Hospital Anxiety & Depression Scale, HADS) scores and targeted ROI activity and connectivity.

## Materials and methods

### Study population and setting

The targeted population for this investigation is adult females meeting the Diagnostic & Statistical Manual of Mental Disorders (DSM-5) criteria for one or more current IDs of interest. Our trial recruited participants from the community in and around Dunedin, New Zealand, and was undertaken at the Departments of Surgical Sciences and Psychological Medicine, University of Otago, Dunedin, New Zealand.

### Important changes to methods after trial commencement

As noted in our published protocol (Perez et al., 2022a, 2022b, 2022c), recruitment began on February 15, 2020, but was prematurely halted due to COVID-19 lockdown measures in New Zealand. Recruitment efforts resumed on June 15, 2020; however, because of budgetary and time restrictions imposed by the lockdown, we amended our trial design. Specifically, our original recruitment goal for clinical participants was changed from 80 (40 males and 40 females) to 60 females.

### Eligibility Criteria

#### Inclusion criteria

- Able to give informed consent
- 18 to 64 years old
- Biological female
- Meets the DSM-5 criteria for one or more of the following *current* diagnoses:
  o Generalized anxiety disorder
  o Social anxiety disorder
  o Major depressive disorder

#### Exclusion criteria

- Intends to start or alter dosages of psychiatric medications < 4 weeks before their first baseline session or at any time during the study.
- Currently taking short-acting benzodiazepines (e.g., midazolam)
- Any externalizing disorder (e.g., substance abuse disorder)
- Any thought disorder (e.g., bipolar disorder)
- Any neurological disorder (e.g., epilepsy)
- Deemed to be at high-risk of suicide per the Columbia-Suicide Severity Rating Scale (C-SSRS)
- Pregnant
- Pacemaker
- Post-concussion syndrome

#### Recruitment

Recruitment took place during the period of February 2020 to July 2021. Participants were recruited via posters and Facebook ads with an invitation to participate in a University of Otago mental health study. Advertisements directed potential participants to a webpage that described the study and invited those interested to complete a short online form, which queried basic information, including first name, age, date of birth, sex, ethnicity, education level, handedness, mental health history, pregnancy status, presence of electronic implants (i.e., pacemakers), email address, and phone number. Individuals who completed the online form and met the basic qualifications were contacted via email and were asked to attend an in-person mental health interview at the University of Otago Hospital, Dunedin, New Zealand. Those who agreed were provided directions to the lab and a digital copy of the 7-page participant information sheet. A reminder text was sent to potential participants on the day of their interview. All participants who completed the study received a NZ$40 supermarket voucher as reimbursement for any parking expenses. Trial recruitment continued until the randomisation goal (i.e., *n* = 60) was met.

#### Screening

Following the attainment of informed consent, a trained male PhD student conducted the Mini-International Neuropsychiatric Interview (MINI; English version 7.0.2 for DSM-5) (Sheehan et al., 1998). The MINI is a brief structured diagnostic interview, shown to be both valid and reliable, used to assess the 17 most common psychiatric disorders, including major depression, suicidality, bipolar, panic disorder, agoraphobia, social anxiety, obsessive compulsive disorder, posttraumatic stress disorder, alcohol use disorder, substance use disorder, psychoses, anorexia, bulimia, binge-eating disorder, generalized anxiety, and antisocial personality disorder (Lecrubier et al., 1997; Sheehan et al., 1997). If the interviewer suspected that the candidate was at high-risk for suicide per the MINI, he screened them using the C-SSRS (Posner et al., 2011) with affirmative answers to questions 4, 5, and/or 6b initiating immediate referral to emergency psychiatric services. Those meeting the eligibility criteria were enrolled into the study, had their anthropometric (i.e., height and weight) measurements taken, and were scheduled for their baseline assessments. Participants were also familiarized with the study equipment, procedures, and personnel.

#### Baseline assessments

Baseline assessments took place on two separate occasions approximately 1 week apart with baseline #2 used as reference. Duplicate baseline assessments were performed to mitigate the influence of certain nonspecific effects (i.e., regression to the mean & elevation bias) (Barnett et al., 2004; Shrout et al., 2018) that may confound studies. All assessment sessions for a given subject occurred at approximately the same time of day and were led by a female research assistant. Before each assessment session, participants were asked to abstain from 1) food and water for 2 h; 2) smoking/vaping for 8 h; and 3) strenuous exercise, alcohol, caffeine, and over-the-counter medication for 24 h. A reminder email and text were sent to each participant 1 day before and on the day of the assessment sessions, respectively. Adherence to lifestyle restrictions was queried at the beginning of each session with any breaches recorded. In cases of serious breaches (e.g., consumption of alcohol in the prior 24 h), asses sessions were rescheduled. In addition, they were asked to use the toilet immediately prior to testing to ensure an empty bladder.

Patient-reported outcomes (English versions) were recreated in digital form via Qualtrics (Qualtrics, 2005–2020), which allowed participants to complete them by using an iPad during their EEG setup. To prevent missing data, a visual alert was generated if any queries on a given form had missing responses. Research has indicated the electronic data collection increases the speed, accuracy, and user acceptability of the process (Dale & Hagen, 2007; Lane et al., 2006; Litchfield et al., 2005). The estimated total time to complete the battery of PROs was 20 min.

Following completion of their PROs, neurophysiological data were collected from each participant by using Compumedics Neuroscan SynAmps RT 64-channel amplifier (DC mode, input impedance > 10 GΩ, 24-bit analog-to-digital resolution, common mode rejection > 110 dB) using a continuous sampling rate of 1000 Hz. Recordings took place in a quiet, cool (~ 15 °C), dimly lit room as participants were seated upright in a comfortable chair with their eyes closed. The 10.5-min resting-state broad-band EEGs used high-density (64-channel) silicone Quik-Cap Hydro Net caps with Ag/AgCl electrode placements corresponding to the international extended 10/20 system. The ground electrode is positioned at AFz with the reference electrode midway between Cz and CPz. The cap was soaked in a saline solution at least 30 min prior to application, and all electrode impedances were kept below 10 kΩ. To help reduce impedances, subjects were asked to arrive with nonbraided, dry, clean (i.e., no conditioner, gels, pastes, sprays) hair.

#### Randomisation

Following baseline #2 measurements, participants were randomised to one of three arms: 1) sham = 6 sessions of yoked-sham; 2) one-region ISF-NFB (ISF1) = 6 sessions PCC up-training; 2) two-region ISF-NFB (ISF2) = 6 sessions of co-modulation (i.e., PCC up-training + dACC down-training).

#### Sequence generation

The randomization scheme was generated by using the website Randomization.com by a lab member from our group with no direct contact with the participants. Block randomization with random block sizes and a 2:1:1 (sham: ISF1: ISF2) allocation was utilised.

#### Concealment mechanism

Randomisation sequences were kept in the central office in sequentially number, sealed, opaque envelopes prepared by the lab member who generated the randomisation scheme. To ensure concealment, the block sizes were known only by this lab member and not disclosed to any of the researchers who had contact with the participants.

#### Implementation

Author TMP was responsible for participant enrolment and allocated participants to interventions following baseline assessments and upon arrival at their first ISF-NFB session.

#### Blinding

This is a double-blind study whereby participants and assessors were unaware of group assignments. The ISF-NFB trainer was not blinded. To improve participant blinding, all aspects of sham sessions were identical to genuine sessions including the live recording of sham participants’ EEGs along with real-time artefact alerts. Treatment assignment was disclosed to trial participants upon their completion of the study.

#### Interventions

ISF-NFB sessions commenced within 1-week after baseline #2 assessments. Participants attended three 30-min sessions per week, every other day, over 2 consecutive weeks (6 sessions in total). To help reduce impedances, subjects were asked to arrive with nonbraided, dry, clean (i.e., no conditioner, gels, pastes, sprays) hair. A 19-channel sLORETA ISF-NFB training was performed by using a DC coupled amplifier produced by Brainmaster Inc. and the BrainAvatar software (version 4.7.5.844) in a quiet, cool (~ 15 °C), dimly lit room by a male researcher with > 2 years of experience in the administration of EEG-NFB. Participants were seated in a comfortable chair and an appropriately sized Comby EEG cap was placed on their head. Using a blunt needle and syringe, the scalp was gently abraded prior to the application of an electrolyte gel beneath each electrode. It should be noted that the purpose of the cool room and scalp abrasions was to mitigate contamination of the EEG signal by electrodermal (i.e., sweat gland) potentials, which are known to mimic brain derived ISFs (Niedermeyer et al., 2011; Vanhatalo et al., 2005). 19-channel EEGs were recorded with the silver/silver chloride (Ag/AgCl) electrodes positioned according to the International 10–20 system (i.e., Fp1, Fp2, F3, F4, C3, C4, P3, P4, O1, O2, F7, F8, T3, T4, T5, T6, Fz, Cz, Pz) using a linked mastoids reference and a ground electrode positioned centrally between, F3, Fp1, Fz, and Fpz. The impedances of the active electrodes were kept below 10 kΩ and a 50-Hz notch filter was set.

Immediately prior to each training period, a demonstration of motion artefact alerts was performed with instructions to avoid eye/head/face movements to minimize this nonrewarding feedback. Participants were then instructed to close their eyes, relax, stay awake, and listen to the sound being played. They were informed that the sound that they will hear reflects that they are doing well. Notably, no explicit strategies or instructions were given as, with few exceptions (Scharnowski et al., 2015), implicit strategies have been shown to produce better outcomes (Gruzelier, 2014; Kober et al., 2013; Micoulaud-Franchi et al., 2015; Sepulveda et al., 2016; Strehl, 2014; Sulzer et al., 2013).

Continuous, real-time auditory feedback was used for reinforcement and produced within 30 ms of the subject’s ISFs (0.0–0.1 Hz) within the targeted ROIs (i.e., DMN’s PCC & SN’s dACC) surpassing the threshold(s). These ROIs are considered key cortical nodes within “opposing” (i.e., task-positive vs task-negative) core RSNs, which are consistently found to be disrupted in ID populations. sLORETA permits the selection of any cortical region for feedback of the current density using voxels selected based on Montreal Neurological Institute (MNI) coordinates (Vanneste et al., 2018).

The reward threshold(s) were manually adjusted in real-time to maintain a 60% ± 10% success rate. Manual, rather than automated, thresholding was chosen as it has been reported to lead to better EEG learning (Arns et al., 2017; Micoulaud-Franchi et al., 2015; Sherlin et al., 2011; Strehl, 2014). The yoked-sham sessions were identical to active sessions, including live EEG recordings and real-time motion/EMG artifact alerts; however, the auditory feedback heard by participants was simply a replay of previously recorded trainings of genuine ISF-NFB sessions from another female ID participant at the same training stage recorded via free, open-source Audacity software (Audacity, 1999–2020), which uses the computer’s sound card as an audio to digital converter. Importantly, this type of control allows matching of rewards and performance across sham and genuine conditions, thereby controlling as much as possible the learning context and degree of motivation (Sorger et al., 2019) while theoretically severing the operant conditioning aspect of EEG neurofeedback. Additionally, it has been reported that training effects are more robust when the clinician is present (Cannon, 2012); therefore, irrespective of group assignment, the trainer was present for the duration of all sessions. A detailed description of the trial intervention using the Template for Intervention Description and Replication (TIDieR) (Hoffmann et al., 2014) has been provided in the Supplement.

#### Participant timeline

The trial period for each participant was approximately 4 weeks and consisted of one 30-min screening interview, two 60-min baseline assessments approximately 1 week apart, six 30-min sham or active ISF-NFB sessions (3 × per week over 2 consecutive weeks) starting within 1 week after baseline #2, and a 60-min after 6 session assessment (Table 1). See Supplement for a complete table of trial activities including secondary endpoints. All posttreatment assessments and procedures were identical to those performed at baseline.

**Table 1:** Schedule of trial activities

Trial Activities	T0	T1	T2	T3-8	T9
**Enrolment**					
Informed consent	√				
Eligibility screen (MINI)	√				
Anthropometric measures	√				
**Primary Outcome Assessments**					
HADS		√	√		√
EEG		√	√		√
**Interventions**					
Active ISF-NFB				√	
Sham ISF-NFB				√	
**Safety Monitoring**					
DESS				√	√

T0 = initial interview; T1 = baseline #1; T2 = baseline #2; T3-8 = training sessions 1 through 6; T9 = post 6 session assessments

#### Criteria for discontinuing the trial

Participants were advised that they could withdraw at any time without giving a reason or be withdrawn by the lead investigator if they 1) experienced significant adverse effects that were deemed detrimental to their well-being, or 2) were unable to adhere to protocol (e.g., missed > 1 training session, started, or modified first-line therapies).

#### Strategies to improve adherence to interventions

We attempted to mitigate adherence issues via automated email and text message reminders sent on the day of each training session.

#### Plans to promote participant retention and follow-up

Once enroled, every reasonable effort was made to follow participants throughout the entirety of the study period via ongoing email and text messaging correspondence. In the event of premature discontinuation of the study for any reason, participants were made aware that all data collected up to the point of withdrawal may be used for analyses.

#### Relevant concomitant care permitted or prohibited during the trial

Participants were asked to maintain any current first-line mental health therapies (e.g., pharmacotherapy) for the entire length of the trial period. Any changes or introductions of first-line therapies (e.g., altered pharmacotherapy dosages, introduction of intensive psychotherapy) rendered participants ineligible.

## Outcomes

### Primary Outcomes

#### Hospital Anxiety and Depression Scale (HADS)

The HADS is a valid and reliable 14-item, transdiagnostic PRO measure used to assess anxiety and depression severities (Zigmond & Snaith, 1983). Response options are on a 4-point scale (0–3) based on participants experiences over the past week with anxiety and depression subscale scores graded as follows: 0–7 = normal; 8–10 = mild; 11–14 = moderate; 15–21 = severe (Breeman et al., 2015). The HADS has been repeatedly shown to be a reliable and valid tool across a variety of settings (Bjelland et al., 2002; Herrmann, 1997; Snaith, 2003). The minimum clinically important difference (MCID) for both HADS subscales is estimated to be a reduction > 1.5 points (Lemay et al., 2019; Puhan et al., 2008; Wynne et al., 2019). That said, to our knowledge, the MCID has not been formally established in populations with MDD, GAD, and/or SAD; therefore, our MCID is derived from values established within other populations and the clinical experience of our team’s psychiatrist (i.e., PG).

#### Broad-band EEG

EEG pre-processing was performed offline using EEGLAB version 14.1.1 (Delorme & Makeig, 2004) and ERPLAB version 6.1.4 (Lopez-Calderon & Luck, 2014) running on MATLAB 2021a (The MathWorks, Inc., Natick, MA). Custom scripts developed in MATLAB utilizing EEGLAB, ERPLAB, and MATLAB functions were used. The raw EEG were imported into MATLAB by using EEGLAB. Channel locations/coordinates were determined via EEGLAB’s *Montreal Neurological Institute (MNI) coordinate file for BEM dipfit model* with the head centre optimized. Non-EEG (i.e., VEOG, HEOG, EKG, EMG, GSR) and four EEG (i.e., F11, FT11, F12, FT12) channels were removed prior to manual coregistration used to match the coordinates of the 60 remaining channels to the realistic Boundary Element Model (MNI) head model*.* Of note, the four EEG channels selected for removal lack locations in the MNI coordinate file, thereby precluding subsequent pre-processing. The data were then truncated to retain only the middle 600 s of the time series, and the PREP pipeline version 0.55.1 (Bigdely-Shamlo et al., 2015) was run to identify and interpolate bad channels, remove line noise, and robust average reference the data. This pipeline has been used previously for evoked potentials and resting-state EEG data (Navid et al., 2019, 2020). PREPed EEGs with > 25% (i.e., > 15) bad channels identified were not considered for further analyses. For identification and marking of artifact-contaminated epochs, continuous PREPed EEGs were 1-Hz high-pass filtered by using a finite impulse response (FIR) filter implemented using EEGLAB’s pop_firwsord function (window = Kaiser 5.653, transition bandwidth = 1.5 Hz, max ripple = 0.001, order = 2416) and segmented into 1-s epochs. Epochs were automatically marked as artifacts if containing one or more of the following characteristics: (i) absolute voltage exceeds 100 µV, (ii) peak-to-peak voltage exceeds 150 µV in any sliding window of 200 ms width with a step size of 100 ms, (iii) voltage greater than 100 µV resulting from a step-function with a sliding window 200 ms wide with a step size of 50 ms, (iv) sample-to-sample difference exceeding 50 µV, or (v) absolute voltage less than 1 µV for 150 ms (i.e., flat-lined data). Following this, manual verification/correction of epoch classifications (i.e., artifact and nonartifact) was performed. Manually verified time series with > 50% (i.e., > 5 min) artifact-contaminated epochs were excluded from further analyses.

**Fig. 1:**
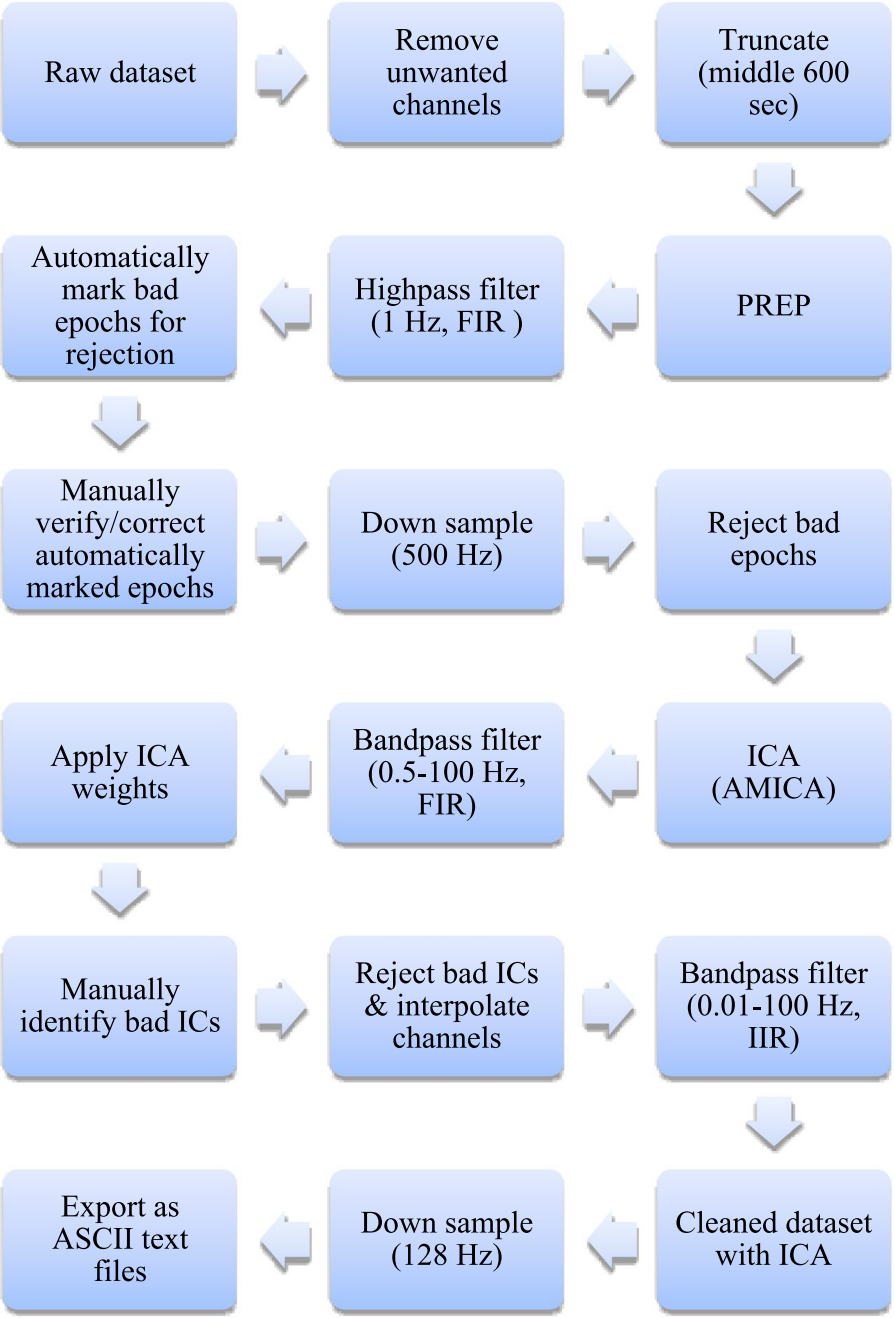
EEG pre-processing pipeline. PREP = early-stage EEG preprocessing pipeline; FIR = finite impulse response; IIR = infinite impulse response; IC = independent component; ICA = IC analysis; AMICA = adaptive mixture ICA; Hz = hertz

For independent component analysis (ICA), the data were again 1-Hz high-pass FIR filtered (window = Kaiser 5.653, transition bandwidth = 1.5 Hz, max ripple = 0.001, order = 2416), downsampled to 500 Hz to reduce computation time, had noisy channels and artifact-contaminated epochs removed, and decomposed into maximally independent components (ICs), which are spatially fixed and temporally discrete (Onton & Makeig, 2009) using adaptive mixture ICA (AMICA) (Palmer et al., 2011). AMICA was selected based on its superior performance compared with other ICA algorithms (Delorme et al., 2012). The resulting ICA weights were applied to 0.5–100 Hz band-pass FIR filtered (window = Kaiser 5.653, transition bandwidth = 1 Hz, max ripple = 0.001, order = 3624) data. We used ICLabel (Pion-Tonachini et al., 2019) with manual verification to categorize the ICs as brain or other (i.e., muscle, eye, channel noise, line noise, or other) based on their spatial distribution (scalp topography), time course, spectrograms, event-related potential (ERP) images, and current dipole models per guidelines published in the literature (Chaumon et al., 2015) and on the website https://labeling.ucsd.edu/. Finally, bad ICs were removed, noisy channels interpolated, and the data 0.01–100 Hz bandpass infinite impulse response (IIR) filtered (first-order Butterworth) to give cleaned datasets. This IIR filter has been utilized in previous studies of ISFs (de Goede & van Putten, 2019; van Putten et al., 2015). Finally, cleaned datasets with ICA were downsampled to 128 Hz to reduced computation time and were exported to ASCII text files for subsequent analyses. Figure 1 shows an overview of the EEG pre-processing pipeline.

**Fig. 2:**
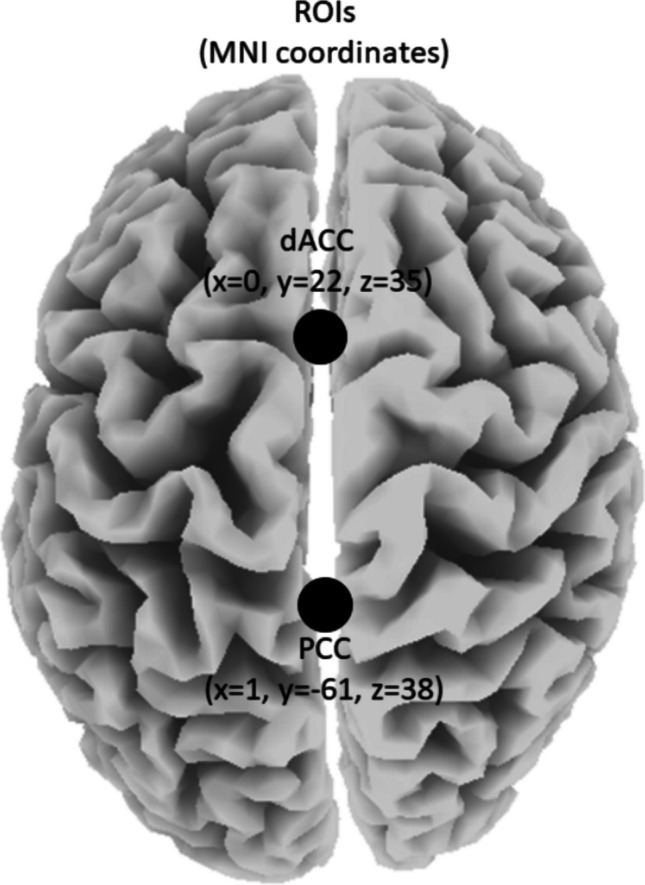
Regions-of-interest (ROIs) and their centre of mass coordinates. MNI = Montreal Neurological Institute; dACC = dorsal anterior cingulate cortex; PCC = posterior cingulate cortex

Via LORETA-KEY software (v20210701; available at: www.uzh.ch/keyinst/loreta.htm), ASCII text files were used as input to compute cross-spectral matrices for each participant for seven frequency bands (infraslow 0.01–0.1 Hz; slow 0.2–1.5 Hz; delta 2–3.5 Hz; theta 4–7.5 Hz; alpha 8–12 Hz; beta 12.5–30 Hz; gamma 30.5–44 Hz) utilizing fast Fourier transform (FFT). To allow for two complete cycles of the lowest frequency of interest (i.e., 0.01 Hz) and to obtain smooth power spectral density, EEGs were segmented into 200-s epochs with Hanning (Hann) tapered windows applied. The cross-spectral matrices was then averaged for each subject and used as input to exact LORETA (eLORETA) to compute whole-brain current source density (CSD; A/m^2^) without assuming a predefined number of active sources (Pascual-Marqui, 2007; Pascual-Marqui et al., 2011). Using the MNI-152 (Montreal Neurological Institute, Canada) template, eLORETA produces an inverse solution space consisting of 6239 cortical grey matter voxels at 5-mm resolution and has been shown to produce exact, zero-error localizations even in the presence of measurement and structured biological noise. eLORETA performs voxel-by-voxel between-condition comparisons of the CSD distribution. Statistical nonparametric mapping was performed for each contrast using built-in voxel-wise randomization test (5000 permutations) to calculate the empirical probability distribution for the max-statistic (e.g., the maximum of a *t* or an *F* statistic) under the null hypothesis while correcting for multiple testing (i.e., for the collection of tests performed for all electrodes and/or voxels, and for all time samples and/or discrete frequencies). For each contrast, the voxel-level, two-tailed max-statistic was used as input to LORETA-KEY software to identify and visualize differences/changes in log-CSD for each of the seven frequency bands. Furthermore, for each condition, log-CSDs were averaged across all voxels within a 10-mm radius of centre of mass MNI coordinates of the targeted ROIs (Fig. 2). This output was exported to Excel (version 2112) and analysed in R version 4.0.5 (Team, 2020) to identify differences/changes in log-CSD between contrasts for each ROI in each frequency band. Next, functional connectivity (FC) differences/changes between the targeted ROIs were calculated for each group in LORETA-Key using linear lagged connectivity (Pascual-Marqui, 2007). Output was then exported to Excel and analysed in R to identify differences/changes between contrasts in each frequency band.

#### Secondary outcomes

See Supplement for descriptions of secondary endpoints of interest.

#### Outcome amendments

During the initial design, the primary PROs included the HADS, MEDI, and IDAS-II; however, after discussions with the trial team, we decided to make the MEDI and IDAS-II secondary outcomes while keeping the HADS as primary given that it was the only PRO with an established MCID (Lemay et al., 2019; Puhan et al., 2008; Wynne et al., 2019). This amendment occurred prior to the start of data analyses.

#### Sample size

The sample size was chosen for pragmatic reasons (i.e., resource constraints, including limited time and funding). This is the first study examining the effects of sLORETA ISF-NFB in an ID population. Because of its novelty, there was no existing information around standard deviations for the measurements of interest. Therefore, no formal sample size or power calculations were made. Our group has previously performed an sLORETA ISF-NFB trial in obese females (Sook Ling Leong et al., 2018a, 2018b); however, this is a different population than the one in our trial and therefore not considered comparable for this study.

#### Statistical considerations

##### Objective 1:

Between-group (sham vs. ISF1 & ISF2) comparisons were performed for all endpoints. For these analyses, we used LORETA-Key software and a Bayesian model with random effects to allow for baseline differences and non-specific temporal effects between participants.

##### Objective 2:

Regression methods were used to explore the relationship between changes in the primary PRO subscales (i.e., HADS-A, HADS-D) and targeted ROI activity and connectivity.

Analyses were performed by using LORETA-KEY software, R, JAGS (Plummer, 2003), and Stan (Stan Development, 2021) with analysis-specific details described below. For all endpoints, responses were modelled assuming a normal distribution (e.g., with group-specific means & variances or via regression). When normality assumptions were not met, appropriate transformations were performed. Additionally, potential endpoint covariates were examined (e.g., age); however, none exhibited correlations that were strong enough to warrant inclusion for denoising purposes in the models.

For the Bayesian analysis, JAGS or Stan was linked to R using the rjags or rstanarm library with estimates based on 3 chains of 25,000 iterations with a burn-in/warm-up = 10,000 iterations. Vague priors were used throughout. The describe_posterior() function in the bayestest R package (Makowski et al., 2019b) was used to generate posterior summary statistics, including the distribution mean (*M*), 95% credible interval (i.e., highest density interval [HDI]), probability of direction (pd), and the percentage of the *full* posterior within the region-of-practical-equivalence (% in ROPE).

- The HDI is the range of parameter values with a higher probability density than values outside the HDI (Kruschke, 2018). As such, a 95% HDI can be interpreted as a 95% probability that the true (unknown) estimate lies within the interval, given the observed data and priors (Hespanhol et al., 2019).
- The pd is an index of the *existence* of an effect represented by the certainty (50–100%) in the direction, positive or negative (Makowski et al., 2019a, 2019b). For pd interpretation, the following reference values have been suggested:
-  ≤ 95% = uncertain
-  > 95% = possibly existing
-  > 97% = likely existing
-  > 99% = probably existing
-  > 99.9% = certainly existing

The “% in ROPE” indexes the *magnitude* of an effect where the ROPE is the range of effect size values considered to be practically equivalent to the null (Makowski et al., 2019a, 2019b). There is no uniquely correct ROPE, however, by convention, the ROPE range is often set at half the size of Cohen’s definition of small effect size (i.e., 0.2) (Cohen, 1988), resulting in ROPE values of ± 0.1 for *standardised* mean differences (i.e., Cohen’s d) and ± 0.05 for *standardised* regression coefficients (i.e., sβ) (Kruschke, 2018). Cohen’s d values can be interpreted as follows: d <  ± 0.10 = negligible, ± 0.10 < d <  ± 0.20 = very small, ± 0.20 < d <  ± 0.50 = small, ± 0.50 < d <  ± 0.80 = medium, and d >  ± 0.80 large (Cohen, 1988), whereas sβs can be interpreted as follows: sβ <  ± 0.20 = weak association, ± 0.20 < sβ <  ± 0.50 = moderate association, sβ >  ± 0.50 = strong association (Acock, 2016). Regarding “% in ROPE” interpretation, the following reference values have been suggested:

o  > 99% = negligible
o  > 97.5% = probably negligible
o  ≤ 97.5 & ≥ 2.5% = undecided
o  < 2.5% = probably significant
o  < 1% = certainly significant

For all outputs, checks for the validity of assumptions regarding the residuals (i.e., normal distribution, constant & equal variances), chain convergence (e.g., trace plots), and posterior predictive model fit (e.g., Bayesian *p*-value) (Gelman, 2005) were performed.

## Methods to handle missing data and protocol nonadherence

As this was an efficacy trial (i.e., to determine whether ISF-NFB works under ideal conditions), we utilised complete-case analysis for all endpoints and objectives. For data to be included in the analysis, participants were required to attend a minimum of 5 of 6 ISF-NFB sessions. We reported the number and percentages of dropouts/exclusions in each of the groups. Based on our lab group’s previous feasibility study using ISF-NFB in an obese female population (Sook Ling Leong et al., 2018a, 2018b), discontinuation/loss to follow-up following randomization was anticipated to be 10–15%.

## Adverse event reporting and harms

We systematically monitored adverse effects from the therapy for the duration of the trial using the Discontinuation-Emergent Signs and Symptoms checklist (DESS) (Rosenbaum et al., 1998) created verbatim in Qualtrics and completed by participants on an iPad during EEG set-ups in the interventional and postinterventional phases. The DESS is a structured 43-item self-report that utilises the following scale: 1 = new symptom; 2 = old symptom but worse; 3 = old symptom but improved; 4 = old symptom but unchanged; 5 = symptom not present. Participants were informed that they could be withdrawn from the trial by the lead investigator, even without their request, in the event of serious adverse effects.

## Results

### Enrolment

**Fig. 3:**
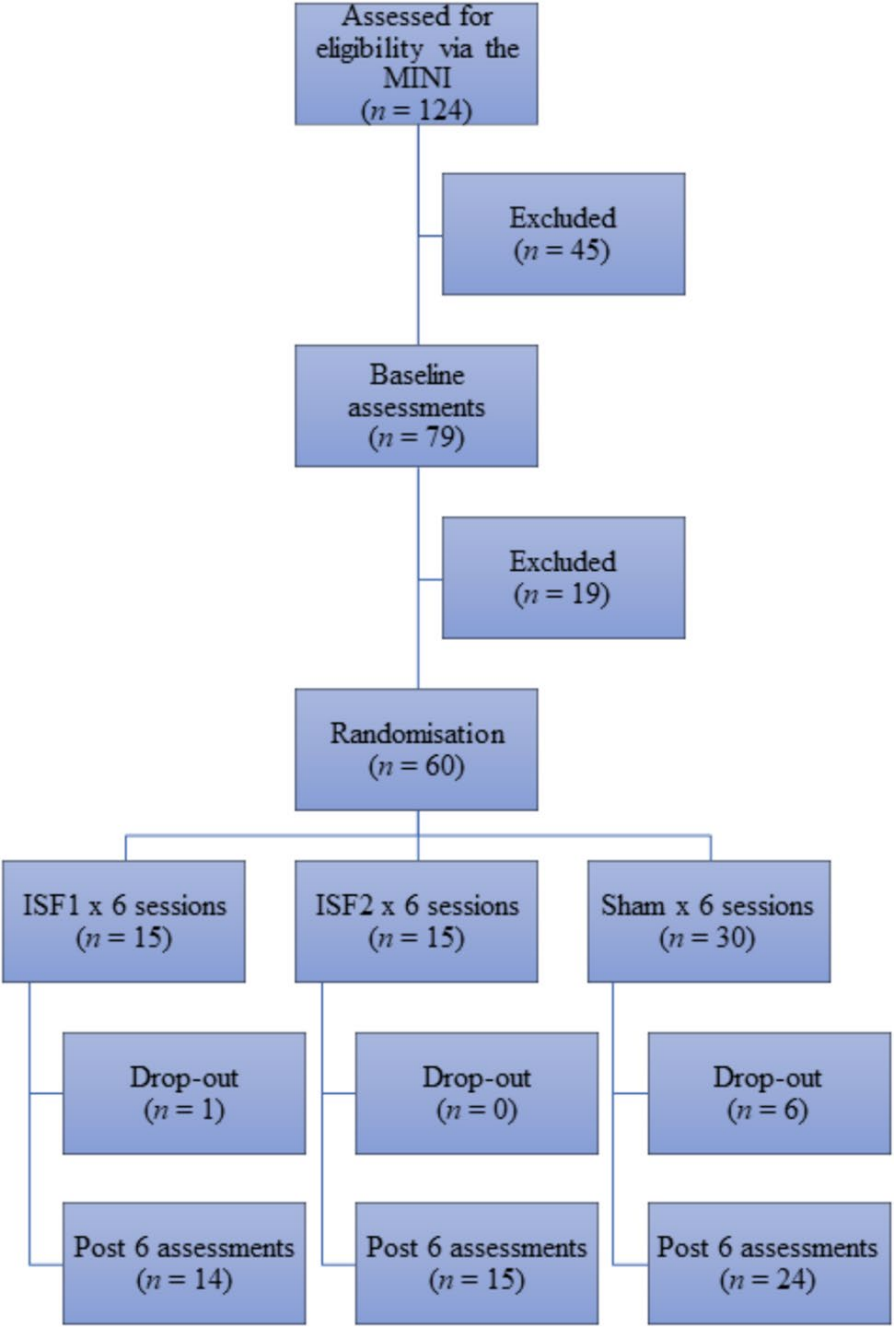
CONSORT participants. MINI = Mini-International Neuropsychiatric Interview; ISF1 = 1-region infraslow neurofeedback; ISF2 = 2-region infraslow neurofeedback

Figure 3 provides an overview of enrolments, assessments, and interventions for the clinical trial. Of the 124 subjects assessed for eligibility via the MINI, 45 were excluded for various reasons, including declining to participate (*n* = 14), failure to attend post interview baseline assessments (*n* = 5), recent change in psychiatric medications (*n* = 1), failure to meet criteria for any ID of interest (*n* = 9), or meeting the criteria for an externalizing/thought disorder (*n* = 16). A total of 79 ID participants underwent baseline assessments with 19 subsequent exclusions due to a variety of factors, including violation of the trial protocol (i.e., starting a new medication; *n* = 1), failure to schedule post baseline ISF-NFB sessions (*n* = 5), and declining to participate (*n* = 13). Notably, all 13 subjects in the latter group reported a change in life circumstances following COVID-19 related lockdown measures as the reason for discontinuation. Ultimately, 60 subjects underwent randomization. A total of seven randomized subjects (11.7%) failed to complete the trial. Reasons given for discontinuation from sham group included lack of time (*n* = 1), lack of benefit (*n* = 1), lack of motivation (*n* = 1), illness (*n* = 1), and adverse side-effects (*n* = 1). Additionally, one sham subject was excluded by the lead trialist owing to a protocol violation (i.e., cessation of medications). The one dropout from ISF1 reported “personal issues” (*n* = 1) as the reason for discontinuation. All subjects randomized to ISF2 completed the trial.

### Demographic and clinical data

A total of 60 participants were included in the trial. Table 2 provides a group-wise overview of the demographic and clinical data of the randomized ID participants. See supplement for baseline data related to secondary outcomes of interest.

**Table 2:** Baseline demographic and clinical characteristics of ID participants by group assignment

Demographic and clinical variables	Sham *n* = 30	ISF1 *n* = 15	ISF2 *n* = 15
Age, years*	34.8 (14.5)	30.7 (11.0)	28.1 (8.6)
**BMI, kg/m**^2^*	28.5 (5.4)	30.5 (10.0)	27.9 (7.4)
Education**			
Secondary/high school	13 (43.3%)	8 (53.3%)	4 (26.7%)
Undergraduate	7 (23.3%)	5 (33.3%)	5 (33.3%)
Postgraduate/professional	10 (33.3%)	2 (13.3%)	6 (40.0%)
Ethnicity**			
European/Pākehā	27 (90.0%)	13 (86.7%)	12 (80.0%)
Māori	1 (3.3%)	1 (6.7%)	0 (0.0%)
Other	2 (6.7%)	1 (6.7%)	3 (20.0%)
Handedness**			
Left	6 (20.0%)	0 (0.0%)	2 (13.3%)
Right	24 (80.0%)	15 (100.0%)	13 (86.7%)
Smoker/vaper**			
No	26 (86.7%)	14 (93.3%)	14 (93.3%)
Yes	4 (13.3%)	1 (6.7%)	1 (6.7%)
Medical comorbidities**			
No	15 (50.0%)	5 (33.3%)	7 (46.7%)
Yes	15 (50.0%)	10 (66.7%)	8 (53.3%)
Psychiatric medications**			
No	15 (50.0%)	5 (33.3%)	8 (53.3%)
Yes	15 (50.0%)	10 (66.7%)	7 (46.7%)
Major depressive disorder**			
Current	8 (26.7%)	6 (40.0%)	8 (53.3%)
Past	22 (73.3%)	9 (60.0%)	7 (46.7%)
Generalized anxiety disorder**			
No	4 (13.3%)	2 (13.3%)	3 (20.0%)
Yes	26 (86.7%)	13 (86.7%)	12 (80.0%)
Social anxiety disorder**			
No	8 (26.7%)	3 (20.0%)	6 (40.0%)
Yes	22 (73.3%)	12 (80.0%)	9 (60.0%)
Agoraphobia**			
No	24 (80.0%)	12 (80.0%)	12 (80.0%)
Yes	6 (20.0%)	3 (20.0%)	3 (20.0%)
Anorexia/bulimia**			
No	30 (100.0%)	15 (100.0%)	15 (100.0%)
Yes	0 (0.0%)	0 (0.0%)	0 (0.0%)
Obsessive compulsive disorder**			
No	29 (96.7%)	15 (100.0%)	14 (93.3%)
Yes	1 (3.3%)	0 (0.0%)	1 (6.7%)
Panic disorder**			
Current	13 (43.3%)	4 (26.7%)	6 (40.0%)
Lifetime	5 (16.7%)	4 (26.7%)	2 (13.3%)
Never	12 (40.0%)	7 (46.7%)	7 (46.7%)
Posttraumatic stress disorder**			
No	28 (93.3%)	8 (53.3%)	12 (80.0%)
Yes	2 (6.7%)	7 (46.7%)	3 (20.0%)
HADS***			
Anxiety {0–21}	10.8 (4.0; 17)	11.9 (3.8; 13)	12.2 (3.5; 12)
Depression {0–21}	7.9 (4.1; 14)	10.1 (3.9; 13)	9.5 (5.1; 18)

*Mean (standard deviation); **n (percentage); ***Mean (standard deviation; range)

### Primary endpoints

#### Patient-reported outcomes

To assess PRO change scores and change difference scores from baseline to post-6 sessions, a Bayesian model was implemented to estimate posteriors for each subscale. Mean change scores were derived by calculating the within-group average difference between baseline #2 and post-6 sessions scores. Tables 3 and 4 show summary statistics for the primary PRO subscales (i.e., HADS-A & HADS-D) and include the group-wise posterior mean effect size (i.e., Cohen’s d) of the change scores (d) with 95% HDI, the posterior mean Cohen’s d of the difference in change scores (d difference) with 95% HDI, probability of direction (pd) of the d and d difference, and the percentage in ± 0.1 region of practical equivalence (% in ± 0.1 ROPE) of the full d and d difference posterior distributions.

**Table 3:**
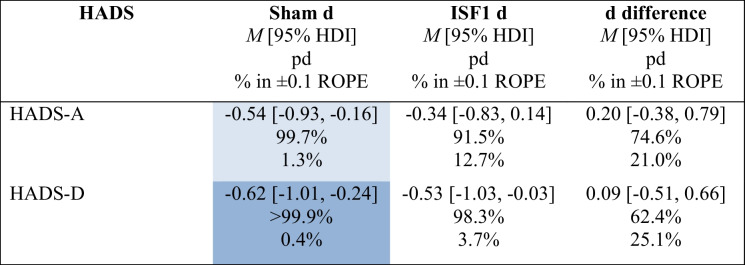
Hospital Anxiety & Depression Scale (HADS) subscale mean changes and change differences for sham and ISF1

Light blue cell = probably significant reduction; Dark blue cell = certainly significant reduction; HADS-A = anxiety subscale; HADS-D = depression subscale; *M* = mean; HDI = highest density interval; pd = probability of direction; % in ± 0.1 ROPE = percentage of the posterior in the ± 0.1 region of practical equivalence; d = Cohen’s d

**Table 4:**
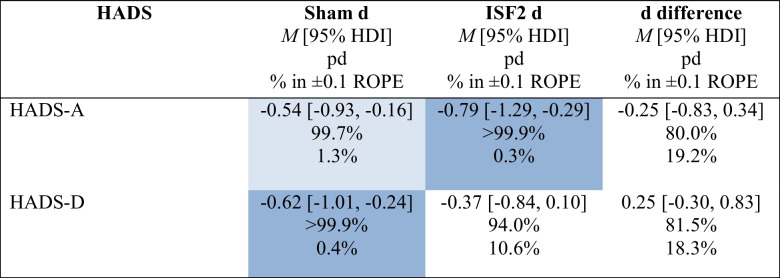
Hospital Anxiety & Depression Scale (HADS) subscale mean changes and change differences for sham and ISF2

Light blue cell = probably significant reduction; Dark blue cell = certainly significant reduction; HADS-A = anxiety subscale; HADS-D = depression subscale; *M* = mean; HDI = highest density interval; pd = probability of direction; % in ± 0.1 ROPE = percentage of the posterior in the ± 0.1 region of practical equivalence; d = Cohen’s d

**Fig. 4:**
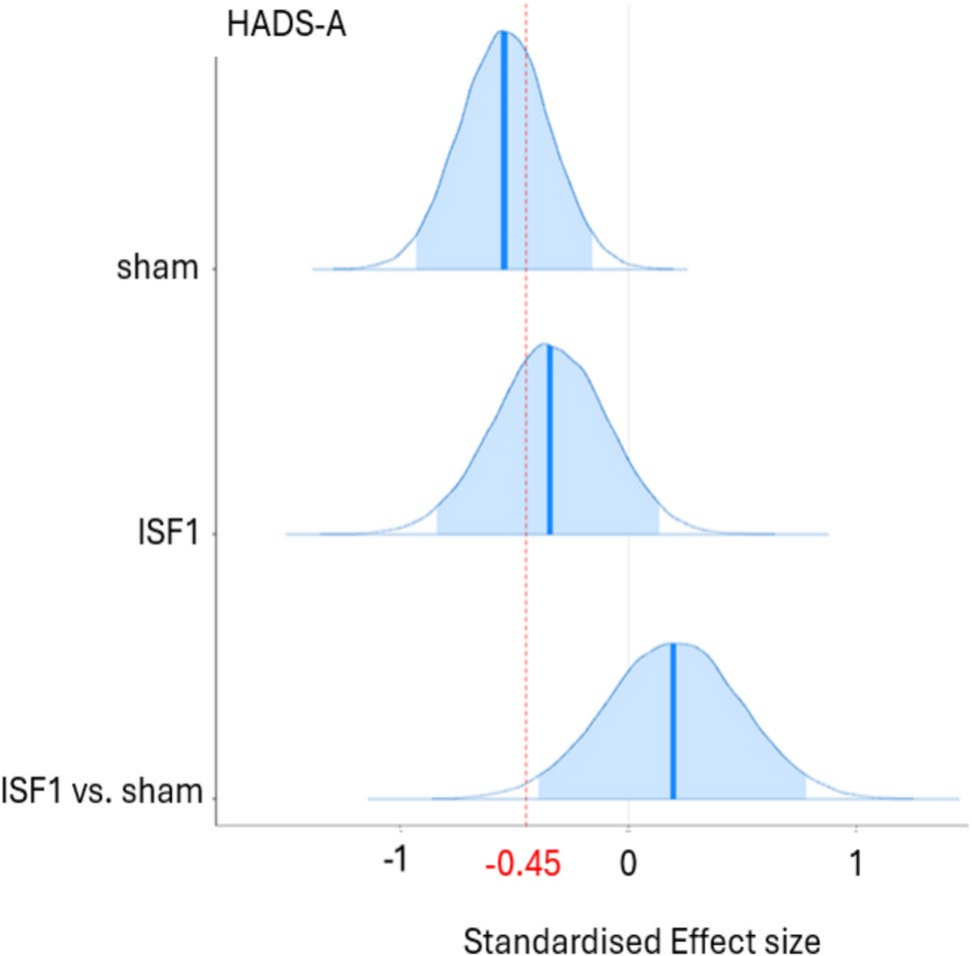
Hospital Anxiety & Depression Scale—Anxiety Subscale (HADS-A) Posterior Cohen’s d & d Difference for Sham & ISF1. *Note.* ISF1 = 1-region infraslow neurofeedback. Dark blue line = distribution mean; Light blue shading = 95% highest density interval (HDI); Red dotted line = standardised minimum clinically important difference (MCID)

**Fig. 5:**
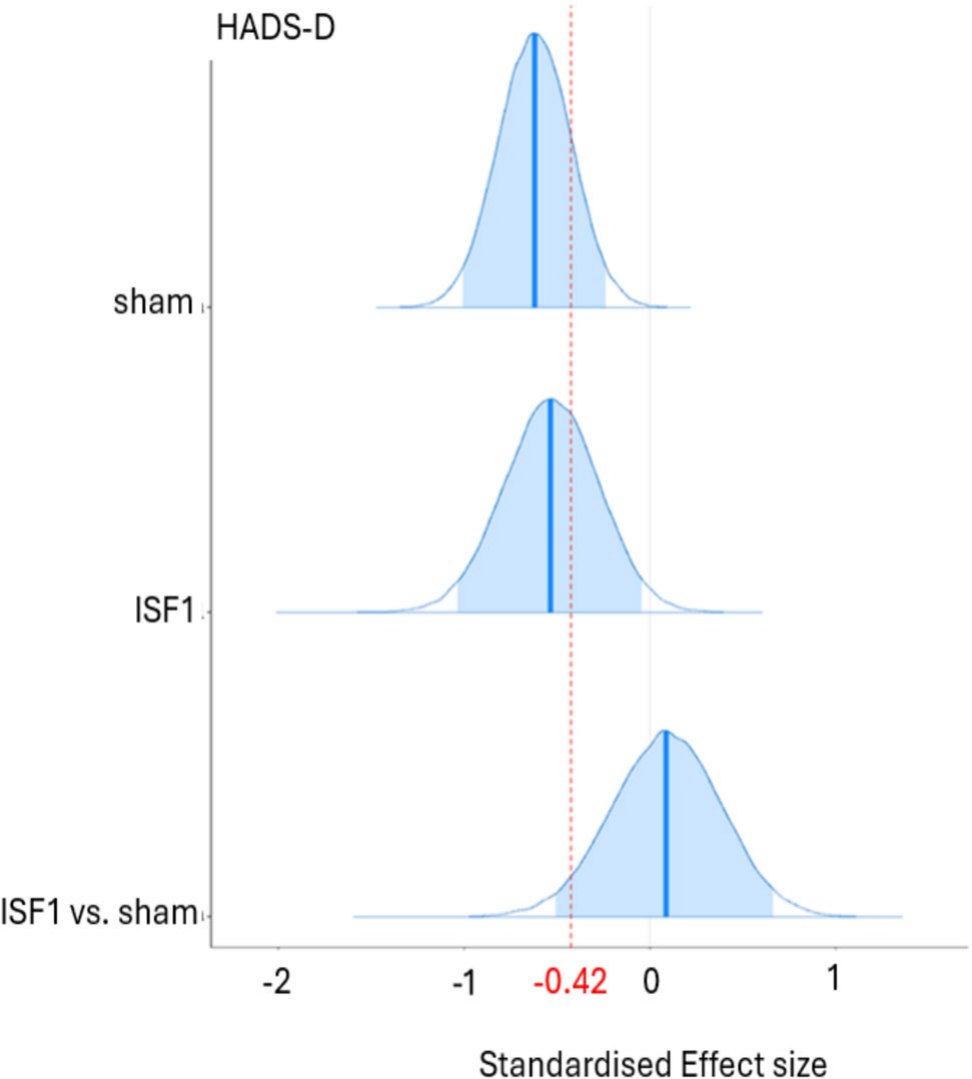
Hospital Anxiety & Depression Scale—Depression Subscale (HADS-D) Posterior Cohen’s d & d Difference for Sham & ISF1. *Note.* ISF1 = 1-region infraslow neurofeedback; Dark blue line = distribution mean; Light blue shading = 95% highest density interval (HDI); Red dotted line = standardised minimum clinically important difference (MCID)

As shown in Tables 3 and 4, the existence of *negative* change scores for sham is probable (HADS-A: 99.7% pd) and certain (HADS-D: > 99.9% pd) with point estimates suggesting medium effect sizes (HADS-A: − 0.54 d [95% HDI, − 0.93 to − 0.16]; HADS-D: − 0.62 d [95% HDI, − 1.01 to − 0.24]), which are probably (HADS-A: 1.3% in ROPE) and certainly (HADS-D: 0.4% in ROPE) significant. For ISF1, the existence of *negative* change scores is uncertain (HADS-A: 91.5% pd) and likely (HADS-D: 98.3% pd) with point estimates suggesting small (HADS-A: − 0.34 d [95% HDI, − 0.83 to 0.14]) and medium (HADS-D: − 0.53 d [95% HDI, − 1.03 to − 0.03]) effect sizes with undecided significances (HADS-A: 12.7% in ROPE; HADS-D: 3.7% in ROPE). For ISF1 vs. sham, the existence of change score *differences* is uncertain (HADS-A: 74.6% pd, HADS-D: 62.4% pd) with point estimates suggesting small (HADS-A: 0.20 d [95% HDI, − 0.38 to 0.79]) and negligible (HADS-D: 0.09 d [95% HDI, − 0.51 to 0.66]) effect sizes with undecided significances (HADS-A: 21.0% in ROPE; HADS-D: 25.1% in ROPE). Plots of the group-wise (sham & ISF1) posterior d and d difference are shown in Figs. 4 and 5. As shown, the sham group’s HADS-A and HADS-D point estimates exceeded the standardised MCID. For ISF1, only the HADS-D point estimate exceeded this threshold. Per published guidance (Lakens, 2013), standardised MCIDs were derived via the division of the MCID (i.e., − 1.5) by the standard deviation of the subscale. Graphical depictions of the pd and % ± 0.1 ROPE of the d difference for both the HADS-A and HADS-D are provided in the Supplement.

**Fig. 6:**
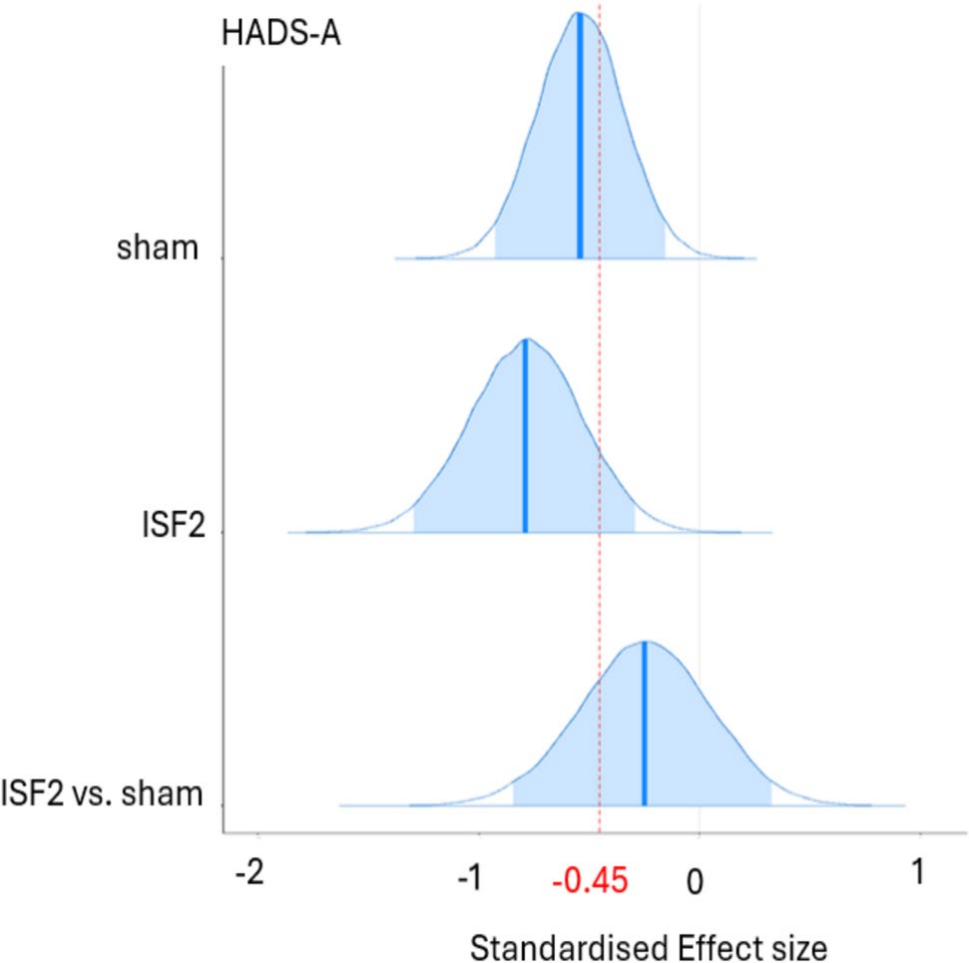
Hospital Anxiety & Depression Scale—Anxiety Subscale (HADS-A) Posterior Cohen’s d & d Difference for Sham & ISF2. *Note.* ISF2 = 2-region infraslow neurofeedback. Dark blue line = distribution mean. Light blue shading = 95% highest density interval (HDI). Red dotted line = standardised minimum clinically important difference (MCID)

**Fig. 7:**
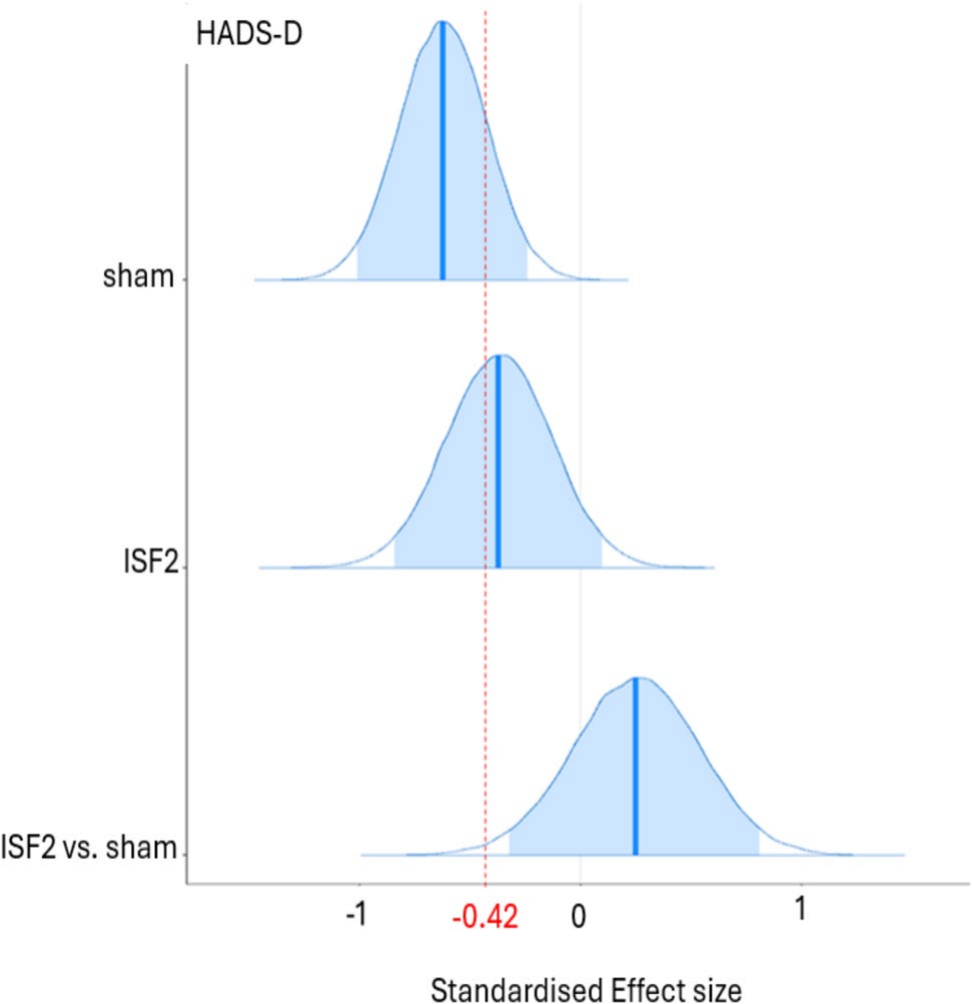
Hospital Anxiety & Depression Scale – Depression Subscale (HADS-D) Posterior Cohen’s d & d Difference for Sham & ISF2. ISF2 = 2-region infraslow neurofeedback; Dark blue line = distribution mean; Light blue shading = 95% highest density interval (HDI); Red dotted line = standardised minimum clinically important difference (MCID)

As shown in Table 4, the existence of *negative* change scores for ISF2 is certain (HADS-A: > 99.9% pd) and uncertain (HADS-D: 94.0% pd) with point estimates suggesting medium (HADS-A: − 0.79 d [95% HDI, − 1.29 to − 0.29]) and small (HADS-D: − 0.37 d [95% HDI, − 0.84 to 0.10]) effect sizes with certain (HADS-A: 0.3% in ROPE) and undecided (HADS-D: 10.6% in ROPE) significances. For ISF2 vs. sham, the existence of change score *differences* is uncertain (HADS-A: 80.0% pd, HADS-D: 81.5% pd) with point estimates suggesting small effect sizes (HADS-A: − 0.25 d [95% HDI, − 0.83 to 0.34]; HADS-D: 0.25 d [95% HDI, − 0.30 to 0.83]) with undecided significances (HADS-A: 19.2% in ROPE; HADS-D: 18.3% in ROPE). Plots of the group-wise (sham & ISF2) d and d difference are shown in Figs. 6 and 7. As shown, the sham group’s HADS-A and HADS-D point estimates exceeded standardised MCID. For ISF2, only the HADS-A point estimate exceeded this threshold. The Graphical depictions of the pd and % ± 0.1 ROPE of the d difference for both the HADS-A and HADS-D are provided in the Supplement.

#### Whole-brain activity

**Fig. 8:**
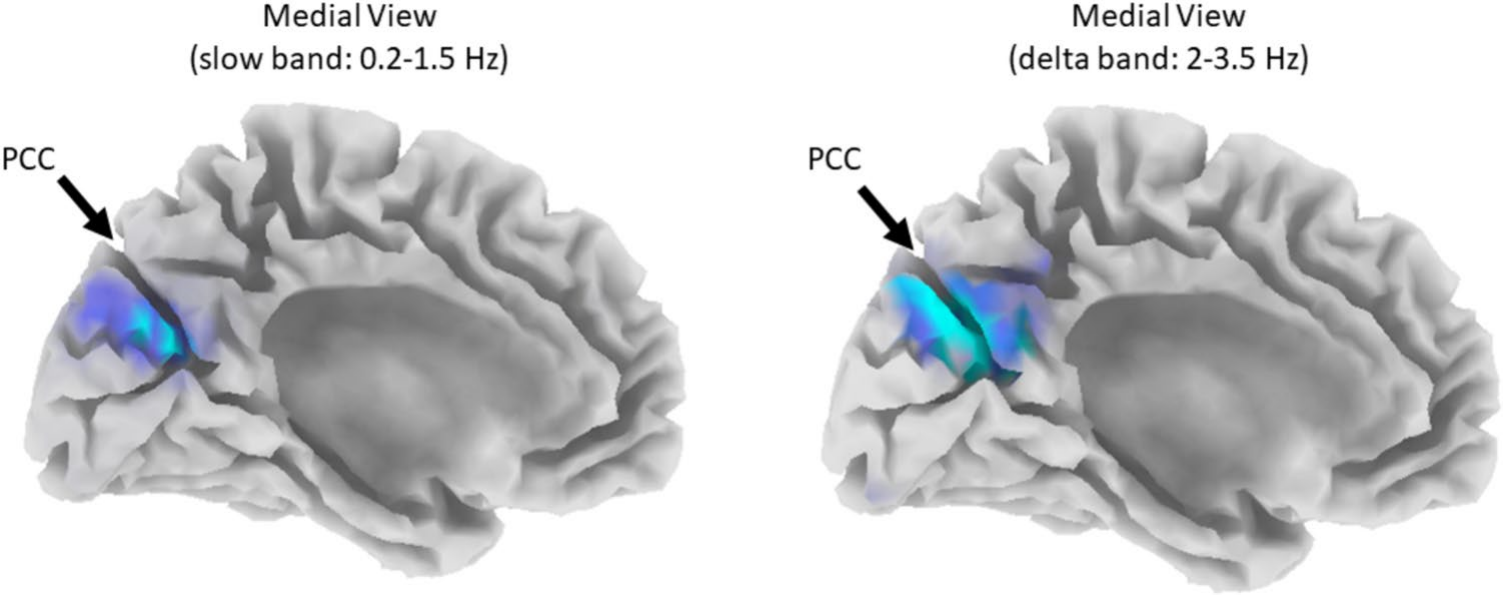
Whole-brain activity (log-CSD) changes in ISF1 compared with sham. ISF1 = 1-region infraslow neurofeedback; PCC = posterior cingulate cortex; Cyan-blue colour = reduced activity

To assess change differences in whole-brain activity from baseline to post-6 sessions, we utilized LORETA-KEY software to perform whole-brain, voxel-wise estimations of the differences in log-CSD changes in the infraslow (0.01–0.1 Hz), slow (0.2–1.5 Hz), delta (2–3.5 Hz), theta (4–7.5 Hz), alpha (8–12 Hz), beta (12.5–30 Hz), and gamma (30.5–44 Hz) frequency bands. As shown in Fig. 8, there is strong evidence (t(39) = 4.2, *p* = 0.008) for activity change differences between sham and ISF1 in the slow and delta bands centred at the targeted ROI (i.e., PCC; slow: x = 0, y =  − 75, z = 25; delta: x =  − 10, y =  − 70, z = 25). In contrast, for sham vs. ISF2, there is weak evidence (t(40) = 4.0, *p* = 0.509) for activity change differences in all frequency bands.

#### Region-of-Interest (ROI) activity

To assess change and change differences in the activity of the targeted Region-of-Interest (ROI) ROIs (i.e., dACC & PCC) from baseline to post-6 sessions, a Bayesian model was used to estimate posterior distributions for the infraslow (0.01–0.1 Hz), slow (0.2–1.5 Hz), delta (2–3.5 Hz), theta (4–7.5 Hz), alpha (8–12 Hz), beta (12.5–30 Hz), and gamma (30.5–44 Hz) frequency bands. Tables 5 and 6 show summary statistics and include the group-wise posterior mean effect size (i.e., Cohen’s d) of the log-CSD changes (d), the posterior mean effect sizes of the difference in log-CSD changes (d difference), probability of direction (pd) of the d and d difference, and the percentage in ± 0.1 region of practical equivalence (% in ± 0.1 ROPE) of the full d and d difference posterior distributions.

**Table 5:**
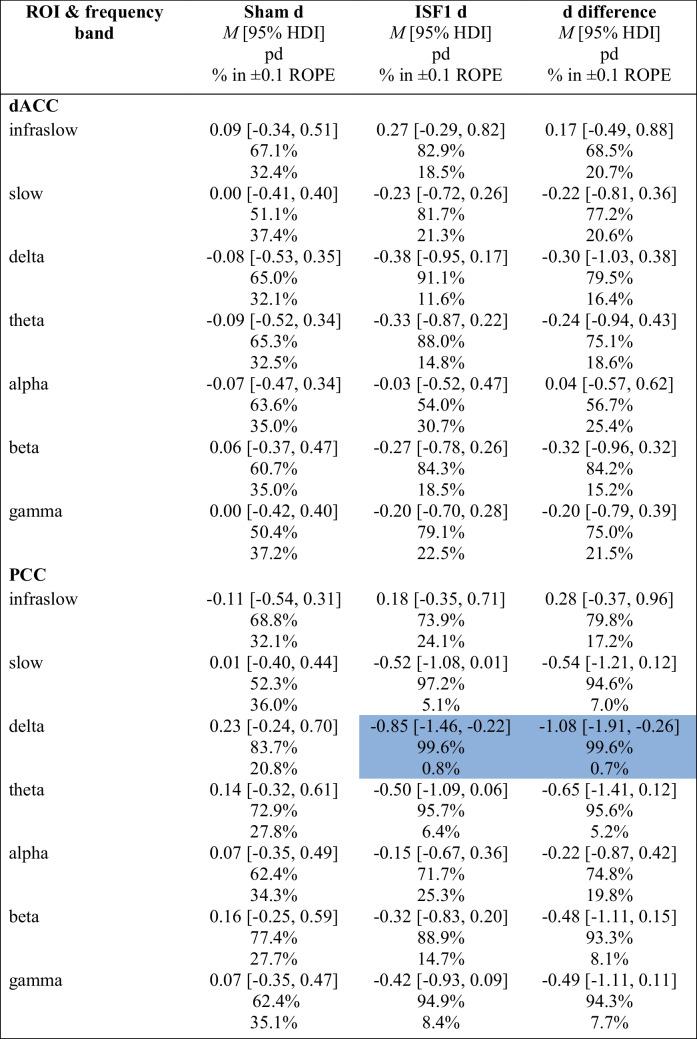
Activity (log-CSD) changes and change differences in each frequency band for sham and ISF1

Dark blue cell = significant reduction; ISF1 = 1-region infraslow neurofeedback*;* ROI = region of interest; dACC = dorsal anterior cingulate cortex; PCC = posterior cingulate cortex; *M* = mean; HDI = highest density interval; pd = probability of direction; % in ± 0.1 ROPE = percentage of the posterior in the ± 0.1 region of practical equivalence; d = Cohen’s d

**Table 6:** Activity (log-CSD) changes and change differences in each frequency band for sham and ISF2

ROI and frequency band	Sham d *M* [95% HDI] pd % in ± 0.1 ROPE	ISF2 d *M* [95% HDI] pd % in ± 0.1 ROPE	d difference *M* [95% HDI] pd % in ± 0.1 ROPE
**dACC**			
Infraslow	0.09 [− 0.34, 0.51] 67.1% 32.4%	0.12 [− 0.40, 0.66] 67.7% 26.2%	0.03 [− 0.60, 0.68] 53.5% 24.2%
Slow	0.00 [− 0.41, 0.40] 51.1% 37.4%	− 0.05 [− 0.54, 0.46] 57.2% 30.2%	− 0.04 [− 0.63, 0.54] 55.1% 26.4%
Delta	− 0.08 [− 0.53, 0.35] 65.0% 32.1%	− 0.25 [− 0.81, 0.31] 81.0% 19.1%	− 0.16 [− 0.87, 0.53] 67.4% 20.4%
Theta	− 0.09 [− 0.52, 0.34] 65.3% 32.5%	− 0.34 [− 0.90, 0.22] 88.7% 13.9%	− 0.26 [− 0.96, 0.42] 76.5% 17.9%
Alpha	− 0.07 [− 0.47, 0.34] 63.6% 35.0%	− 0.44 [− 0.95, 0.06] 95.4% 7.8%	− 0.36 [− 0.96, 0.23] 88.9% 13.0%
Beta	0.06 [− 0.37, 0.47] 60.7% 35.0%	− 0.45 [− 1.00, 0.09] 95.0% 8.0%	− 0.51 [− 1.17, 0.15] 93.6% 7.6%
Gamma	0.00 [− 0.42, 0.40] 50.4% 37.2%	− 0.30 [− 0.83, 0.22] 87.3% 16.0%	− 0.30 [− 0.92, 0.32] 83.2% 16.3%
**PCC**			
Infraslow	− 0.11 [− 0.54, 0.31] 68.8% 32.1%	− 0.18 [− 0.72, 0.35] 74.6% 23.5%	− 0.07 [− 0.71, 0.57] 58.9% 24.0%
Slow	0.01 [− 0.40, 0.44] 52.3% 36.0%	− 0.11 [− 0.64, 0.41] 66.5% 27.1%	− 0.13 [− 0.74, 0.52] 65.1% 23.2%
Delta	0.23 [− 0.24, 0.70] 83.7% 20.8%	− 0.14 [− 0.72, 0.44] 67.9% 24.2%	− 0.37 [− 1.13, 0.37] 83.4% 13.3%
Theta	0.14 [− 0.32, 0.61] 72.9% 27.8%	− 0.17 [− 0.75, 0.41] 72.2% 22.5%	− 0.32 [− 1.07, 0.43] 79.6% 15.1%
Alpha	0.07 [− 0.35, 0.49] 62.4% 34.3%	− 0.09 [− 0.62, 0.43] 63.4% 27.8%	− 0.16 [− 0.80, 0.47] 68.8% 21.8%
Beta	0.16 [− 0.25, 0.59] 77.4% 27.7%	− 0.16 [− 0.69, 0.38] 71.8% 24.6%	− 0.32 [− 0.98, 0.33] 83.5% 15.4%
Gamma	0.07 [− 0.35, 0.47] 62.4% 35.1%	− 0.09 [− 0.62, 0.43] 63.5% 27.8%	− 0.16 [− 0.79, 0.47] 68.8% 22.1%

ISF2 = 2-region infraslow neurofeedback; ROI = region of interest; dACC = dorsal anterior cingulate cortex; PCC = posterior cingulate cortex; *M* = mean; HDI = highest-density interval; pd = probability of direction; % in ± 0.1 ROPE = percentage of the posterior in the ± 0.1 region of practical equivalence; d = Cohen’s d

Generally, there were no notable dACC and PCC log-CSD changes within or change differences between groups. A notable exception is the PCC’s delta band (2–3.5 Hz). Specifically, ISF1 changes probably exist (99.6% pd) with a large effect size (− 0.85 d [95% HDI, − 1.46 to − 0.22]) that is certainly significant (0.8% in ROPE). Likewise, a change difference between ISF1 and sham probably exists (99.6% pd) with a large effect size (− 1.08 d [95% HDI, − 1.91 to − 0.26) that is certainly significant (0.7% in ROPE).

#### Functional connectivity

To assess change and change differences in the functional connectivity (FC) between the targeted ROIs (i.e., dACC & PCC) from baseline to post 6 sessions, a Bayesian model was used to estimate posterior distributions for the infraslow (0.01–0.1 Hz), slow (0.2–1.5 Hz), delta (2–3.5 Hz), theta (4–7.5 Hz), alpha (8–12 Hz), beta (12.5–30 Hz), and gamma (30.5–44 Hz) frequency bands. Tables 7 and 8 show summary statistics and include the group-wise posterior mean Cohen’s d of the FC changes (d) with 95% HDI, the posterior mean effects size (i.e., Cohen’s d) of the difference in FC changes (d difference) with 95% HDI, probability of direction (pd) of the d and d difference, and the percentage in ± 0.1 region of practical equivalence (% in ± 0.1 ROPE) of the full d and d difference posterior distributions.

**Table 7:** Functional connectivity (FC) changes and change differences for sham and ISF1

ROI-ROI and frequency band	Sham d *M* [95% HDI] pd % in ± 0.1 ROPE	ISF1 d *M* [95% HDI] pd % in ± 0.1 ROPE	d difference *M* [95% HDI] pd % in ± 0.1 ROPE
**dACC-PCC**			
Infraslow	0.33 [− 0.06, 0.74] 94.9% 11.1%	0.19 [− 0.29, 0.68] 78.1% 24.1%	− 0.14 [− 0.71, 0.42] 69.4% 24.2%
Slow	0.00 [− 0.41, 0.41] 50.8% 38.1%	− 0.06 [− 0.55, 0.42] 59.6% 31.7%	− 0.06 [− 0.60, 0.47] 59.1% 29.1%
Delta	0.00 [− 0.40, 0.43] 51.3% 38.0%	0.05 [− 0.43, 0.56] 59.1% 30.9%	0.05 [− 0.48, 0.59] 57.5% 29.4%
Theta	− 0.04 [− 0.44, 0.35] 57.7% 37.3%	0.21 [− 0.27, 0.68] 80.7% 22.3%	0.25 [− 0.30, 0.81] 81.3% 22.3%
Alpha	− 0.16 [− 0.54, 0.24] 78.8% 28.9%	− 0.07 [− 0.55, 0.40] 61.1% 31.3%	0.09 [− 0.46, 0.63] 63.3% 26.7%
Beta	0.02 [− 0.36, 0.41] 54.4% 39.2%	− 0.02 [− 0.47, 0.44] 53.6% 33.2%	− 0.04 [− 0.58, 0.46] 56.2% 29.9%
Gamma	− 0.01 [− 0.40, 0.37] 52.5% 38.7%	− 0.02 [− 0.49, 0.43] 53.7% 33.0%	− 0.01 [− 0.53, 0.52] 51.5% 29.6%

ISF1 = 1-region infraslow neurofeedback; ROI-ROI = functionally connected regions of interest; dACC = dorsal anterior cingulate cortex; PCC = posterior cingulate cortex; *M* = mean; HDI = highest-density interval; pd = probability of direction; % in ± 0.1 ROPE = percentage of the posterior in the ± 0.1 region of practical equivalence; d = Cohen’s d

**Table 8:**
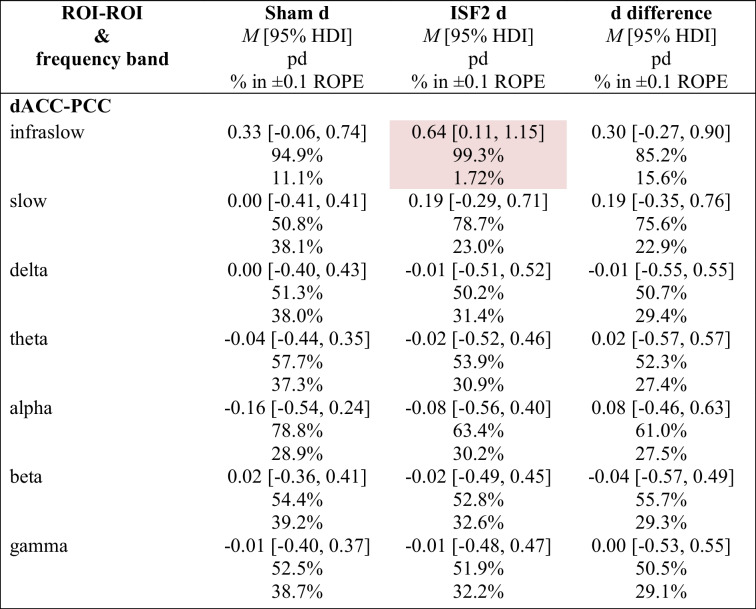
Functional connectivity (FC) changes and change differences for sham and ISF2

Pink cell = probably significant elevation; ISF2 = 2-region infraslow neurofeedback; ROI-ROI = functionally connected regions of interest; dACC = dorsal anterior cingulate cortex; PCC = posterior cingulate cortex; *M* = mean; HDI = highest-density interval; pd = probability of direction; % in ± 0.1 ROPE = percentage of the posterior in the ± 0.1 region of practical equivalence; d = Cohen’s d

Generally, there were no notable dACC-PCC changes within or change differences between groups. That said, ISF2 showed changes in the infraslow (0.01–0.1 Hz) band that probably exist (99.3% pd) with a point estimate suggesting a medium effect size (0.64 d [95% HDI, 0.11–1.15]) of probable significance (1.72% in ROPE).

#### Regression: FC vs. HADS

We implemented a Bayesian regression model to assess for associations between changes in the primary PRO subscales (i.e., HADS-A & HADS-D) and targeted ROI FC from baseline to post 6 sessions in the infraslow (0.01–0.1 Hz), slow (0.2–1.5 Hz), delta (2–3.5 Hz), theta (4–7.5 Hz), alpha (8–12 Hz), beta (12.5–30 Hz), and gamma (30.5–44 Hz) frequency bands. Table 9 shows summary statistics for each primary subscale and includes the posterior of the mean standardised regression coefficient (sβ) with 95% HDI, pd of the sβ, and % in ± 0.05 ROPE of the sβ. There were no notable associations between HADS-A or HADS-D change scores and dACC-PCC FC changes.

**Table 9:** Associations between Hospital Anxiety & Depression Scale (HADS) subscale score changes and functional connectivity (FC) changes

ROI-ROI and frequency band	HADS-A sβ *M* [95% HDI] pd % in ± 0.05 ROPE	HADS-D sβ *M* [95% HDI] pd % in ± 0.05 ROPE
**dACC-PCC**		
Infraslow	0.29 [− 0.02, 0.59] 96.7% 4.7%	− 0.06 [− 0.37, 0.26] 64.3% 23.0%
Slow	− 0.18 [− 0.49, 0.14] 86.9% 13.2%	− 0.18 [− 0.50, 0.13] 87.4% 13.2%
Delta	0.13 [− 0.19, 0.44] 79.2% 17.7%	0.02 [− 0.29, 0.34] 56.2% 24.4%
Theta	0.17 [− 0.14, 0.49] 85.9% 14.1%	− 0.17 [− 0.48, 0.14] 85.4% 14.4%
Alpha	0.18 [− 0.14, 0.48] 87.1% 13.2%	0.07 [− 0.25, 0.38] 66.6% 22.7%
Beta	− 0.14 [− 0.45, 0.18] 81.1% 16.8%	− 0.01 [− 0.33, 0.31] 52.6% 24.7%
Gamma	0.01 [− 0.31, 0.33] 52.2% 24.6%	0.09 [− 0.23, 0.40] 70.9% 21.2%

ROI-ROI = functionally connected regions of interest; HADS-A = anxiety subscale; HADS-D = depression subscale; *M* = mean; HDI = highest-density interval; pd = probability of direction; % in ± 0.05 ROPE = percentage of the posterior in the ± 0.05 region of practical equivalence; sβ = standardised beta

#### Regression: ROI activity vs. HADS

We implemented a Bayesian regression model to assess for associations between changes in the primary PRO subscales (i.e., HADS-A & HADS-D) and targeted ROI activity from baseline post-6 sessions (post 6) in the infraslow (0.01–0.1 Hz), slow (0.2–1.5 Hz), delta (2–3.5 Hz), theta (4–7.5 Hz), alpha (8–12 Hz), beta (12.5–30 Hz), and gamma (30.5–44 Hz) frequency bands. Table 10 shows summary statistics for each primary subscale and includes the posterior of the mean standardised regression coefficient (sβ) with 95% HDI, pd of the sβ, and % in ± 0.05 ROPE of the sβ.

**Table 10:**
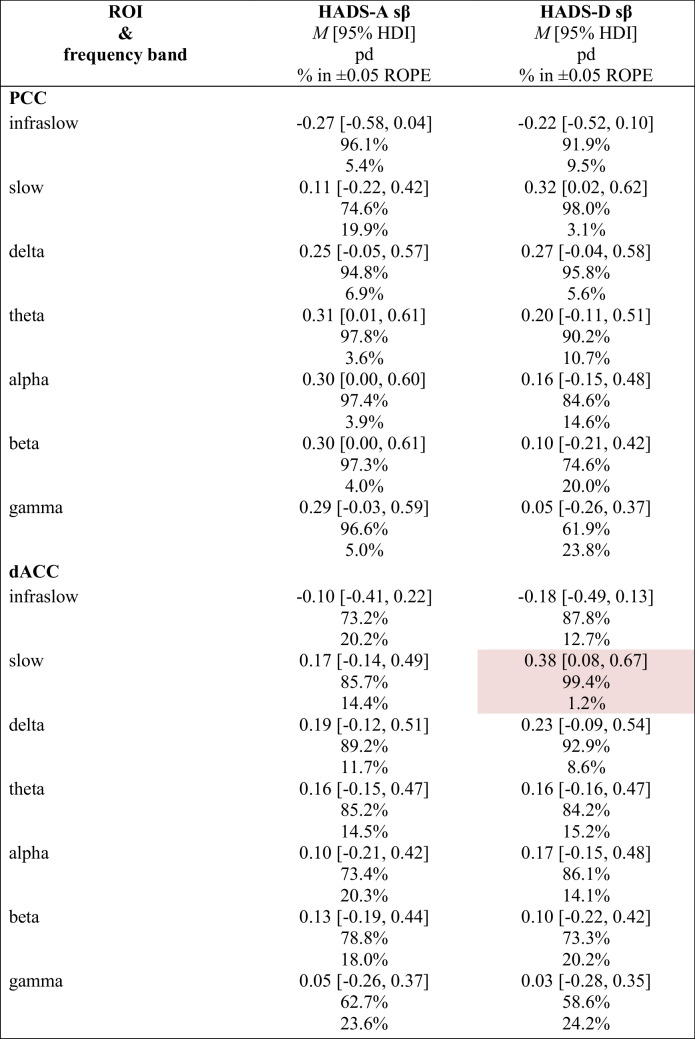
Associations between HADS subscale score changes and targeted ROI activity (log-CSD) changes

Pink cell = probably significant positive association; ROI = region of interest; HADS-A = anxiety subscale; HADS-D = depression subscale; *M* = mean; HDI = highest-density interval; pd = probability of direction; % in ± 0.05 ROPE = percentage of the posterior in the ± 0.05 region of practical equivalence; sβ = standardised beta

**Fig. 9:**
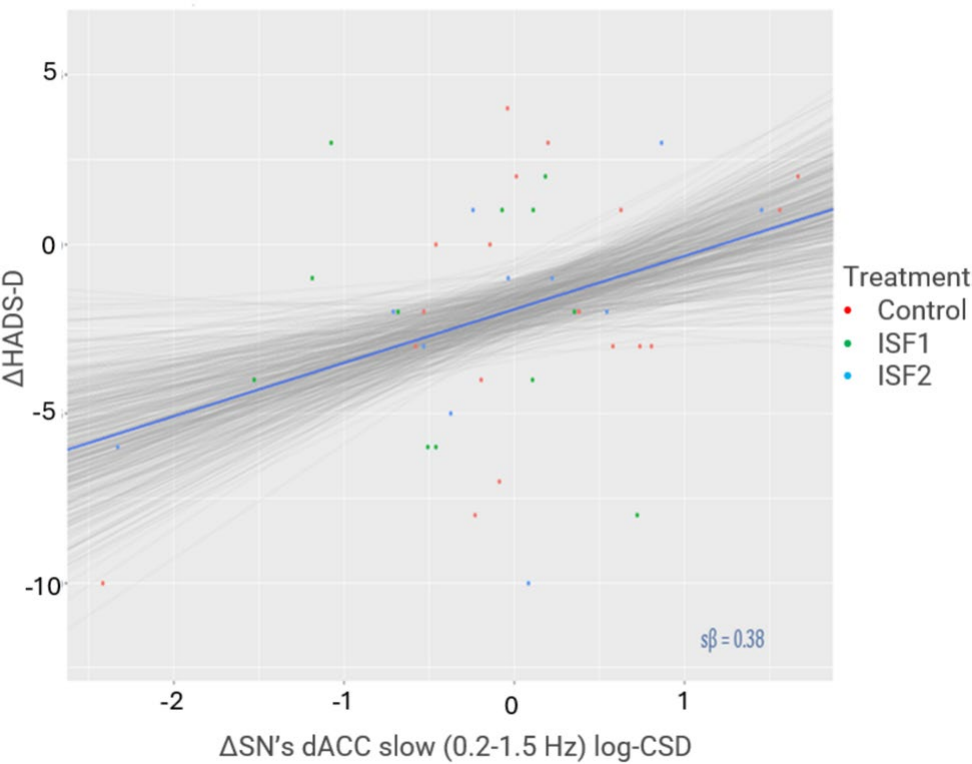
Association between Hospital Anxiety & Depression Scale – Depression Subscale (HADS-D) Change Scores & SN’s dACC Slow Band Activity (log-CSD) Changes. *Note.* Δ = change; SN = Salience Network; dACC = dorsal anterior cingulate cortex; ISF1 = 1-region infraslow neurofeedback; ISF2 = 2-region infraslow neurofeedback; Control = sham; Grey lines = 500 random regression lines from the posterior distribution; Dark blue line = mean regression line; sβ = standardised beta

Generally, there were no notable associations between HADS-A or HADS-D change scores and ROI activity. A notable exception is the association between HADS-D change scores and dACC slow (0.2–1.5 Hz) activity changes which probably exists (99.4% pd) with a point estimate suggesting a *positive*, moderate association strength (0.38 sβ [95% HDI, 0.08–0.67]) that is probably significant (1.2% in ROPE). A regression plot of the HADS-D change scores as a function of SN’s dACC slow band activity changes is provided in Fig. 9.

#### Regression: Placebo Network activity vs. HADS (Exploratory)

As part of an exploratory analysis, we implemented a Bayesian regression model to assess for associations between changes in the primary PRO subscales (i.e., HADS-A & HADS-D) and the activity of the “placebo network” (PN) ROIs from baseline to post 6 sessions in the infraslow (0.01–0.1 Hz), slow (0.2–1.5 Hz), delta (2–3.5 Hz), theta (4–7.5 Hz), alpha (8–12 Hz), beta (12.5–30 Hz), and gamma (30.5–44 Hz) frequency bands. Table 11 shows summary statistics for each primary subscale and includes the posterior of the mean standardised regression coefficient (sβ) with 95% HDI, pd of the sβ, and % in ± 0.05 ROPE of the sβ.

**Table 11:**
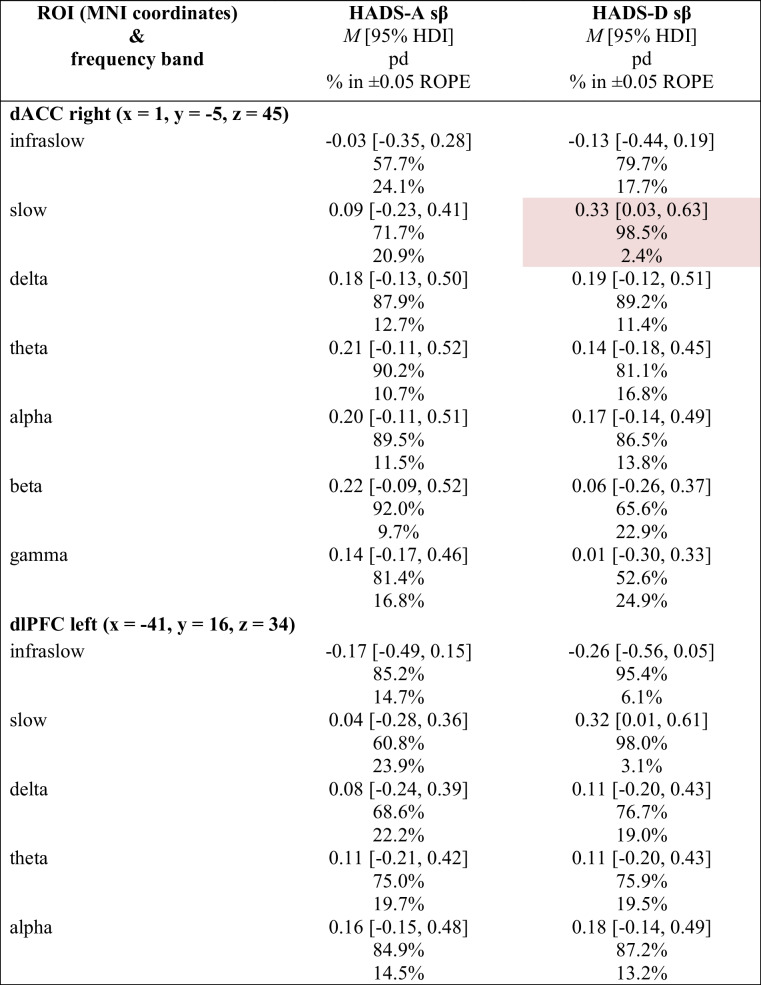

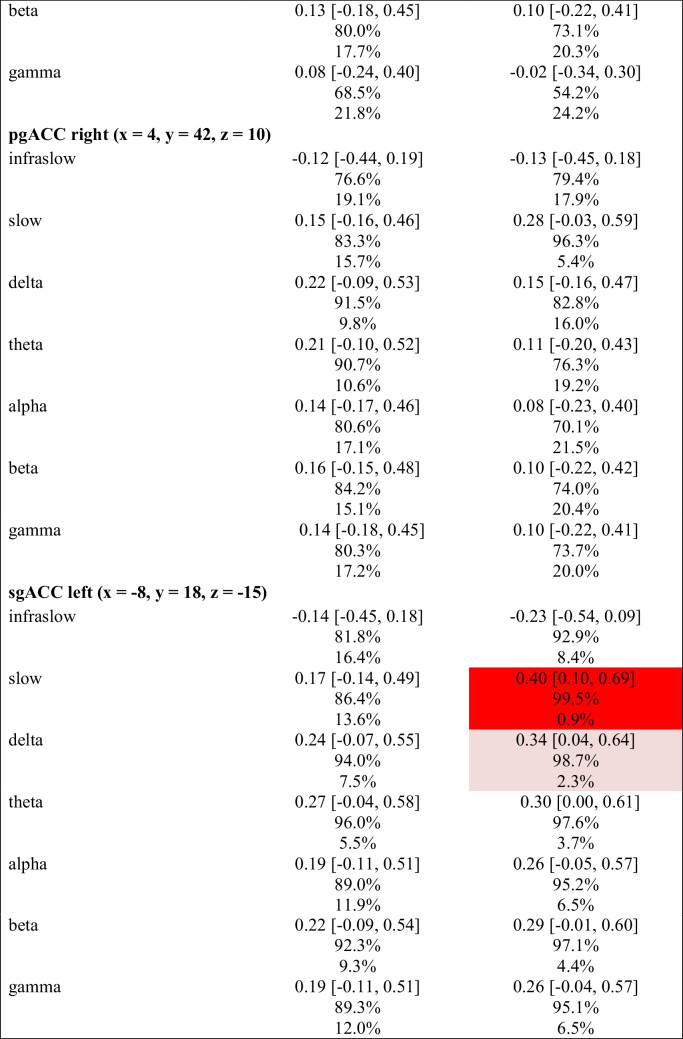
Associations between Hospital Anxiety & Depression Scale (HADS) subscale score changes and placebo network (PN) activity (log-CSD) changes

Pink cell = probably significant positive association; Red cell = certainly significant positive association; ROI = region of interest; HADS-A = anxiety subscale; HADS-D = depression subscale; dACC = dorsal anterior cingulate cortex; dlPFC = dorsolateral prefrontal cortex; pgACC = pregenual anterior cingulate cortex; sgACC = subgenual anterior cingulate cortex; MNI = Montreal Neurological Institute; *M* = mean; HDI = highest-density interval; pd = probability of direction; % in ± 0.05 ROPE = percentage of the posterior in the ± 0.05 region of practical equivalence; sβ = standardised beta

**Fig. 10:**
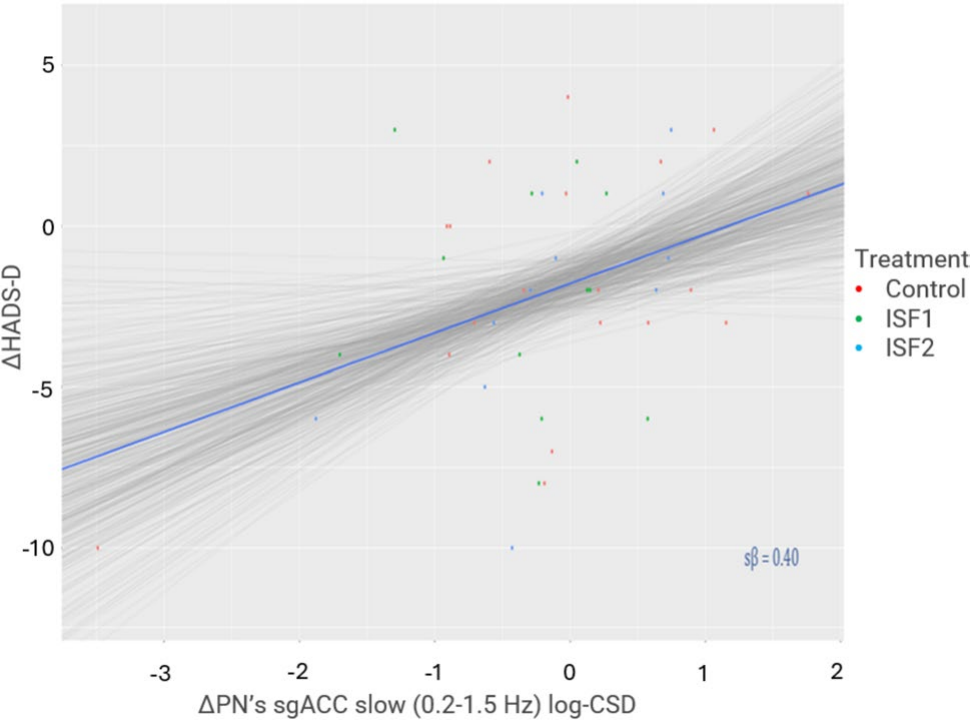
Association between Hospital Anxiety & Depression Scale – Depression Subscale (HADS-D) Change Scores & PN’s sgACC Slow Band Activity (log-CSD) changes. *Note.* Δ = change. PN = placebo network; sgACC = subgenual anterior cingulate cortex; ISF1 = 1-region infraslow neurofeedback; ISF2 = 2-region infraslow neurofeedback; Control = sham; Grey lines = 500 random regression lines from the posterior distribution; Dark blue line = mean regression line; sβ = standardised beta

Generally, there were no notable associations between HADS-A or HADS-D change scores and PN activity. Notable exceptions include 1) the association between HADS-D change scores and the PN’s dACC slow (0.2–1.5 Hz) activity, which likely exists (98.5% pd) with a point estimate suggesting a *positive*, moderate association strength (0.33 sβ [95% HDI, 0.03–0.63]) of probable significance (2.4% in ROPE), and 2) the associations between changes in HADS-D and the PN’s sgACC slow (0.2–1.5 Hz) and delta (2–3.5 Hz) activity, which likely (slow: 99.5% pd) and probably (delta: 98.7% in pd) exist with point estimates suggesting *positive*, moderate association strengths (slow: 0.40 sβ [95% HDI, 0.10–0.69]; delta: 0.34 sβ [95% HDI, 0.04–0.64]) of certain (slow: 0.9% in ROPE) and probable (delta: 2.3% in ROPE) significance. A regression plot of the HADS-D change scores as a function of the PN’s sgACC slow activity change is provided in Fig. 10.

#### Secondary endpoints, side-effects, Bayesian model diagnostics

See Supplement for results from secondary endpoints, DESS, and Bayesian model diagnostics.

## Discussion

In partial confirmation of our initial hypothesis, we found credible evidence of strong *nonspecific* clinical effects as evidenced by significant improvements (i.e., reductions) in HADS scores irrespective of group assignment. Indeed, negative standardised effect size point estimates exceeding the standardised MCID values were evident in at least one subscale for all groups. That said, we found no credible evidence for *specific* clinical effects as indicated by an absence of HADS score change differences (i.e., differences in standardised effect sizes) between the sham and active groups.

Regarding EEG-based metrics, although absent in sham and ISF2, there is credible evidence to support targeted EEG learning in the ISF group as indicated by a reduction in delta activity within the targeted *ROI* (i.e., PCC). Furthermore, although lacking in the ISF2 vs. sham contrast, whole-brain and ROI-specific analyses shows credible evidence to support a greater reduction in the activity in the slow and/or delta bands within the PCC of ISF1 compared with sham. That said, activity changes and change differences in the targeted *band* (i.e., ISFs = 0.01–0.1 Hz) were noticeably absent within and between groups. With respect to FC, although lacking in both sham and ISF groups, credible changes in dACC-PCC FC in the targeted infraslow band were found for ISF2. However, change differences were absent across all contrasts (i.e., sham vs. ISF1 and sham vs. ISF2) and frequency bands (i.e., infraslow, slow, delta, theta, alpha, beta, gamma). Finally, apart from a *positive* moderate association between HADS-D change scores and slow band activity modulation within the SN’s dACC, credible associations between changes in HADS subscale scores and EEG metrics were generally lacking. Intriguingly, exploratory analyses revealed credible *positive* moderate associations between HADS-D change scores and delta and/or slow band activity modulation within key PN nodes (i.e., sgACC, dACC).

Placebo-related effect sizes are known to be large in ID-related clinical trials (Ashar et al., 2017; Huneke et al., 2020; Jones et al., 2021), and the evidence suggests that they may have played a dominant role in the current trial. To elaborate, placebo effects can be defined as beneficial therapeutic effects derived from complex brain responses to the context of the treatment rather than the treatment itself and are mediated by a myriad of external (e.g., exposure to novel technology) and internal (e.g., Bayesian predictions) factors (Wager & Atlas, 2015). The magnitude of the placebo response across treatment modalities used for treatment-resistant depression in adults is large (g = 1.05; 95% confidence interval 0.91–1.1) (Jones et al., 2021), which makes it extremely hard for studies of any treatment modality in this population to demonstrate specific effects superior to placebo. A recent meta-analysis has elucidated a link between placebo effects and activity modulations within a collection of nodes (i.e., right dACC, right pgACC, left sgACC/ventral striatum, left dlPFC, and right basal ganglia) aptly named the “placebo network” (PN) (Burke et al., 2021), which appears to overlap considerably with the triple-network (Menon, 2011). These findings have been echoed in another recent review looking specifically at the neural correlates of ID-related placebo responses (Huneke et al., 2022). Importantly, the credible associations between HADS-D change scores and PN-related *activity* changes in our pilot trial might suggest that the substantial clinical responses may have been driven, at least in part, by placebo effects.

Notably, among the strongest and most credible associations with HADS-D change scores are changes in slow band activity within ACC nodes (i.e., sgACC, dACC). Interestingly, these nodes were among a select few identified in a recent systematic review as putative neural correlates of dysfunctional automatic emotion regulation in MDD (Rive et al., 2013); however, the indictment of slow oscillations, which are commonly filtered out during EEG pre-processing (Shim et al., 2018; Wu et al., 2021; Yan et al., 2021) or ignored (Sook Ling Leong et al., 2018a, 2018b), appears novel. Slow oscillations (SOs; ~  < 1 Hz) were first described in anesthetized animals (Steriade et al., 1993; Wilson & Groves, 1981) and are generally defined as alternating periods of synchronous depolarisations (“up” states) and hyperpolarisations (i.e., “down” states) of cortical pyramidal neurons, which are augmented during periods of reduced sensory input from the external environment (Neske, 2015). Commonly associated with various functions during sleep (e.g., memory consolidation, cellular repair) (Neske, 2015), the behavioural relevance of SOs in the waking human brain is, to our knowledge, largely unknown. Based the results of this trial, we tentatively propose that SOs may function in the regulation of mood during the waking state. What is mood? Within the framework of the Bayesian brain theory, mood is a state of mind that functions as a hyperprior that influences uncertainty and emotional states (Clark et al., 2018). As such, a low (i.e., depressed) mood might be conceptualised as a state whereby “the brain is certain that it will encounter an uncertain environment,” resulting in ongoing negatively valenced emotions (Clark et al., 2018). Consequently, a depressed mood will result in withdrawal from the predicted uncertain environment. Considering that SOs function on an extended temporal scale, they are well placed to regulate slower processes, such as mood. In keeping with this Bayesian perspective, it could be hypothesised that long-term average mood constrains short-term emotional fluctuations neurobiologically via a recently discovered form of FC known as cross-frequency coupling (CFC), whereby lower frequency oscillations (e.g., SOs) modulate higher-frequency oscillations (e.g., gamma) (Canolty & Knight, 2010; Neske, 2015; Schutter & Knyazev, 2012); the latter is widely recognised to be associated with prediction errors (Arnal et al., 2011; Hein & Herrojo Ruiz, 2022; Strube et al., 2021), which may lead to negative emotional processing (Gemignani et al., 2000; Luo et al., 2007; Matsumoto et al., 2006; Müller et al., 1999; Oathes et al., 2008; Oya et al., 2002; Sebastiani et al., 2003). Interestingly, associations between ID-related traits/symptoms and CFC of traditional frequency bands (i.e., delta, alpha, beta) within key nodes of the triple-network (e.g., dACC) have recently been described (Knyazev et al., 2019). Moreover, thalamocortical dysrhythmia, a CFC phenomenon, whereby abnormal low frequency rhythms in the thalamus drive dysfunctional activity in and interactions between low- and high-frequency rhythms at the cortex, is theorised to be a central mechanism responsible for neuropsychiatric symptomatology (Llinás et al., 1999, 2005; Schulman et al., 2011). An interestingly line of research would be to investigate potential ISF-NFB induced CFC modulations along with associations between CFC modulations and clinical symptom changes using novel resting-state CFC analyses. Although nascent, we and others (Sacks et al., 2021) view CFC as a very promising area of research in the neuropsychiatric space.

Given that strong placebo effects are likely at play, this still fails to explain the apparent lack of specific effects in the active groups. There are several potential explanations for this outcome. One possibility is that clinical outcome improvements are primarily driven by nonspecific effects as posited by some NFB sceptics (Arnold et al., 2020; Ghaziri & Thibault, 2019; Schönenberg et al., 2017a, 2017b; Thibault et al., 2016, 2018a, 2018b). Indeed, our trial results are in line with those found in our recently published systematic review wherein all three included ID-related NFB trials showed small mean effect size *differences* between active and sham groups on subjective outcome measures (e.g., PROs) with 95% confidence intervals that spanned the null (Perez et al., 2022c). A second possibility is that, although we found some credible evidence of *differential* EEG learning within the targeted ROI (i.e., greater reduction in delta/slow band activity in ISF1 vs. sham), this learning did not appear to involve the targeted frequency band (i.e., ISFs), which precluded the possibility of ISF-mediated specific effects. If ISF modulation is a prerequisite for the induction of specific clinical effects from ISF-NFB, more active sessions may be required to induce significant ISF changes in ID populations. Furthermore, it is plausible that our use of a standardised auditory feedback signal may have been more informational than motivational and, therefore, not sufficiently reinforcing, on average, to induce significant ISF changes. In support, a recent NFB trial showed that differential EEG *activity* learning in the targeted frequency band was only apparent when participants were allowed to select the feedback signal that they found highly enjoyable (Pérez-Elvira et al., 2021). Future sham-controlled ISF-NFB trials could consider allowing participants to choose the feedback that they find most pleasing to enhance the motivational value of the reinforcement signal, thereby potentially fostering differential ISF changes in the targeted ROIs to ascertain whether specific effects are indeed induced. Another explanation may be related to the fact that most of the dropouts occurred within the sham allocation. If the true impetus for trial discontinuation was a lack of improvement from sham therapy, not having outcome data on these participants may have biased the sham group’s outcomes. That said, the proportion of dropouts in this group was relatively small and unlikely to have a significant impact on the outcomes.

Of note, putative ISF-NFB–related side-effects, although common, were generally very mild (see Supplement). This is consistent with previous studies that investigated the side-effects from NFB (Rogel et al., 2015). Moreover, like our previous ISF-NFB study (Leong et al., 2018a, 2018b), increased dreaming was the most salient side-effect experienced during this trial. Although informing participants of the possibility of this specific side-effect during the consent process may have played a role (Colagiuri et al., 2012), increased NFB-related postsession dreaming has been theorised to indicate the consolidation of procedural, nondeclarative learning (Yonah, 2020).

## Strengths and limitations

Our trial encompasses many key strengths. First, our relatively novel transdiagnostic approach heeds recent calls for a more pragmatic, ecologically valid clinical research (Bui & Fava, 2017; Cummings et al., 2014; Dickinson, 2017; Ehring & Watkins, 2008; Epkins & Heckler, 2011; Goldstein-Piekarski et al., 2016; Lahey et al., 2017; McTeague et al., 2016; Scholten et al., 2016; Wenzel & Jager-Hyman, 2014; Zald & Lahey, 2017). Second, unlike nearly all contemporary EEG-based trials (Vanhatalo et al., 2005), we were able to explore changes and change differences beyond traditional frequency bands owing to the utilization of broad-band EEG acquisitions. Third, our incorporation of modern source localization algorithms (i.e., eLORETA) combined with whole-head, high-density EEGs putatively allowed for reasonably accurate source estimations (Song et al., 2015). There are some limitations that are important to point out. First, we utilized generic head models based on template magnetic resonance images (MRIs) along with template electrode positions. Failure to incorporate subject-specific head models and electrode digitization has been shown to increase source localization errors (Liu et al., 2023). Second, although the existence of transdiagnostic biomarkers is well established (Menon, 2019, 2020), there exists substantial heterogeneity in individual neurobiological aberrations both between and within IDs, which may explain differential responsiveness to certain neuromodulatory therapies (Drysdale et al., 2017; Qi et al., 2023; Yan et al., 2023). As such, attempting to modulate neurobiological substrates without predetermining substrate dysfunction, which was done in this trial, has the potential to result in failed outcomes and incorrect conclusions. Future studies may consider more stringent eligibility criteria whereby given biomarkers must be present in an individual prior to enrolment. Second, because of the pilot nature of our trial, our sample size was relatively small constraining the precision of our estimates. Informed by our pilot data, future studies of similar design should consider incorporating larger sample sizes to constrain the width of confidence intervals within the desired range. Third, our study was comprised entirely of female participants; therefore, it would be premature to generalize our findings to male populations. Notably, we chose to limit recruitment to females owing to the significantly increased impact of IDs in this subgroup (Asher et al., 2017; Kessler et al., 2015; Stein et al., 2017). Fourth, our sample included only adult subjects. Because adolescence is well recognised as a critical period in the development of neuropsychiatric disorders (Kessler et al., 2005), it was not unexpected that nearly all our participants reported first-onset of internalizing symptoms in their adolescent years. Future ISF-NFB trials are advised to incorporate adolescent participants to investigate whether and how outcomes may differ in younger cohorts.

## Conclusions

This pilot trial showed that our short-term sham and genuine ISF-NFB interventions directed at an ID population resulted in rapid, clinically important improvements in self-reported symptoms (i.e., anxiety and/or depression) that were predominantly nonspecific in nature and possibly driven by placebo-related mechanisms. Notably, differential targeted EEG learning was generally absent as evidenced by a lack of ISF-related change differences between groups. It remains to be seen if future trial designs can implement design modifications that are capable of differential modulation of ISFs between sham and treatment groups to increase the potential for specific clinical effects and establish genuine ISF-NFB therapy as a valuable tool in the treatment of IDs and other psychopathologies.

## Supplementary Information

Below is the link to the electronic supplementary material.

Supplementary file1 (DOCX 12750 KB)

## Data Availability

In line with the scientific imperatives of open and reproducible science, de-identified datasets and statistical codes have been made openly available in GitHub at https://github.com/tysonperez/PhD_rct.git.

## Declarations

### Open practices statement

The data are available in GitHub (https://github.com/tysonperez/PhD_rct.git). The trial was prospectively registered with the Australia New Zealand Clinical Trials Registry (ANZCTR; Trial ID: ACTRN12619001428156), and the full trial protocol has been published previously (Perez et al., 2022a, 2022b, 2022c).
