# Supplementary material for: Infraslow Closed-Loop Brain Training for Anxiety and Depression (ISAD): A pilot randomised, sham-controlled trial in adult females with internalizing disorders
Source: Cogn Affect Behav Neurosci. 2025 Mar 18;25(4):1147–80. doi: 10.3758/s13415-025-01279-z (PMC12356751; doi:10.3758/s13415-025-01279-z)

Supplementary Material

**Table S1**

Template for Intervention Description and Replication (TIDieR)

| **Item number** | **Item** | **Description** |
| --- | --- | --- |
|  | **BRIEF NAME** Provide the name or a phrase that describes the intervention. | Infraslow neurofeedback for Anxiety & Depressive Disorders (ISADD) |
|  | **WHY** Describe any rationale, theory, or goal of the elements essential to the intervention. | Electrophysiological infraslow (<0.1 Hz) fluctuations (ISFs) are believed to coordinate and integrate information exchange within and between core resting-state networks (c-RSNs). Further, communication within and between c-RSNs has been found to be disrupted in internalizing disorder (ID) populations. We hypothesize that ISF neurofeedback (ISF-NFB) targeting key cortical nodes of these c-RSNs, may restore proper intra- and inter-network function and reduce ID-related symptoms. |
|  | **WHAT** Materials: Describe any physical or informational materials used in the intervention, including those provided to participants or used in intervention delivery or training of intervention providers. Provide information on where the materials can be accessed (e.g. online appendix, URL). | 19-channel sLORETA ISF-NFB training will be performed using a DC coupled amplifier (Brainmaster Technologies Inc.), ASUS laptop computer (ASUSTek Computer Inc.; 64.0 GB RAM; Intel Core i7 processor) running BrainAvatar software (version 4.7.5.844), 24 (Ag/AgCl) electrode Comby EEG caps, blunt needle, 5ml syringe, and electrolyte gel (Electro-Cap International Inc.). Free, open-source software (Audacity.com) is used to record/playback all auditory rewards during active/sham sessions. |
|  | **PROCEDURES**  Describe each of the procedures, activities, and/or processes used in the intervention, including any enabling or support activities. | Subjects were asked to arrive with non-braided, clean, dry hair. They were seated in a comfortable chair with their eyes closed in a quiet, cool (~15°C), dimly lit room. An appropriately sized Comby EEG cap was affixed to the head and, using a blunt need and syringe, the scalp was mildly abraded just prior to the application of the electrolyte gel beneath each electrode. EEGs were recorded with the Ag/AgCl electrodes positioned according to the International 10–20 system (i.e., Fp1, Fp2, F3, F4, C3, C4, P3, P4, O1, O2, F7, F8, T3, T4, T5, T6, Fz, Cz, Pz) using a linked mastoids reference and a ground electrode positioned centrally between, F3, Fp1, Fz and Fpz. Impedances were kept below 10 kΩ. Immediately prior to each training period, a demonstration of motion/EMG artefact alerts was provided with instructions to avoid eye/head/face movements to minimize this non-rewarding feedback. Participants were instructed to close their eyes, relax, stay awake, and listen to the sound being played. They were informed that the sound/music they will hear reflects that they are doing well. Continuous, real-time auditory feedback (organ tones) was used for reinforcement when the subject’s ISFs surpass the threshold(s). The reward threshold was manually adjusted in real-time to maintain a 60% ± 10% success rate. The yoked-sham sessions were identical to active sessions, including live EEG recordings and real-time motion/EMG artefact alerts, however the auditory rewards were derived from playbacks of consecutive, pre-recorded sessions of another female with IDs. The trainer remained present for the duration of all sessions to monitor the EEG. |
|  | **WHO PROVIDED** For each category of intervention provider (e.g., psychologist, nursing assistant), describe their expertise, background and any specific training given. | A non-blinded doctoral student with 2+ years of training and experience in the administration of NFB. |
|  | **HOW** Describe the modes of delivery (e.g., face-to-face or by some other mechanism, such as internet or telephone) of the intervention and whether it was provided individually or in a group. | sLORETA ISF-NFB sessions were performed one-on-one and face-to-face |
| 7. | **WHERE** Describe the type(s) of location(s) where the intervention occurred, including any necessary infrastructure or relevant features. | sLORETA ISF-NFB sessions took place in the EEG lab of the Department of Psychological Medicine, University of Otago, Dunedin, New Zealand. |
|  | **WHEN and HOW MUCH** Describe the number of times the intervention was delivered and over what period of time including the number of sessions, their schedule, and their duration, intensity or dose. | Participants attended three 30-minute sessions per week over 4 consecutive weeks (12 sessions in total). |
|  | **TAILORING** If the intervention was planned to be personalized, titrated or adapted, then describe what, why, when, and how. | Auditory feedback during active sLORETA ISF-NFB sessions will be based on each person’s real-time EEG-derived cortical ISFs. Thresholds will be manually adjusted, as needed, to maintain the pre-specified feedback success rate (i.e., 60% ± 10%) |
|  | **MODIFICATIONS** If the intervention was modified during the course of the study, describe the changes (what, why, when, and how). | The intervention was not modified during the study. |
|  | **HOW WELL** Planned: If intervention adherence or fidelity was assessed, describe how and by whom, and if any strategies were used to maintain or improve fidelity, describe them. | Protocol adherence was monitored by the trainer. Attempts were made to mitigate adherence issues via automated email and text message reminders sent on the day of each training session. |
|  | **ACTUAL** If intervention adherence or fidelity was assessed, describe the extent to which the intervention was delivered as planned. | The intervention was delivered as planned to all participants. |

**Table S2**

*Schedule of Trial Activities*

| **Trial Activities** | **T0** | **T1** | **T2** | **T3-8** | **T9** |
| --- | --- | --- | --- | --- | --- |
| **Enrolment** | | | | | |
| Informed consent | 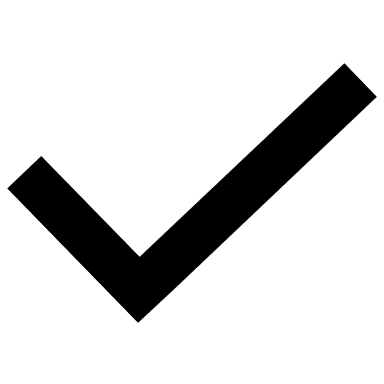 |  |  |  |  |
| Eligibility screen (MINI) | 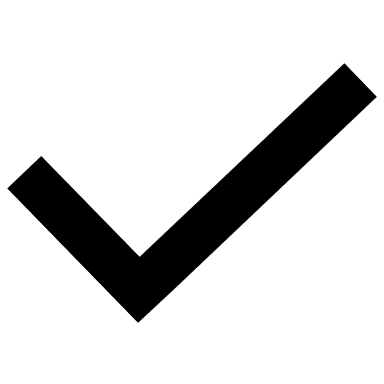 |  |  |  |  |
| Anthropometric measures | 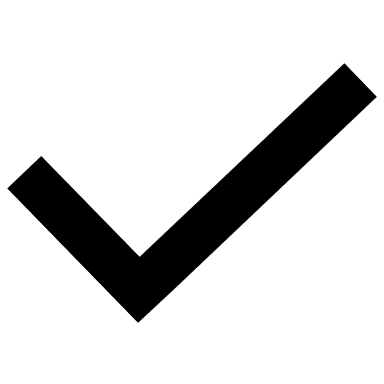 |  |  |  |  |
| **Outcome Assessments** |  |  |  |  |  |
| HADS, MEDI, IDAS-II |  | 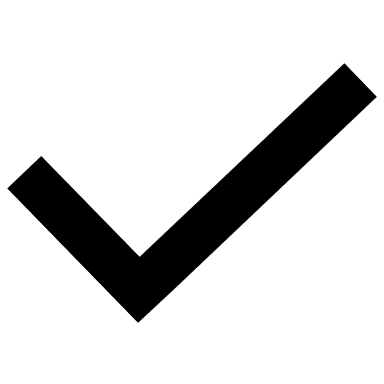 | 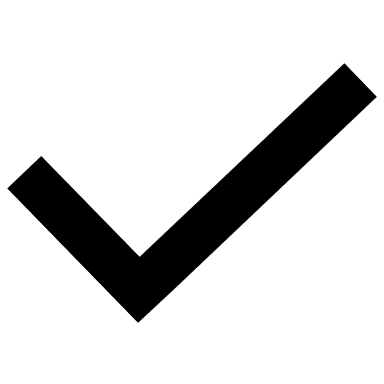 |  | 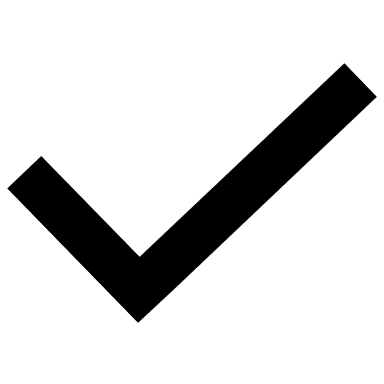 |
| EEG |  | 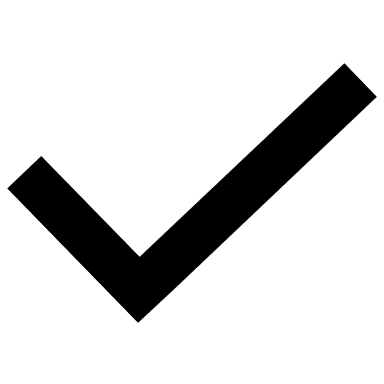 | 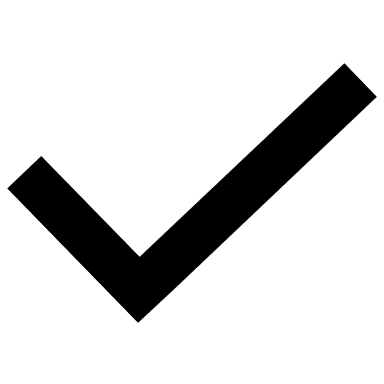 |  | 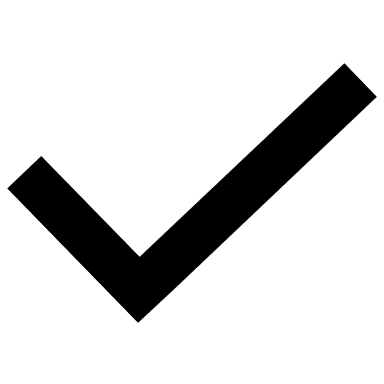 |
| ECG |  | 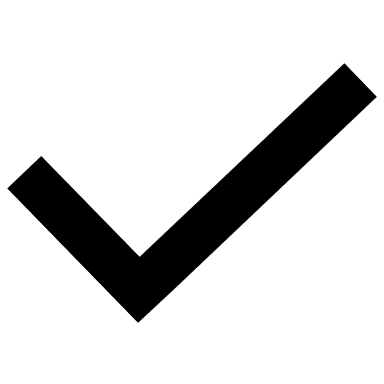 | 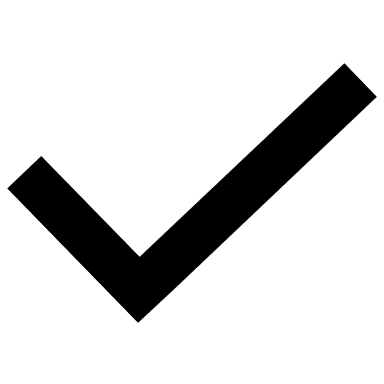 |  | 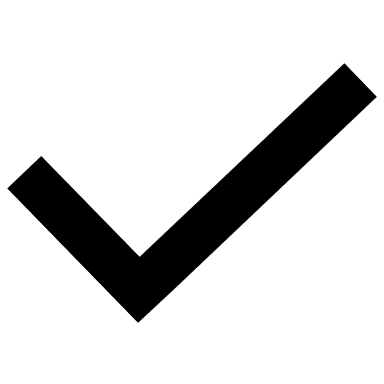 |
| **Interventions** | | | | | |
| Active ISF-NFB |  |  |  | 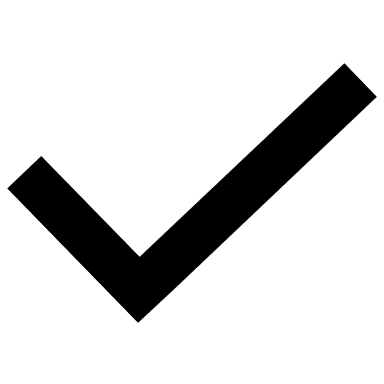 |  |
| Sham ISF-NFB |  |  |  | 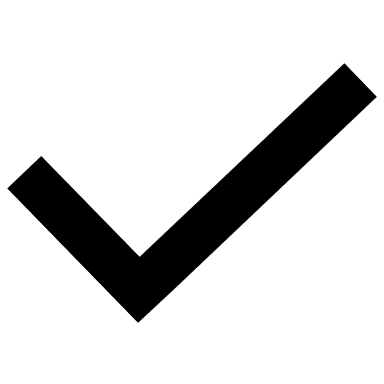 |  |
| **Safety Monitoring** |  |  |  |  |  |
| DESS |  |  |  | 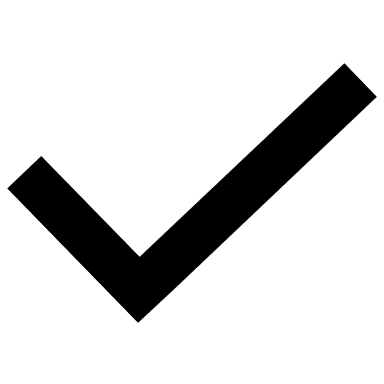 | 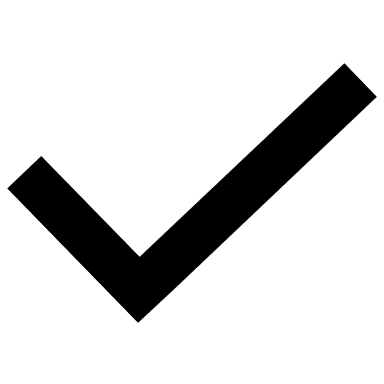 |

*Note*. T0 = initial interview. T1 = baseline #1. T2 = baseline #2. T3-8 = training sessions 1 through 6. T9 = post 6 session assessments.

**Table S3**

Baseline Clinical Characteristics of ID Participants by Group Assignment. for Secondary Outcomes of Interest

| **PROs & subscales** | **Sham**  *n*=30 | **ISF1**  *n*=15 | **ISF2**  *n*=15 |
| --- | --- | --- | --- |
| **MEDI*** | | | |
| Neurotic Temperament {0-40} | 26.3 (6.5) | 28.7 (7.6) | 25.7 (9.5) |
| Positive Temperament {0-40} | 21.7 (5.02) | 16.4 (6.6) | 17.7 (7.8) |
| Depression {0-40} | 20.2 (7.8) | 21.3 (7.9) | 21.5 (10.6) |
| Social Concerns {0-40} | 24.5 (10.5) | 26.1 (10.1) | 26.3 (12.8) |
| Autonomic Arousal {0-40} | 14.3 (9.3) | 16.8 (8.8) | 12.5 (11.9) |
| Somatic Anxiety {0-40} | 15.8 (10.3) | 18.0 (7.1) | 13.3 (10.6) |
| Intrusive Cognition {0-48} | 15.8 (8.9) | 19.7 (12.0) | 17.0 (11.2) |
| Traumatic Reexperiencing {0-40} | 11.3 (11.4) | 16.9 (12.3) | 12.1 (10.4) |
| Avoidance {0-64} | 26.1 (13.3) | 27.9 (12.5) | 28.6 (11.1) |
| **IDAS-II*** | | | |
| General Depression {20-100} | 57.5 (11.2) | 63.6 (14.4) | 64.9 (12.1) |
| Social Anxiety {6-30} | 14.7 (5.3) | 16.4 (5.3) | 17.6 (6.7) |
| Dysphoria {10-50} | 29.4 (7.5) | 32.1 (8.4) | 34.2 (7.1) |
| Lassitude {6-30} | 18.6 (4.4) | 19.7 (5.2) | 19.9 (4.3) |
| Insomnia {6-30} | 16.3 (5.7) | 15.9 (4.7) | 17.7 (5.6) |
| Appetite Loss {3-15} | 6.9 (3.1) | 7.5 (3.1) | 6.8 (4.1) |
| Appetite Gain {3-15} | 7.4 (3.1) | 7.8 (3.2) | 8.5 (3.2) |
| Well-Being {8-40} | 18.0 (5.6) | 15.3 (4.3) | 17.1 (6.7) |
| Ill Temper {5-25} | 9.4 (4.2) | 10.4 (5.8) | 10.2 (4.6) |
| Traumatic Intrusions {4-20} | 8.0 (3.5) | 8.9 (4.9) | 8.3 (4.2) |
| Traumatic Avoidance {4-20} | 9.5 (3.9) | 8.7 (4.7) | 9.5 (4.6) |
| Panic {8-40} | 14.7 (4.7) | 15.3 (5.5) | 15.3 (6.3) |
| Suicidality {6-30} | 8.3 (2.8) | 10.0 (5.0) | 9.9 (6.4) |
| Mania {5-25} | 10.7 (4.6) | 10.5 (5.5) | 9.9 (4.2) |
| Euphoria {5-25} | 6.2 (1.7) | 6.1 (1.6) | 6.9 (3.0) |
| Claustrophobia {5-25} | 6.8 (3.4) | 9.1 (4.8) | 7.7 (4.3) |
| Checking {3-15} | 6.4 (3.2) | 6.2 (2.7) | 7.3 (3.1) |
| Ordering {5-25} | 8.4 (4.6) | 6.9 (2.4) | 9.0 (4.2) |
| Cleaning {7-35} | 10.0 (4.8) | 10.5 (4.6) | 11.9 (5.7) |
| **IUS-12*** {12-60} | 37.7 (13.0) | 37.0 (11.7) | 33.9 (11.4) |
| **RTQ-10*** {10-50} | 34.8 (7.2) | 32.4 (8.6) | 34.2 (11.2) |
| **HR (bpm)*** | | | |
| Free Breathing | 67.8 (8.9) | 70.2 (11.3) | 72.6 (15.6) |
| Paced Breathing | 68.8 (9.0) | 73.4 (12.1) | 71.9 (15.2) |
| **IBI (ms)*** | | | |
| Free Breathing | 899.0 (117.1) | 874.0 (137.8) | 865.0 (196.5) |
| Paced Breathing | 886.8 (117.2) | 836.2 (128.7) | 870.8 (190.5) |
| **RMSSD (ms)*** | | | |
| Free Breathing | 41.3 (24.3) | 47.3 (24.3) | 38.4 (29.4) |
| Paced Breathing | 39.5 (22.0) | 43.4 (22.2) | 45.5 (30.0) |
| **LF (ms^2^)*** | | | |
| Free Breathing | 210.5 (70.8) | 198.4 (61.5) | 196.7 (99.9) |
| Paced Breathing | 190.9 (59.9) | 169.6 (52.4) | 195.0 (90.2) |
| **HF (ms^2^)*** | | | |
| Free Breathing | 82.8 (56.7) | 100.3 (69.4) | 75.2 (63.3) |
| Paced Breathing | 88.9 (53.4) | 96.9 (58.3) | 102.9 (85.0) |

*Note.* * = means (standard deviations). HR = heart rate. IBI = interbeat interval. RMSSD = Root Mean Square of Successive Differences in normal-to-normal heart beats. LF = low frequency heart rate variability; HF = high frequency heart rate variability. { } = subscale range. ms = milliseconds. bpm = beats per minute.

**Figure S1**

Probability of Direction (pd) of the Posterior Cohen’s d Difference Between ISF1 & Sham for the Hospital & Anxiety Scale – Anxiety Subscale (HADS-A)


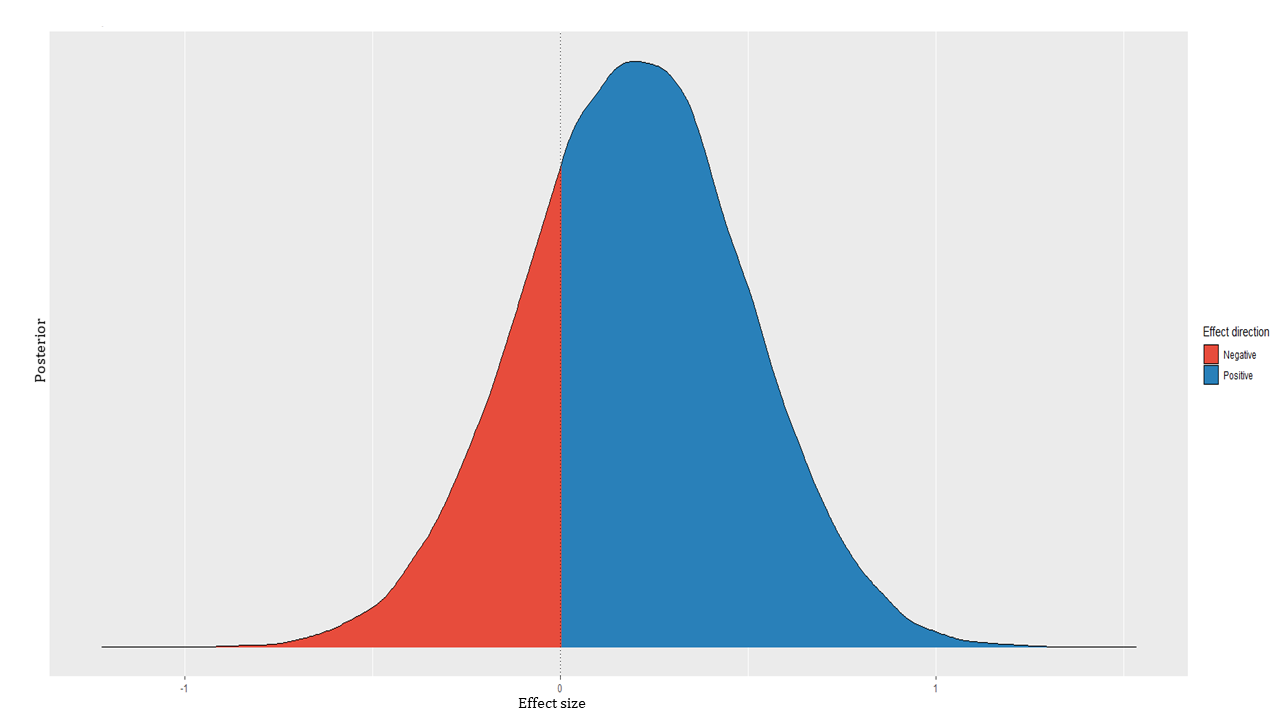


**Figure S2**

*Percentage In ±0.1 Region of Practical Equivalence (ROPE) of the Posterior Cohen’s d Difference Between ISF1 & Sham for the Hospital & Anxiety Scale – Anxiety Subscale (HADS-A)*


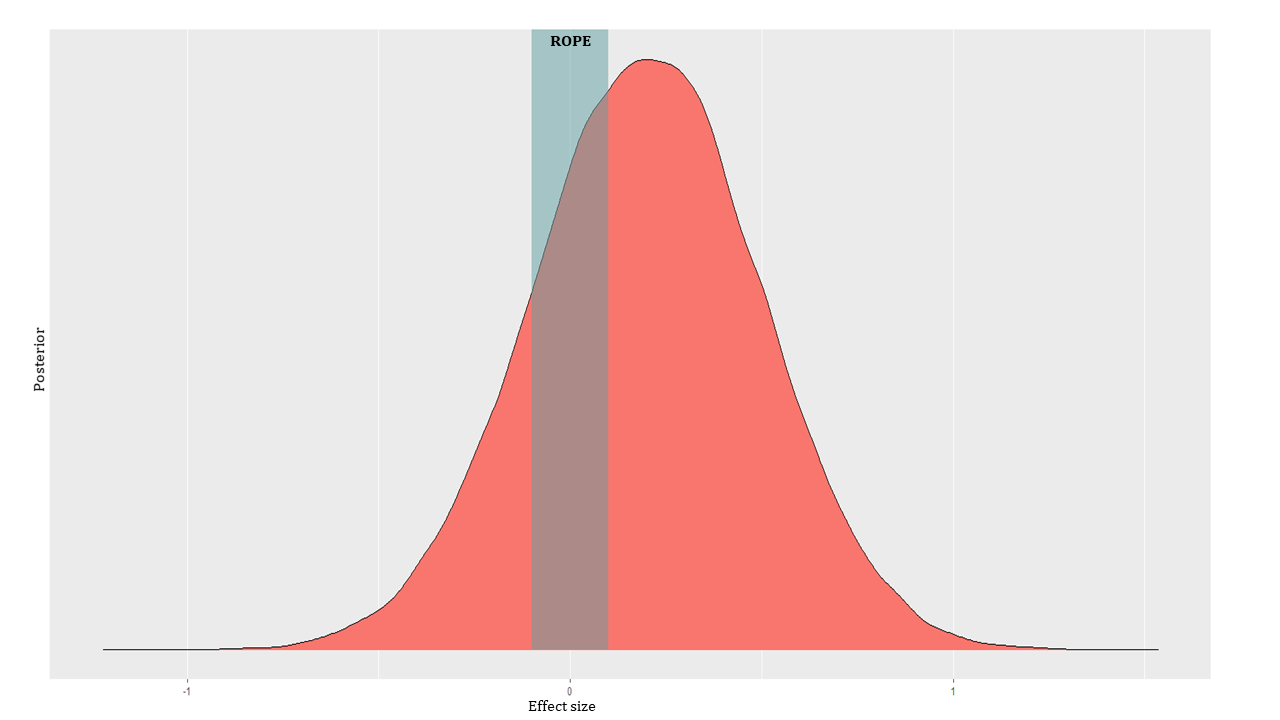


**Figure S3**

*Probability of Direction (pd) of the Posterior Cohen’s d Difference Between ISF1 & Sham for the Hospital & Anxiety Scale – Depression Subscale (HADS-D)*


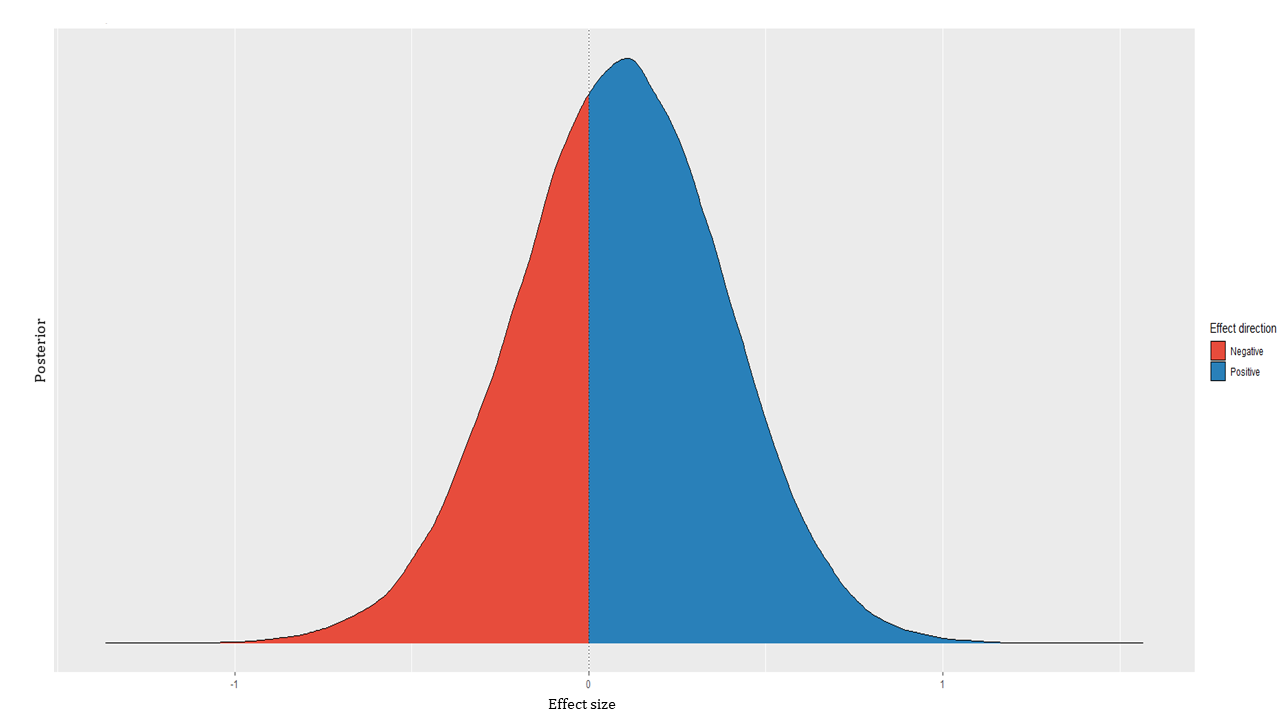


**Figure S4**

*Percentage In ±0.1 Region of Practical Equivalence (ROPE) of the Posterior Cohen’s d Difference Between ISF1 & Sham for the Hospital & Anxiety Scale – Depression Subscale (HADS-D)*


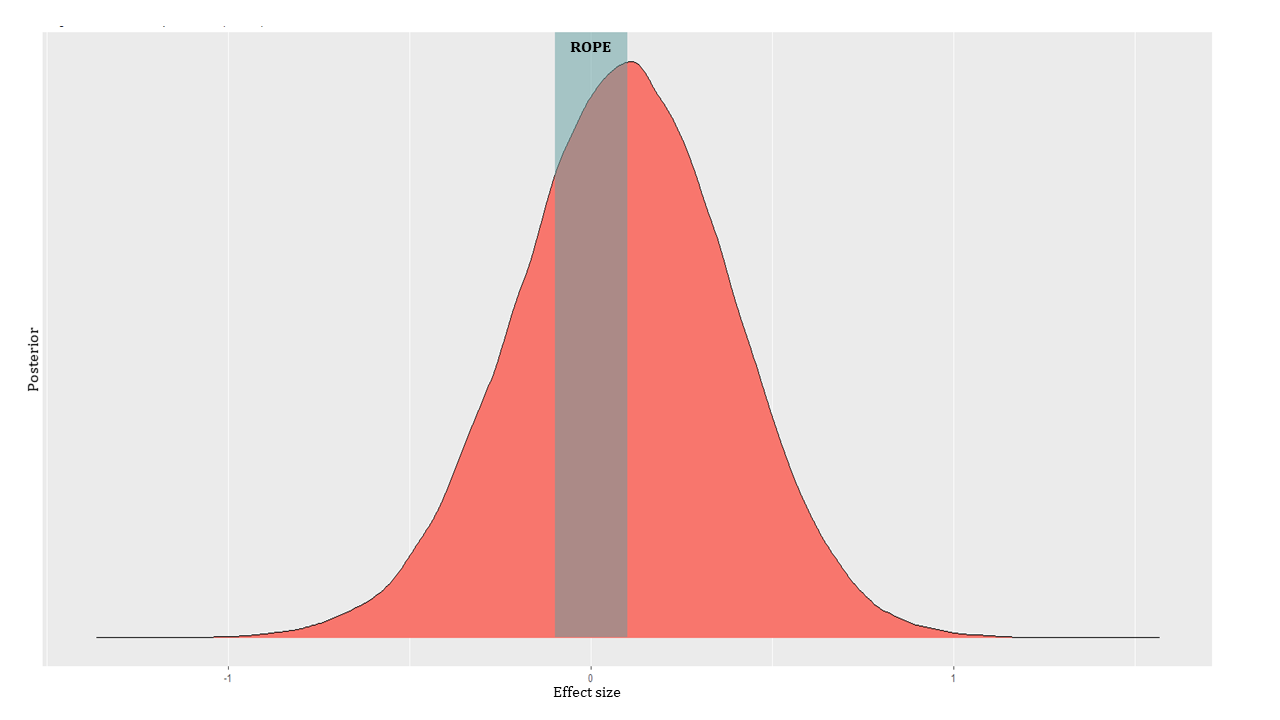


**Figure S5**

*Probability of Direction (pd) of the Posterior Cohen’s d Difference Between ISF2 & sham for the Hospital & Anxiety Scale – Anxiety Subscale (HADS-A)*


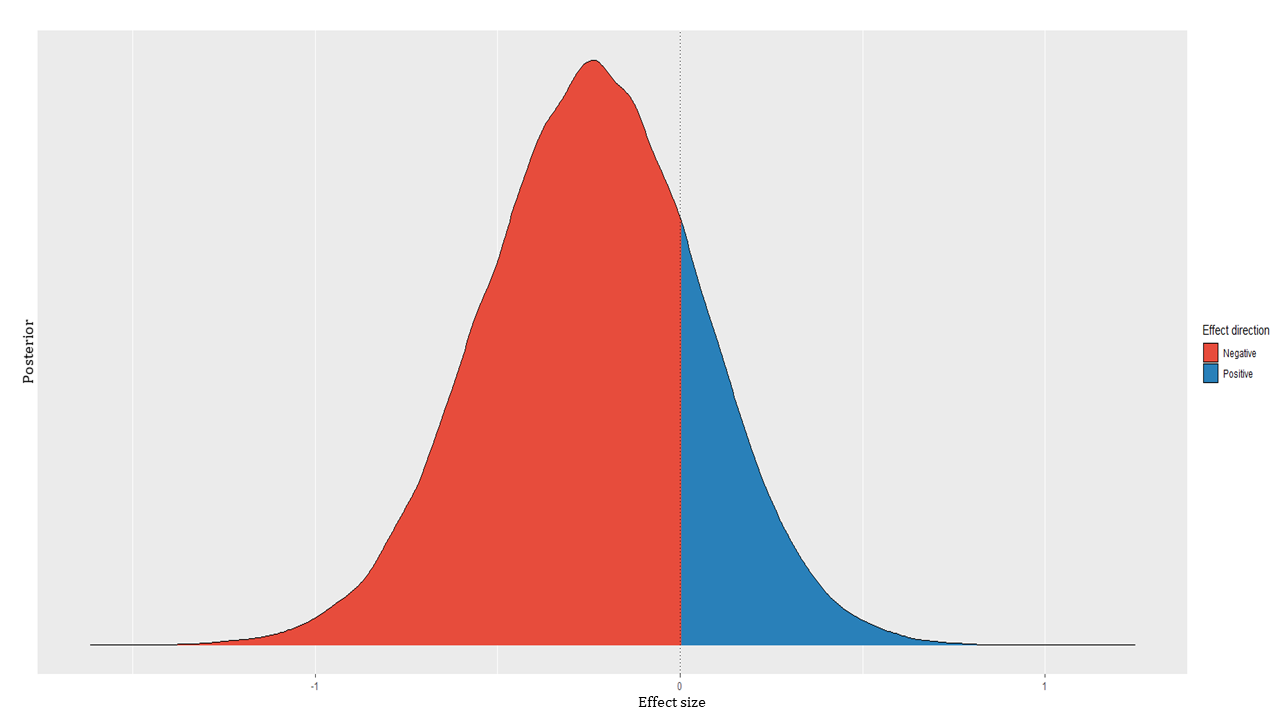


**Figure S6**

*Percentage In ±0.1 Region of Practical Equivalence (ROPE) of the Posterior Cohen’s d Difference Between ISF2 & Sham for the Hospital & Anxiety Scale – Anxiety Subscale (HADS-A)*


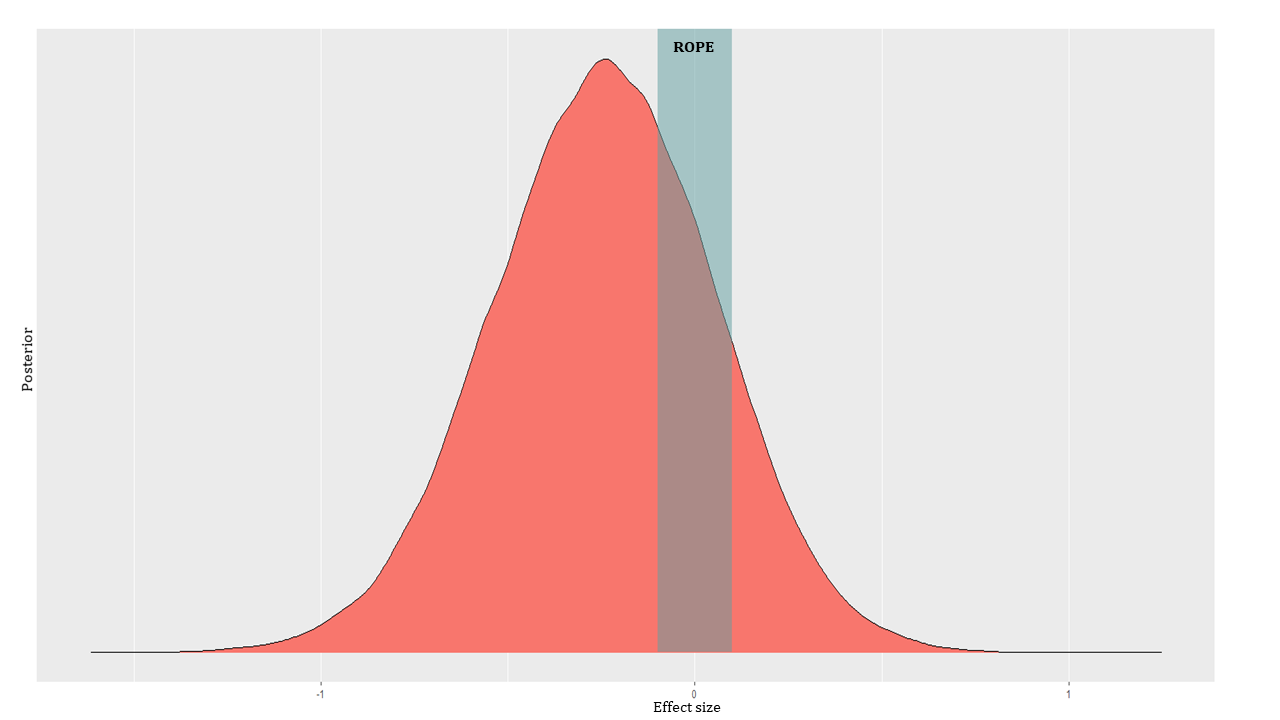


**Figure S7**

*Probability of Direction (pd) of the Posterior Cohen’s d Difference Between ISF2 & sham for the Hospital & Anxiety Scale – Depression Subscale (HADS-D)*


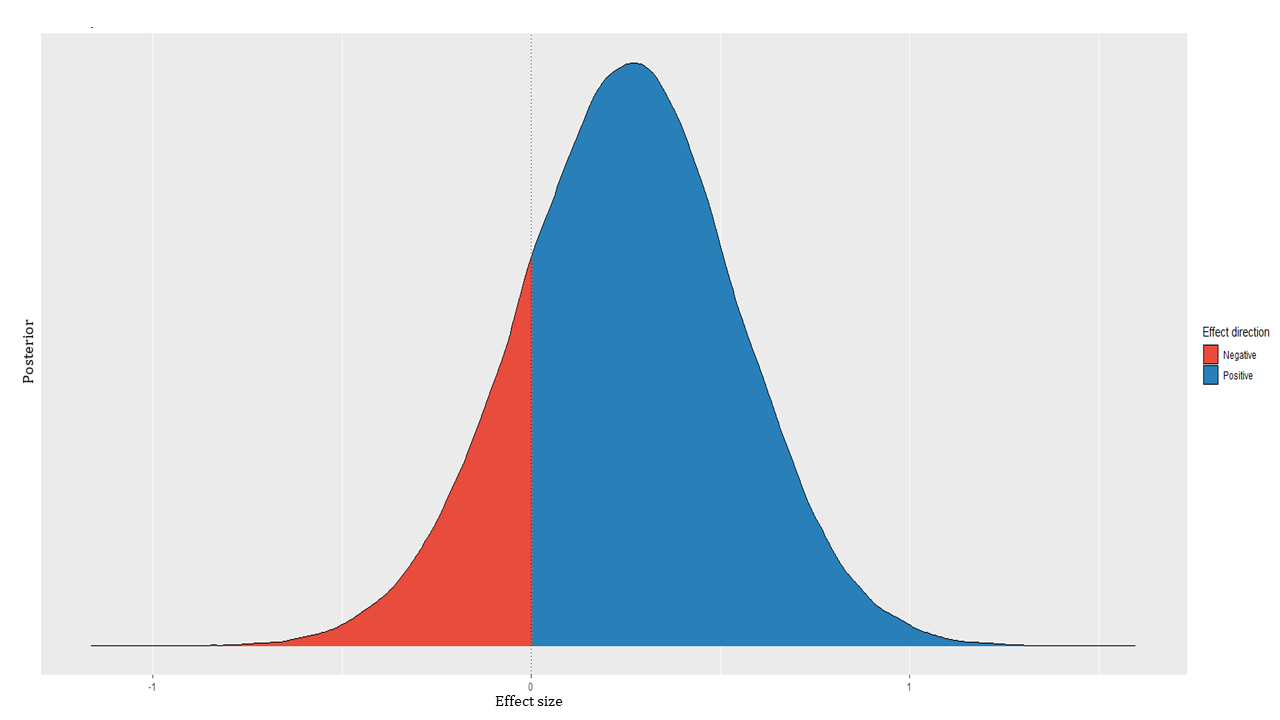


**Figure S8**

*Percentage In ±0.1 Region of Practical Equivalence (ROPE) of the Posterior Cohen’s d Difference Between ISF2 & Sham for the Hospital & Anxiety Scale – Depression Subscale (HADS-D)*


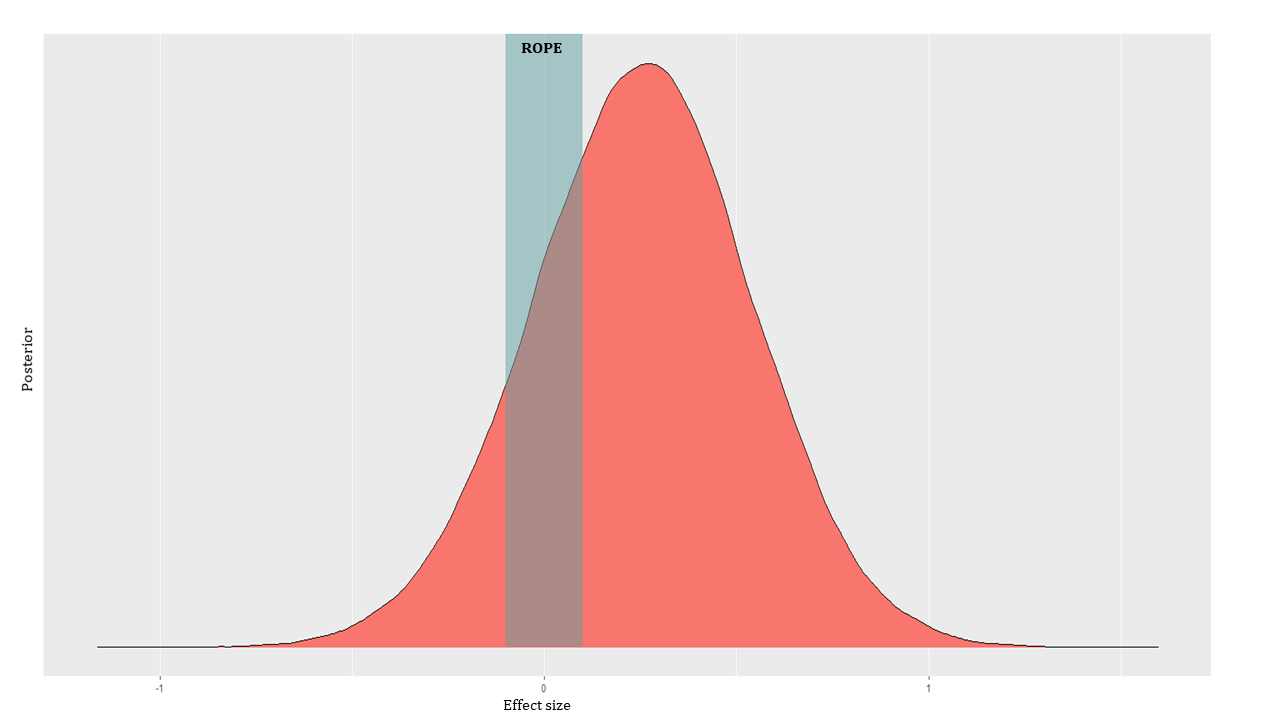


**Table S4**

*Secondary Patient Reported Outcome (PRO) Mean Change & Change Differences for Sham & ISF1*

| **PRO** (subscale) | **Sham d** *M* [95% HDI]  pd % in ±0.1 ROPE | **ISF1 d** *M* [95% HDI] pd  % in ±0.1 ROPE | **d difference** *M* [95% HDI] pd % in ±0.1 ROPE |
| --- | --- | --- | --- |
| **MEDI** | | | |
| Neurotic Temperament  (MEDI-NT) | -0.41 [-0.81, 0.00] 97.8% 5.8% | -0.59 [-1.12, -0.06] 98.5% 3.1% | -0.17 [-0.82, 0.47] 70.0% 20.9% |
| Positive Temperament  (MEDI-PT) | 0.29 [-0.10, 0.70] 92.2% 14.5% | 0.06 [-0.46, 0.58] 58.6% 28.7% | -0.23 [-0.90, 0.43] 75.6% 18.0% |
| Depression  (MEDI-Dep) | -0.34 [-0.71, 0.03] 96.4% 9.0% | -0.61 [-1.09, -0.12] 99.4% 1.7% | -0.27 [-0.84, 0.30] 82.3% 17.9% |
| Social Concerns  (MEDI-Soc) | -0.78 [-1.20, -0.37] >99.9% <0.1% | -0.48 [-1.00, 0.03] 96.7% 6.0% | 0.30 [-0.31, 0.95] 82.8% 15.9% |
| Autonomic Arousal  (MEDI-AA) | -0.60 [-0.96, -0.24] >99.9% 0.3% | -0.74 [-1.19, -0.29] >99.9% 0.2% | -0.14 [-0.61, 0.33] 71.7% 28.3% |
| Somatic Anxiety  (MEDI-SA) | -0.05 [-0.44, 0.34] 59.2% 37.9% | -0.63 [-1.17, -0.10] 99.1% 2.1% | -0.59 [-1.24, 0.05] 96.3% 5.1% |
| Intrusive Cognition  (MEDI-IC) | -0.38 [-0.77, 0.01] 97.1% 7.2% | -0.70 [-1.24, -0.18] 99.5% 1.1% | -0.32 [-0.95, 0.33] 83.7% 15.7% |
| Traumatic Reexperiencing  (MEDI-TR) | -0.33 [-0.70, 0.04] 96.0% 10.1% | -0.31 [-0.80, 0.17] 89.8% 14.8% | 0.02 [-0.56, 0.60] 52.7% 26.8% |
| Avoidance  (MEDI-Avoid) | -0.36 [-0.74, 0.03] 96.4% 8.5% | -0.54 [-1.04, -0.04] 98.2% 3.9% | -0.18 [-0.81, 0.42] 71.6% 21.8% |
| **IDAS-II** | | | |
| General Depression  (IDAS-Dep) | -0.87 [-1.29, -0.45] >99.9% <0.1% | -0.98 [-1.53, -0.43] >99.9% <0.1% | -0.11 [-0.76, 0.56] 62.7% 22.0% |
| Social Anxiety  (IDAS-Soc) | -0.61 [-1.02, -0.20] 99.8% 0.8% | -0.34 [-0.86, 0.18] 90.1% 13.3% | 0.27 [-0.38, 0.92] 79.3% 17.2% |
| Dysphoria  (IDAS-Dys) | -0.68 [-1.07, -0.27] >99.9% 0.2% | -0.74 [-1.27, -0.22] 99.8% 0.7% | -0.06 [-0.68, 0.56] 57.6% 24.7% |
| Lassitude  (IDAS-Las) | -0.52 [-0.92, -0.13] 99.5% 1.8% | -0.37 [-0.87, 0.15] 92.6% 11.2% | 0.15 [-0.47, 0.77] 68.2% 22.3% |
| Insomnia  (IDAS-Ins) | -0.51 [-0.91, -0.12] 99.4% 2.0% | -0.52 [-1.04, -0.02] 97.6% 4.5% | -0.01 [-0.62, 0.62] 50.9% 24.9% |
| Appetite Loss  (IDAS-AL) | -0.51 [-0.89, -0.13] 99.6% 1.6% | -0.25 [-0.73, 0.22] 85.0% 19.6% | 0.26 [-0.31, 0.83] 81.6% 18.3% |
| Appetite Gain  (IDAS-AG) | -0.24 [-0.63, 0.15] 88.5% 20.0% | -0.53 [-1.03, -0.01] 98.0% 4.3% | -0.29 [-0.94, 0.31] 82.2% 16.6% |
| Well-Being  (IDAS-WB) | 0.22 [-0.15, 0.61] 87.6% 21.7% | 0.18 [-0.30, 0.69] 76.4% 24.4% | -0.04 [-0.63, 0.56] 56.1% 25.8% |
| Ill Temper  (IDAS-Ill) | -0.47 [-0.82, -0.11] 99.5% 2.0% | -0.36 [-0.79, 0.08] 94.9% 10.2% | 0.10 [-0.39, 0.59] 66.6% 28.5% |
| Traumatic Intrusions  (IDAS-TI) | -0.28 [-0.66, 0.09] 92.7% 15.2% | -0.36 [-0.85, 0.13] 92.9% 11.6% | -0.09 [-0.67, 0.49] 61.1% 25.4% |
| Traumatic Avoidance  (IDAS-TA) | -0.68 [-1.06, -0.31] >99.9% 0.1% | -0.24 [-0.69, 0.21] 85.2% 20.5% | 0.44 [-0.08, 0.99] 94.9% 8.0% |
| Panic  (IDAS-Pan) | -0.85 [-1.21, -0.49] >99.9% <0.1% | -0.52 [-0.92, -0.12] 99.5% 1.8% | 0.33 [-0.09, 0.74] 94.3% 11.5% |
| Suicidality  (IDAS-Sui) | -0.32 [-0.70, 0.07] 94.4% 11.9% | -0.22 [-0.72, 0.28] 80.1% 21.8% | 0.10 [-0.53, 0.71] 62.8% 23.7% |
| Mania  (IDAS-Man) | -0.30 [-0.68, 0.08] 93.6% 13.6% | -0.20 [-0.69, 0.29] 79.1% 22.9% | 0.10 [-0.49, 0.69] 62.5% 25.0% |
| Euphoria  (IDAS-Eup) | 0.26 [-0.13, 0.63] 90.8% 17.6% | -0.01 [-0.49, 0.47] 51.9% 31.9% | -0.27 [-0.85, 0.31] 82.0% 17.6% |
| Claustrophobia  (IDAS-Clau) | -0.45 [-0.79, -0.11] 99.5% 2.0% | -0.51 [-0.94, -0.08] 99.1% 2.7% | -0.05 [-0.50, 0.40] 59.2% 33.9% |
| Checking  (IDAS-Check) | -0.43 [-0.82, -0.03] 98.3% 4.8% | -0.42 [-0.93, 0.09] 94.5% 8.8% | 0.01 [-0.62, 0.63] 50.7% 24.6% |
| Ordering  (IDAS-Ord) | -0.09 [-0.47, 0.30] 67.8% 35.0% | -0.21 [-0.71, 0.29] 79.7% 21.7% | -0.12 [-0.75, 0.49] 65.0% 22.9% |
| Cleaning  (IDAS-Clean) | -0.50 [-0.89, -0.11] 99.5% 2.1% | -0.25 [-0.73, 0.24] 84.1% 19.5% | 0.26 [-0.34, 0.86] 80.0% 18.0% |
| **IUS-12** | -0.22 [-0.61, 0.17] 86.6% 21.8% | -0.21 [-0.72, 0.28] 80.1% 21.9% | 0.01 [-0.62, 0.60] 50.7% 25.2% |
| **RTQ-10** | -0.29 [-0.67, 0.09] 93.5% 13.8% | -0.37 [-0.85, 0.11] 93.5% 10.5% | -0.08 [-0.65, 0.50] 60.9% 25.5% |

*Note*. Light blue cell = probably significant reduction. Dark blue cell = certainly significant reduction. ISF1 = 1-region infraslow neurofeedback. *M* = mean. HDI = highest density interval. pd = probability of direction. % in ±0.1 ROPE = percentage of the posterior in the ±0.1 region of practical equivalence. d = Cohen’s d.

**Table S5**

*Secondary Patient Reported Outcome (PRO) Mean Change & Change Differences for Sham & ISF2*

| **PRO** (subscale) | **Sham d** *M* [95% HDI]  pd % in ±0.1 ROPE | **ISF2 d** *M* [95% HDI] pd  % in ±0.1 ROPE | **d difference** *M* [95% HDI] pd % in ±0.1 ROPE |
| --- | --- | --- | --- |
| **MEDI** | | | |
| Neurotic Temperament  (MEDI-NT) | -0.41 [-0.81, 0.00] 97.8% 5.8% | -0.63 [-1.14, -0.12] 99.3% 1.7% | -0.22 [-0.86, 0.40] 75.9% 19.5% |
| Positive Temperament  (MEDI-PT) | 0.29 [-0.10, 0.70] 92.2% 14.5% | -0.12 [-0.61, 0.37] 68.9% 27.7% | -0.41 [-1.04, 0.23] 90.0% 10.5% |
| Depression  (MEDI-Dep) | -0.34 [-0.71, 0.03] 96.4% 9.0% | -0.82 [-1.30, -0.33] >99.9% 0.1% | -0.48 [-1.04, 0.09] 95.3% 7.3% |
| Social Concerns  (MEDI-Soc) | -0.78 [-1.20, -0.37] >99.9% <0.1% | -0.58 [-1.09, -0.09] 98.9% 2.5% | 0.20 [-0.43, 0.80] 73.8% 20.4% |
| Autonomic Arousal  (MEDI-AA) | -0.60 [-0.96, -0.24] >99.9% 0.3% | -0.78 [-1.20, -0.35] >99.9% <0.1% | -0.18 [-0.62, 0.29] 77.7% 25.3% |
| Somatic Anxiety  (MEDI-SA) | -0.05 [-0.44, 0.34] 59.2% 37.9% | -0.64 [-1.14, -0.13] 99.4% 1.6% | -0.59 [-1.20, 0.04] 97.0% 4.5% |
| Intrusive Cognition  (MEDI-IC) | -0.38 [-0.77, 0.01] 97.1% 7.2% | -0.46 [-0.95, 0.02] 96.7% 6.1% | -0.08 [-0.68, 0.52] 59.6% 24.6% |
| Traumatic Reexperiencing  (MEDI-TR) | -0.33 [-0.70, 0.04] 96.0% 10.1% | -0.36 [-0.81, 0.09] 94.1% 10.8% | -0.03 [-0.57, 0.51] 54.2% 28.3% |
| Avoidance  (MEDI-Avoid) | -0.36 [-0.74, 0.03] 96.4% 8.5% | -0.57 [-1.07, -0.08] 98.8% 2.8% | -0.21 [-0.82, 0.38] 75.5% 20.5% |
| **IDAS-II** | | | |
| General Depression  (IDAS-Dep) | -0.87 [-1.29, -0.45] >99.9% <0.1% | -1.28 [-1.85, -0.70] >99.9% <0.1% | -0.41 [-1.07, 0.25] 89.0% 11.4% |
| Social Anxiety  (IDAS-Soc) | -0.61 [-1.02, -0.20] 99.8% 0.8% | -0.66 [-1.18, -0.14] 99.4% 1.5% | -0.05 [-0.70, 0.59] 56.1% 23.5% |
| Dysphoria  (IDAS-Dys) | -0.68 [-1.07, -0.27] >99.9% 0.2% | -1.05 [-1.58, -0.49] >99.9% <0.1% | -0.37 [-1.01, 0.27] 87.4% 13.3% |
| Lassitude  (IDAS-Las) | -0.52 [-0.92, -0.13] 99.5% 1.8% | -0.73 [-1.23, -0.22] 99.8% 0.6% | -0.21 [-0.83, 0.39] 75.0% 20.6% |
| Insomnia  (IDAS-Ins) | -0.51 [-0.91, -0.12] 99.4% 2.0% | -0.65 [-1.17, -0.15] 99.4% 1.5% | -0.14 [-0.76, 0.47] 67.0% 23.2% |
| Appetite Loss  (IDAS-AL) | -0.51 [-0.89, -0.13] 99.6% 1.6% | -0.37 [-0.83, 0.09] 94.4% 10.0% | 0.14 [-0.42, 0.69] 68.6% 24.9% |
| Appetite Gain  (IDAS-AG) | -0.24 [-0.63, 0.15] 88.5% 20.0% | -0.44 [-0.94, 0.05] 96.1% 7.1% | -0.21 [-0.83, 0.40] 74.5% 20.4% |
| Well-Being  (IDAS-WB) | 0.22 [-0.15, 0.61] 87.6% 21.7% | 0.11 [-0.37, 0.56] 67.7% 29.6% | -0.11 [-0.68, 0.46] 65.3% 25.0% |
| Ill Temper  (IDAS-Ill) | -0.47 [-0.82, -0.11] 99.5% 2.0% | -0.45 [-0.87, -0.01] 98.1% 4.8% | 0.02 [-0.46, 0.49] 53.5% 32.3% |
| Traumatic Intrusions  (IDAS-TI) | -0.28 [-0.66, 0.09] 92.7% 15.2% | 0.05 [-0.41, 0.51] 57.8% 32.4% | 0.32 [-0.24, 0.89] 87.0% 14.8% |
| Traumatic Avoidance  (IDAS-TA) | -0.68 [-1.06, -0.31] >99.9% 0.1% | -0.13 [-0.57, 0.31] 71.5% 29.5% | 0.55 [0.03, 1.08] 98.1% 3.6% |
| Panic  (IDAS-Pan) | -0.85 [-1.21, -0.49] >99.9% <0.1% | -0.69 [-1.10, -0.29] >99.9% 0.2% | 0.16 [-0.23, 0.57] 78.8% 28.1% |
| Suicidality  (IDAS-Sui) | -0.32 [-0.70, 0.07] 94.4% 11.9% | -0.51 [-1.00, -0.01] 98.0% 4.2% | -0.19 [-0.80, 0.42] 73.4% 21.2% |
| Mania  (IDAS-Man) | -0.30 [-0.68, 0.08] 93.6% 13.6% | -0.55 [-1.03, -0.07] 98.9% 2.9% | -0.25 [-0.83, 0.33] 80.7% 18.6% |
| Euphoria  (IDAS-Eup) | 0.26 [-0.13, 0.63] 90.8% 17.6% | 0.18 [-0.28, 0.66] 77.2% 24.9% | -0.08 [-0.68, 0.49] 60.3% 25.5% |
| Claustrophobia  (IDAS-Clau) | -0.45 [-0.79, -0.11] 99.5% 2.0% | -0.10 [-0.50, 0.29] 69.1% 34.3% | 0.35 [-0.09, 0.78] 94.7% 10.3% |
| Checking  (IDAS-Check) | -0.43 [-0.82, -0.03] 98.3% 4.8% | -0.35 [-0.84, 0.16] 91.7% 12.3% | 0.07 [-0.55, 0.69] 59.3% 24.3% |
| Ordering  (IDAS-Ord) | -0.09 [-0.47, 0.30] 67.8% 35.0% | -0.23 [-0.72, 0.26] 82.2% 21.0% | -0.14 [-0.75, 0.47] 67.2% 23.0% |
| Cleaning  (IDAS-Clean) | -0.50 [-0.89, -0.11] 99.5% 2.1% | -0.34 [-0.83, 0.13] 91.8% 12.7% | 0.16 [-0.41, 0.76] 70.7% 22.5% |
| **IUS-12** | -0.22 [-0.61, 0.17] 86.6% 21.8% | -0.61 [-1.09, -0.11] 99.2% 1.9% | -0.39 [-0.97, 0.24] 89.6% 11.6% |
| **RTQ-10** | -0.29 [-0.67, 0.09] 93.5% 13.8% | -0.44 [-0.90, 0.03] 97.0% 6.3% | -0.15 [-0.70, 0.43] 70.3% 24.0% |

*Note.* Light blue cell = probably significant reduction. Dark blue cell = certainly significant reduction. ISF2 = 2-region infraslow neurofeedback. *M* = mean. HDI = highest density interval. pd = probability of direction. % in ±0.1 ROPE = percentage of the posterior in the ±0.1 region of practical equivalence. d = Cohen’s d.

**Table S6**

*ECG-Related Activity Changes & Change Differences for Sham & ISF1*

| **ECG-related metric** | **Sham d** *M* [95% HDI] pd % in ±0.1 ROPE | **ISF1 d** *M* [95% HDI] pd % in ±0.1 ROPE | **d difference** *M* [95% HDI] pd % in ±0.1 ROPE |
| --- | --- | --- | --- |
| **HR** | | | |
| Free Breathing | 0.28 [-0.15, 0.71]  89.9% 16.6% | 0.29 [-0.31, 0.90]  82.9% 16.4% | 0.01 [-0.74, 0.72]  51.5% 21.4% |
| Paced Breathing | -0.01 [-0.43, 0.41] 51.8% 35.8% | 0.20 [-0.43, 0.81]  73.9% 20.2% | 0.21 [-0.52, 0.95]  71.6% 17.9% |
| **IBI** | | | |
| Free Breathing | -0.13 [-0.57, 0.30] 71.9% 29.7% | -0.21 [-0.80, 0.36] 76.3% 20.8% | -0.08 [-0.77, 0.63]  59.3% 21.3% |
| Paced Breathing | 0.14 [-0.30, 0.56]  74.3% 29.1% | -0.12 [-0.73, 0.46]  65.9% 24.1% | -0.27 [-0.98, 0.45]  76.8% 16.3% |
| **Ln-RMSSD** | | | |
| Free Breathing | -0.37 [-0.81, 0.05] 95.4% 9.3% | -0.22 [-0.84, 0.40]  75.4% 19.7% | 0.15 [-0.61, 0.89]  65.8% 19.2% |
| Paced Breathing | -0.09 [-0.51, 0.32]  67.3% 33.2% | -0.31 [-0.95, 0.30]  83.8% 15.6% | -0.22 [-0.94, 0.53]  72.0% 18.2% |
| **Ln-LF** | | | |
| Free Breathing | -0.18 [-0.60, 0.23]  80.9% 25.6% | 0.02 [-0.56, 0.60]  52.5% 26.4% | 0.20 [-0.49, 0.90]  71.7% 19.0% |
| Paced Breathing | 0.23 [-0.19, 0.64]  86.2% 20.7% | 0.05 [-0.57, 0.67]  55.9% 24.3% | -0.18 [-0.90, 0.58]  68.7% 18.4% |
| **Ln-HF** | | | |
| Free Breathing | -0.40 [-0.84, 0.02]  96.6% 7.3% | -0.42 [-1.04, 0.19] 91.1% 10.6% | -0.02 [-0.75, 0.70]  51.6% 21.6% |
| Paced Breathing | -0.07 [-0.48, 0.34]  63.2% 34.9% | -0.27 [-0.88, 0.35]  81.0% 17.2% | -0.20 [-0.90, 0.53]  71.1% 18.6% |

*Note.* ISF1 = 1-region infraslow neurofeedback. HR = heart rate. IBI = interbeat interval. Ln-RMSSD = natural log of root mean square of successive differences in normal-to-normal heart beats. Ln-LF = natural log of low frequency heart rate variability. Ln-HF = natural log of high frequency heart rate variability. *M* = mean. HDI = highest density interval. d = Cohen’s d.

**Table S7**

*ECG-Related Activity Changes & Change Differences for Sham & ISF2*

| **ECG-related metric** | **Sham d** *M* [95% HDI] pd % in ±0.1 ROPE | **ISF2 d** *M* [95% HDI] pd % in ±0.1 ROPE | **d difference** *M* [95% HDI] pd % in ±0.1 ROPE |
| --- | --- | --- | --- |
| **HR** | | | |
| Free Breathing | 0.28 [-0.15, 0.71]  89.9% 16.6% | 0.14 [-0.40, 0.67]  69.4% 25.0% | -0.14 [-0.84, 0.52] 65.8% 20.8% |
| Paced Breathing | -0.01 [-0.43, 0.41] 51.8% 35.8% | 0.27 [-0.26, 0.82]  84.2% 17.5% | 0.28 [-0.39, 0.94]  79.8% 16.4% |
| **IBI** | | | |
| Free Breathing | -0.13 [-0.57, 0.30] 71.9% 29.7% | -0.03 [-0.55, 0.49]  54.2% 29.5% | 0.10 [-0.54, 0.76]  61.7% 23.0% |
| Paced Breathing | 0.14 [-0.30, 0.56]  74.3% 29.1% | -0.15 [-0.68, 0.38]  71.1% 25.1% | -0.29 [-0.94, 0.36]  81.0% 16.2% |
| **Ln-RMSSD** | | | |
| Free Breathing | -0.37 [-0.81, 0.05] 95.4% 9.3% | 0.09 [-0.46, 0.62]  62.1% 27.0% | 0.46 [-0.25, 1.13]  90.2% 10.0% |
| Paced Breathing | -0.09 [-0.51, 0.32]  67.3% 33.2% | -0.12 [-0.66, 0.42]  67.2% 25.9% | -0.03 [-0.71, 0.62]  53.4% 23.7% |
| **Ln-LF** | | | |
| Free Breathing | -0.18 [-0.60, 0.23]  80.9% 25.6% | -0.13 [-0.64, 0.38]  69.0% 26.7% | 0.05 [-0.58, 0.70]  56.4% 24.2% |
| Paced Breathing | 0.23 [-0.19, 0.64]  86.2% 20.7% | -0.16 [-0.69, 0.39]  72.2% 23.8% | -0.39 [-1.04, 0.29]  87.6% 12.0% |
| **Ln-HF** | | | |
| Free Breathing | -0.40 [-0.84, 0.02]  96.6% 7.3% | 0.04 [-0.50, 0.56]  55.5% 28.6% | 0.44 [-0.23, 1.12]  90.1% 10.3% |
| Paced Breathing | -0.07 [-0.48, 0.34]  63.2% 34.9% | -0.15 [-0.68, 0.39]  71.3% 24.8% | -0.08 [-0.73, 0.56]  59.8% 23.2% |

*Note.* ISF2 = 2-region infraslow neurofeedback. HR = heart rate. IBI = interbeat interval. Ln-RMSSD = natural log of root mean square of successive differences in normal-to-normal heart beats. Ln-LF = natural log of low frequency heart rate variability. Ln-HF = natural log of high frequency heart rate variability. *M* = mean. HDI = highest density interval. d = Cohen’s d.

**Figure S9**

*Bar chart of the Discontinuation-Emergent Signs & Symptoms (DESS) scale*


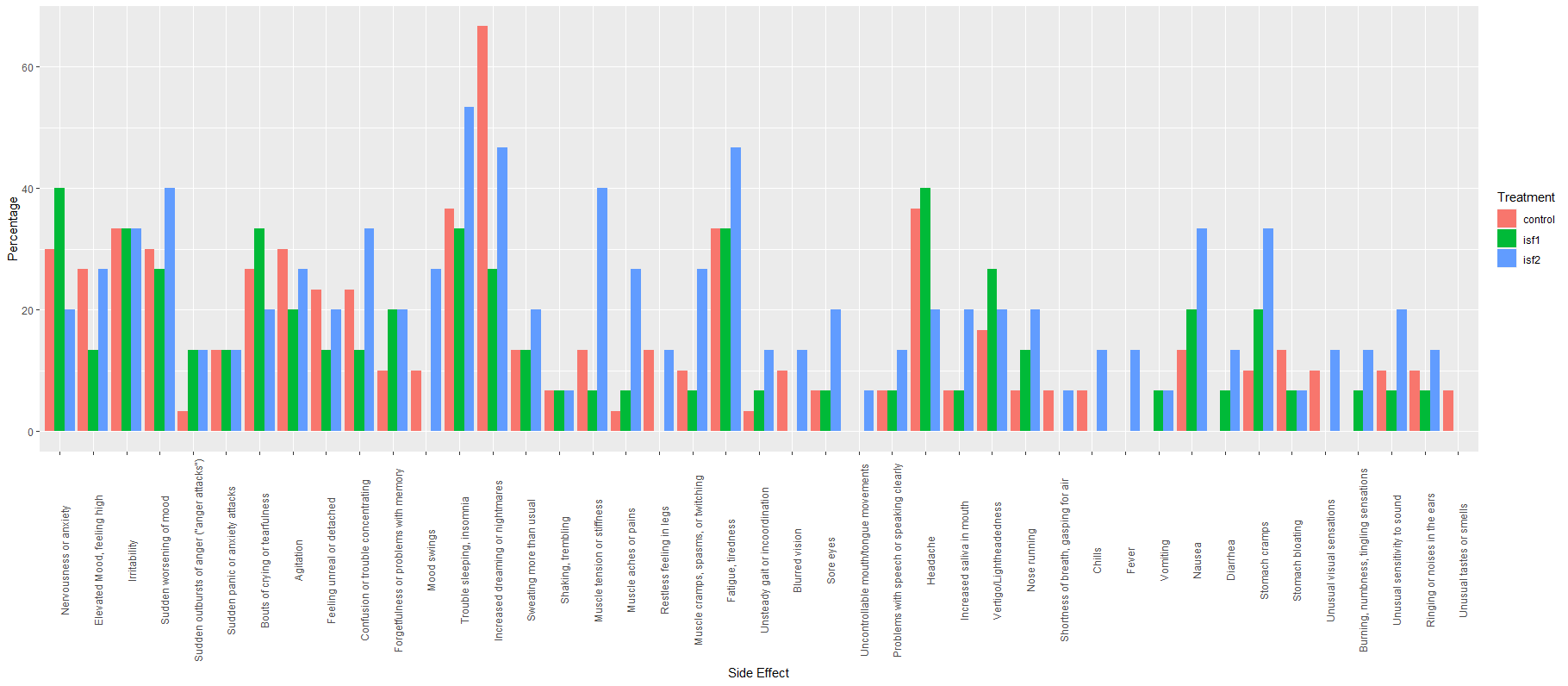


**Figure S10**

*Trace Plots of Samples from the Hospital Anxiety & Depression Scale – Depression Subscale (HADS-D) Posterior Cohen’s d for Sham (top), ISF1 (middle), & ISF2 (bottom)*


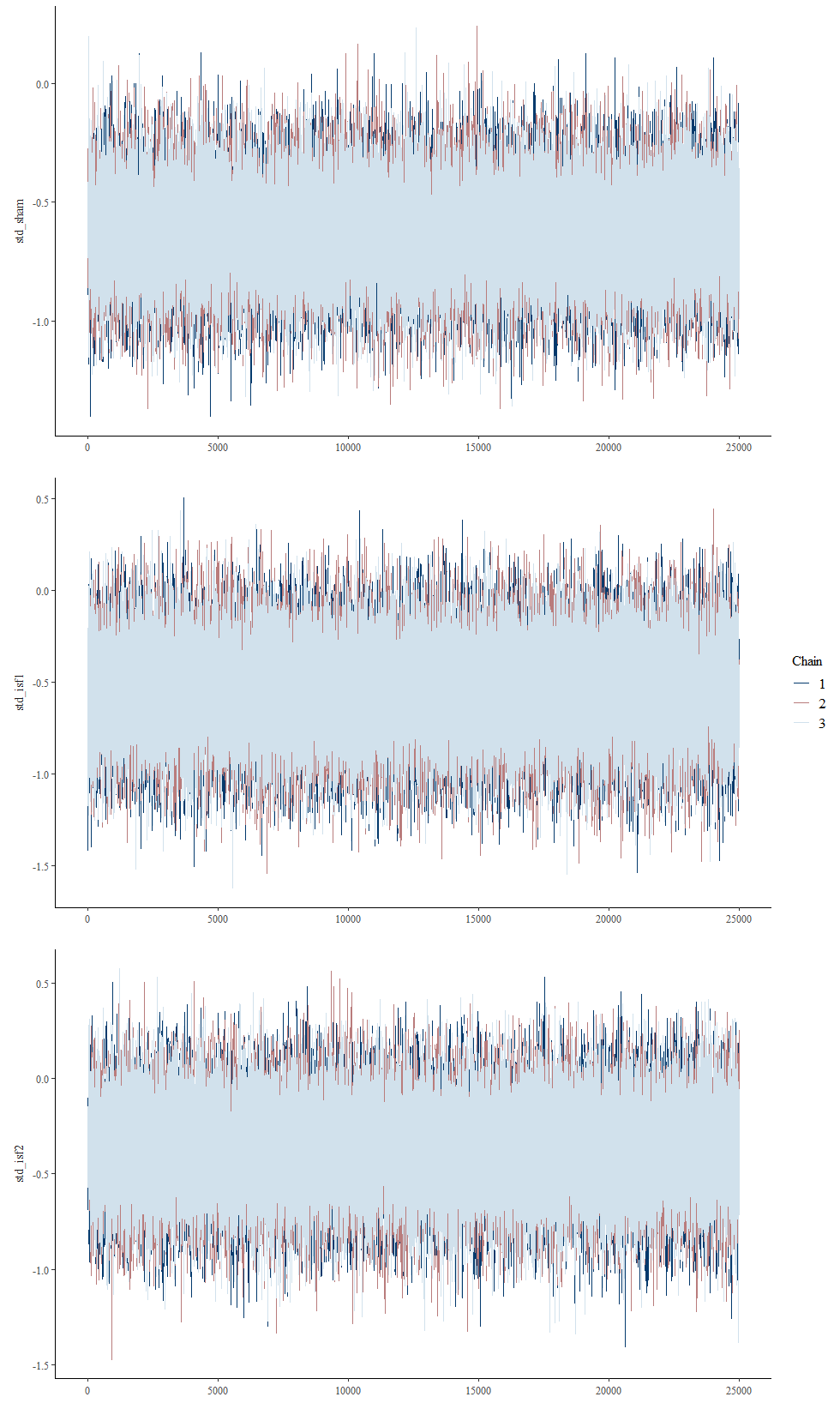


**Figure S11**

Comparison Plot of the Test Statistic (Sum of Squared Pearson Residuals) Derived From Observed (T2) & Predicted (T2.pred) Data for the Hospital & Anxiety Scale – Depression Subscale (HADS-D)
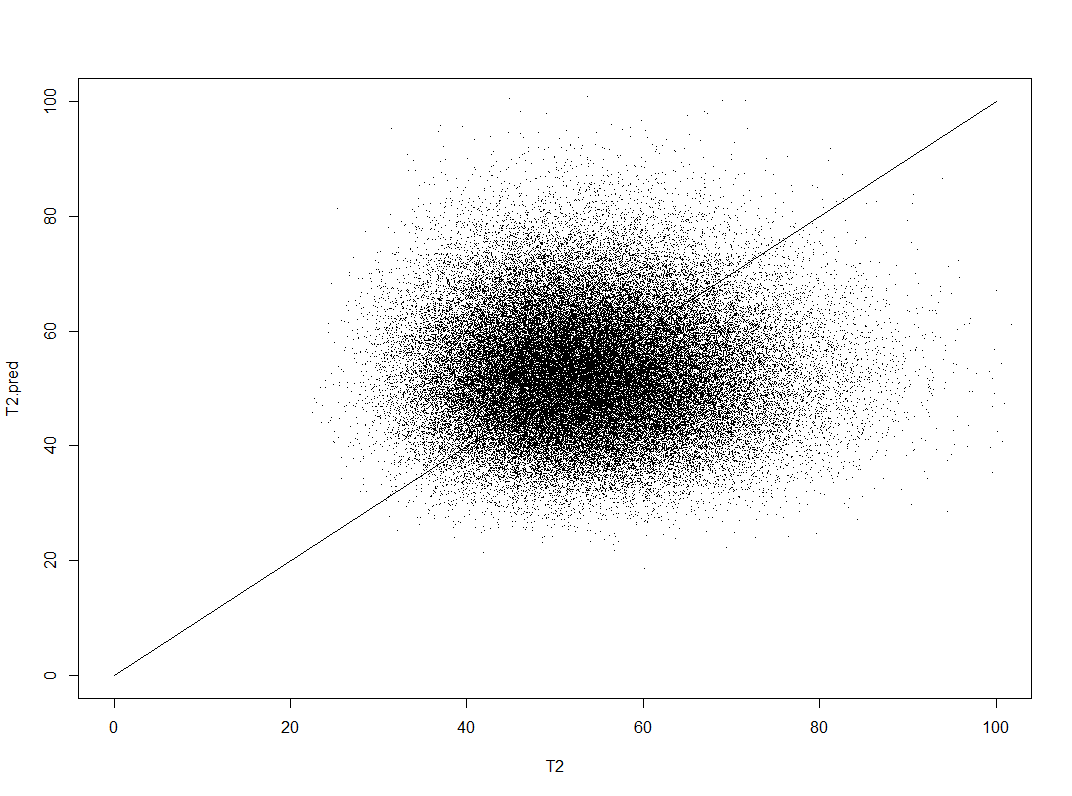


*Note.* Bayesian p-value = 0.47.

**Figure S12**

*Plots of the Standardized Residuals from the Hospital Anxiety & Depression Scale – Depression Subscale (HADS-D) for Sham (top), ISF1 (middle), ISF2 (bottom)*
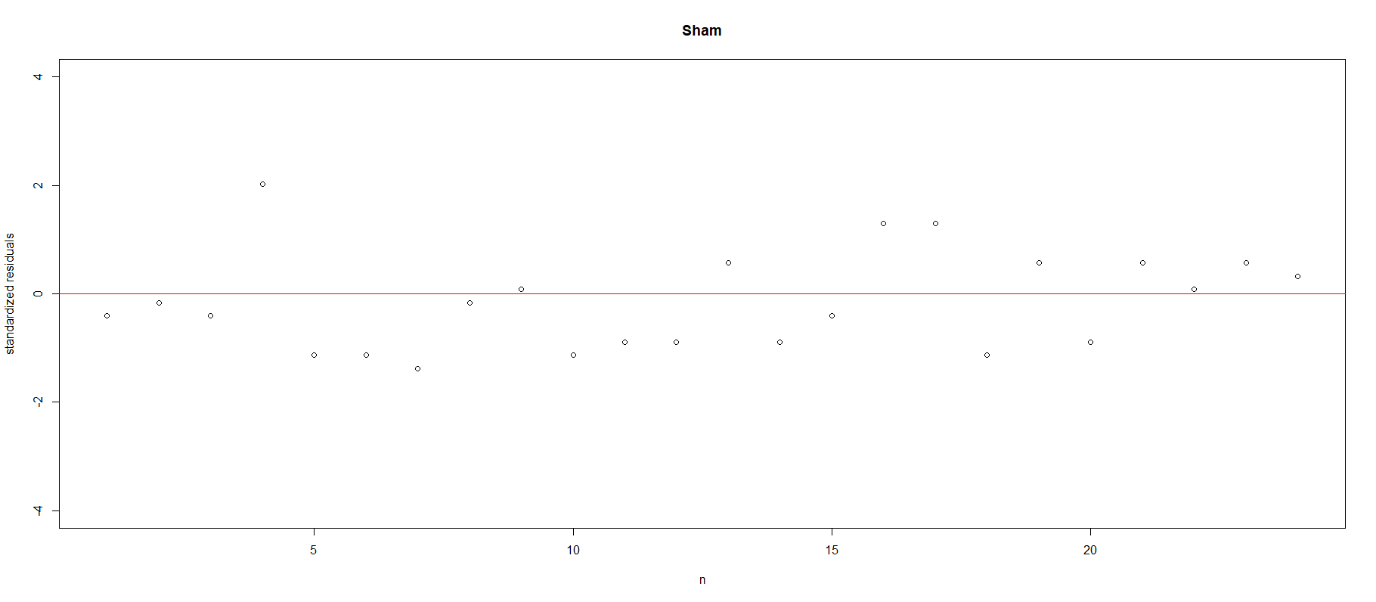

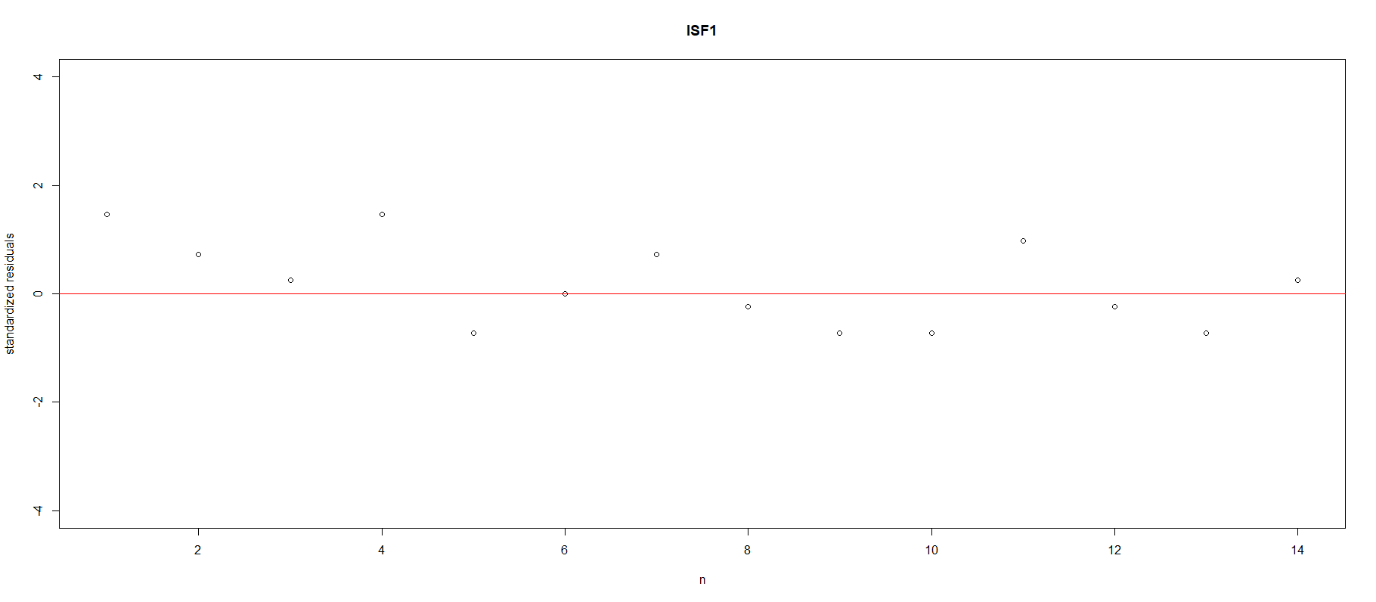


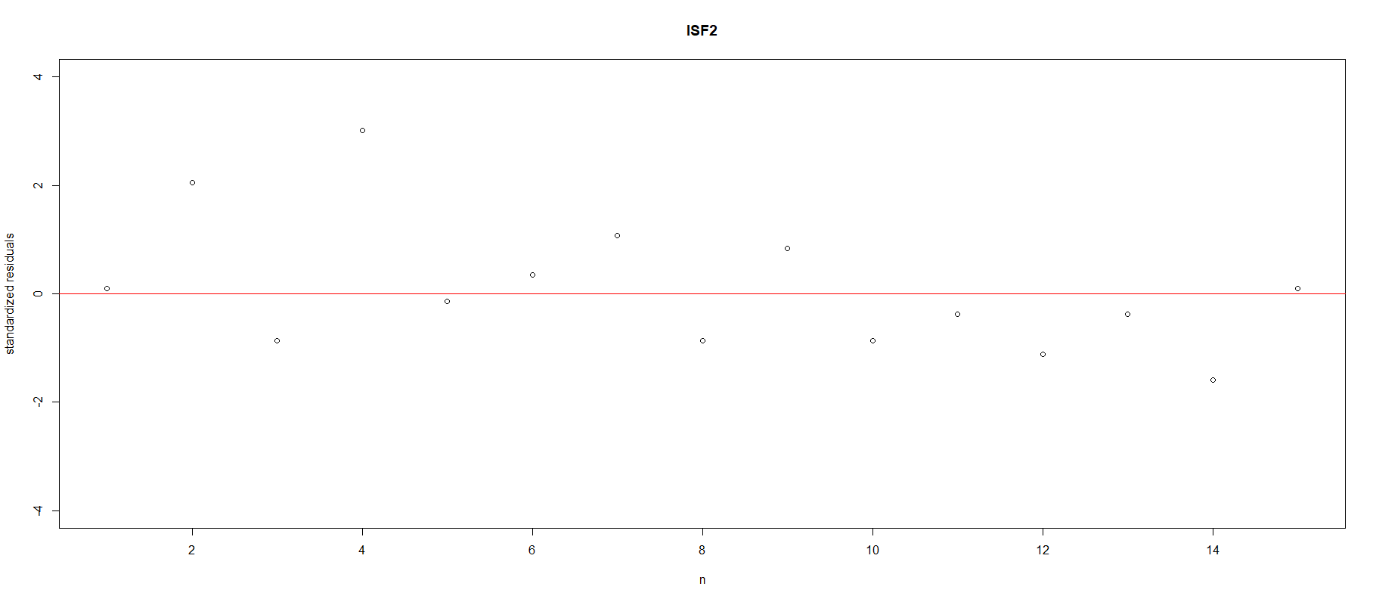


**Figure S13**

Quantile-quantile (Q-Q) plot of the Hospital Anxiety & Depression Scale – Depression Subscale (HADS-D) Standardized Residuals for Sham, ISF1, & ISF2
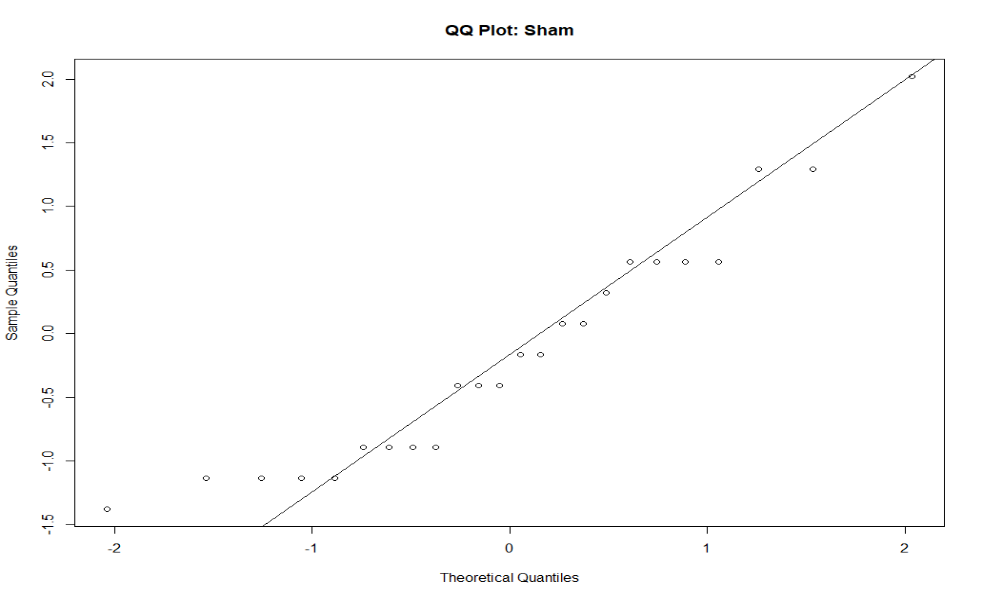

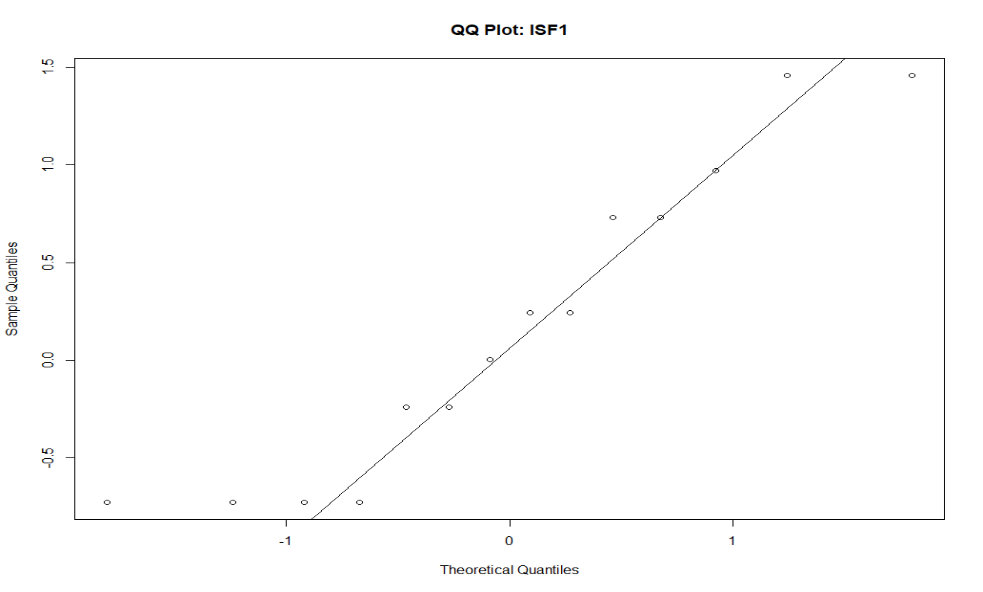

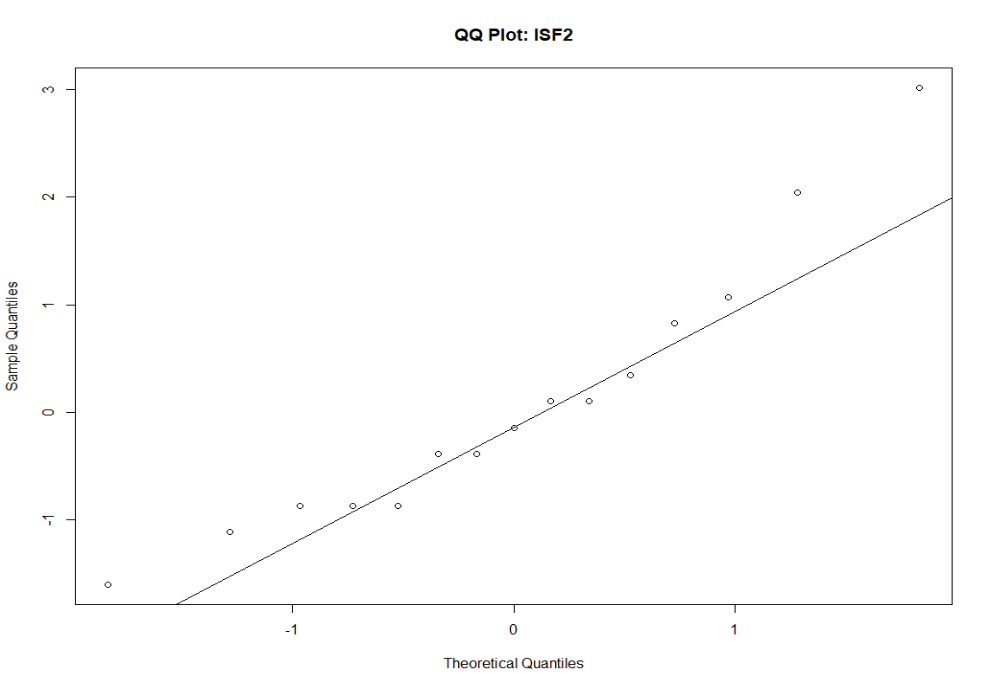

Supplement: Supplementary file 1 — Supplementary file1 (DOCX 12750 KB) [file 13415_2025_1279_MOESM1_ESM.docx]
